# Chemical Synthesis and Chaperone Peptide Mediated Folding of Human Nerve Growth Factor by Expressed KAHA Ligation

**DOI:** 10.1021/acscentsci.5c00277

**Published:** 2025-05-01

**Authors:** Nicolas Y. Nötel, Angus E. McMillan, Vijaya R. Pattabiraman, Katarina Vulić, Jeffrey W. Bode

**Affiliations:** † Laboratory of Organic Chemistry, Department of Chemistry and Applied Biosciences, 27219 ETH Zürich, 8093 Zürich, Switzerland; ‡ Laboratory of Biosensors and Bioelectronics, ETH Zürich, 8092 Zürich, Switzerland

## Abstract

Nerve growth factor (NGF) is a powerful neurotrophic protein for treating central nervous system diseases, but its therapeutic utility is limited by severe side effects, including hyperalgesia. These adverse effects arise from pleitropic receptor binding that can, in principle, be modulated by side chain mutations or modification—a task suited for chemical protein synthesis. Despite its small size (13 kDa), the chemical synthesis of NGF has been stymied by exceptional hydrophobicity and the requirement for a 104-residue N-terminal “chaperone peptide” for folding. This study presents a chemical synthesis of NGF using α-ketoacid-hydroxylamine (KAHA) ligations, featuring recombinant production of the chaperone peptide and its chemoselective conversion to a C-terminal α-ketoacid. A novel solubility tag, SOLACE, and ester-forming KAHA ligations enabled assembly of linear proNGF from three synthetic and one recombinant segment. Controlled folding and disulfide-bond formation mediated by the chaperone peptide followed by proteolytic cleavage yielded biologically active synthetic NGF as its noncovalent dimer. The synthetic NGF exhibited comparable activity to recombinant NGF in axon growth assays, establishing a platform for engineering NGF variants with tailored therapeutic properties. This approach provides a versatile framework for the semisynthesis of neurotrophins and related proteins that also require long chaperone peptides for proper folding.

## Graphical abstract

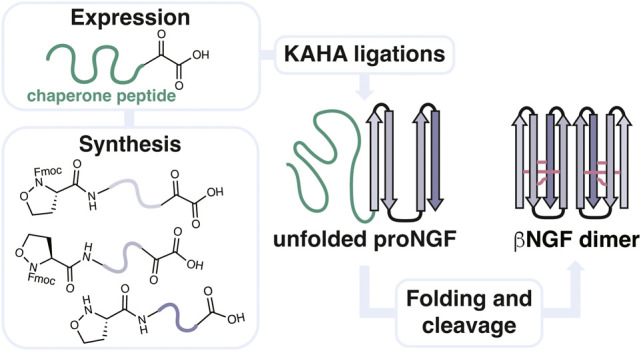

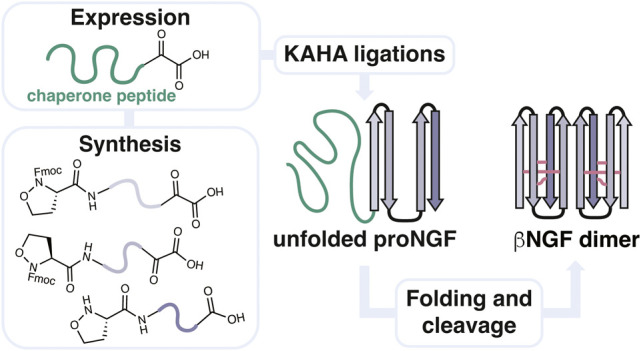

Nerve Growth Factor (NGF), a 13 kDa excreted neurotrophic protein that promotes neuron survival, proliferation, and differentiation, is a promising treatment for CNS diseases including Alzheimer’s disease, Parkinson’s disease, and hypoxic ischemic-perinatal brain injury.^1^ Unfortunately, NGF’s application is limited by a severe side effect, hyperalgesia—a heightened sensory sensitivity resulting in pain—stemming from NGF’s secondary role in priming sensory neurons.^2^ This has been partly circumvented by topical application allowing the approval of Oxervate, recombinant NGF eye drops, for the treatment of neurotrophic keratitis, but systemic administration of NGF therapies remains precluded.^3^

A path toward the uncoupling of NGF’s neurotrophic and sensitizing effects was revealed during the study of a Swedish family with a genetic condition with autonomic neuropathy type V (HSAN V).^4^ A point mutation in NGF (R221W) resulted in normal neurological development but reduced pain perception. Cattaneo et al. showed that an NGF variant (R221E) retained neurotrophic activity while reducing hyperalgesia in mice and SPR studies confirmed this mutation lowers NGF’s affinity for the p75 receptor without affecting its interaction with TrkA.^5,6^ Taken together, these insights suggest that TrkA-biased NGF variants could serve as painless NGF-based therapeutics for a wide range of neurologic conditions.

An ideal strategy for further advancing NGF as a therapeutic modality would involve atomic tailoring of side chains to enhance TrkA interactions while abating binding to p75, along with half-life extension and conditional activation approaches – in analogy to advances in cytokine engineering.^7^ To that end, a chemical synthesis of NGF would provide an ideal platform for such efforts, as it would facilitate both precise modulation of protein–protein interactions as well as chemical conjugation. While the relatively small size of NGF makes it appear, at first glance, to be well suited to this approach, its chemical synthesis is stymied by two considerations. First, NGF is an exceptionally hydrophobic protein, even more so than notoriously difficult synthetic targets including IL-2 and PD-1.^8–10^ Second, like all neurotrophins, NGF requires the presence of a long “chaperone peptide” at its N-terminus for proper folding and formation of its three disulfide bonds (Figure 1a).^11^ Any synthetic effort therefore requires the preparation of a 25 kDa proNGF protein that could be folded and processed to give active NGF variants.

**Figure 1:**
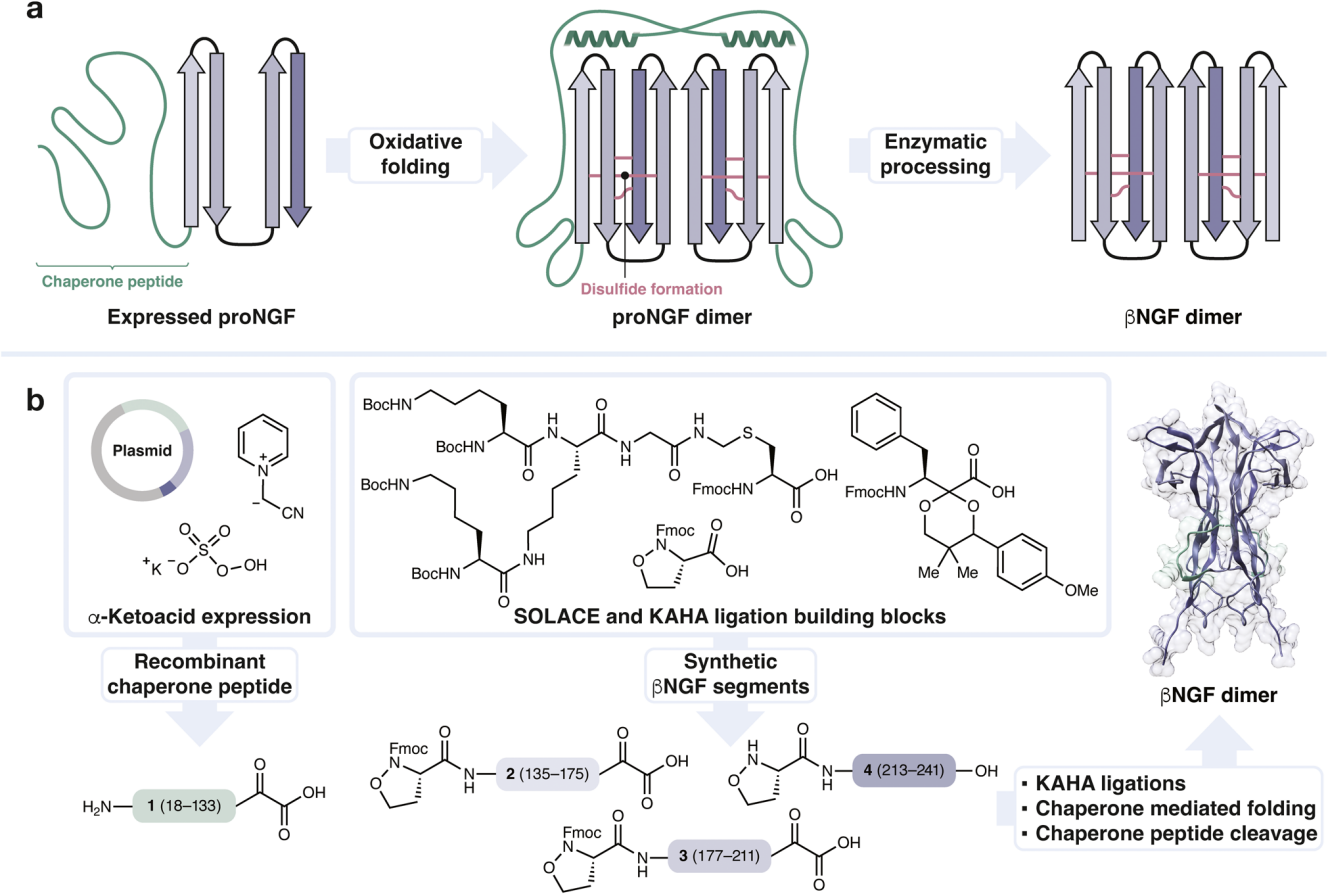
| NGF requires a chaperone peptide for folding. a, The chaperone peptide facilitates folding and is then enzymatically cleaved to produce mature βNGF dimer. b, Semisynthesis of NGF via KAHA ligation of the recombinant chaperone peptide with three synthetic segments comprising the mature NGF sequence, followed by oxidative folding and enzymatic processing to yield bioactive NGF. The βNGF dimer structure in this figure was generated with AlphaFold 3.^12^

Inspired by this challenge, we now document the successful synthesis of a biologically active NGF variant by α-ketoacid–hydroxylamine (KAHA) ligations of four segments, including a 116-residue chaperone peptide α-ketoacid prepared by recombinant expression (Figure 1b). The extremely hydrophobic nature of NGF was mitigated by ester-forming KAHA ligations with 5-oxaproline and the use of a new solubility-enhancing tag (SOLACE) attached to a Cys-residue of the most challenging peptide segment. Assembly of the four segments, followed by acyl shifts, chaperone peptide mediated folding, disulfide-formation, and selective removal of the unstructured chaperone peptide by controlled tryptic digestion afforded a biologically active synthetic NGF variant. Importantly, this work not only establishes a synthetic platform for preparing NGF variants but also documents the first example of a peptide-α-ketoacid prepared from a recombinantly expressed peptide, achieved by selective cleavage of an intein-derived thioester with a cyanopyridinium ylide and its chemoselective oxidation to the α-ketoacid.

## Results and Discussion

### Design and Retrosynthetic Analysis

ProNGF comprises 224 residues, with 18–121 serving as a “chaperone peptide” responsible for proper folding before being removed intracellularly by the protease furin.^13^ While we initially contemplated preparing just the sequence of active βNGF, Schwarz et al. has reported that efforts to fold the linear protein lacking the chaperone sequence were unsuccessful – a finding that we also confirmed during the course of our studies (see Supporting Information).^11^ A full chemical synthesis of proNGF, just to remove and discard nearly half of the protein, was unattractive, particularly as we did not plan to introduce any meaningful modifications or conjugation handles in this region.

We therefore sought to adopt the elegant concept of expressed chemical ligation introduced by Muir and co-workers and widely used for protein semisynthesis in conjunction with native chemical ligation. By expressing all or most of the chaperone peptide, we could avoid synthesis of this long segment and rely on bacterial fermentation for its production. At first glance, the presence of a Cys residue at position 136, near the N-terminus of wtNGF, endorsed this strategy. In practice, however, it was complicated by both the extreme hydrophobicity of mature NGF (122–241) and the requirement of NCL with a valine-derived thioester.^14^ We sought instead to convert the chaperone peptide–intein fusion to an α-ketoacid, thereby allowing the final ligation to occur under KAHA ligation conditions (HFIP/AcOH (2:1)) better suited for solubilizing and handling extremely hydrophobic systems. This also presented the opportunity to develop an “expressed KAHA ligation” approach to chemical proteins synthesis to complement the widely used expressed protein ligation of thioesters and N-terminal cysteine residues.

In considering the synthetic strategy toward active NGF proteins, we selected ligation sites that would enable us to introduce three homoserine mutations at Ser134, Glu176, and Thr212 (Figure 2a). We further introduced Met158Nle and Met213Nle mutations to mitigate undesired oxidation and replaced two acid-labile Asp-Pro sites (Asp73, Asp181)—one in the mature NGF sequence and one in the chaperone peptide—with the more stable Glu–Pro residues. In our initial studies on NGF synthesis, we observed partial cleavage at these sites during HPLC purification, consistent with prior findings by our group and others.^15,16^ The successful execution of the synthesis provides a roadmap for the production of fully synthetic NGF analogues.

**Figure 2:**
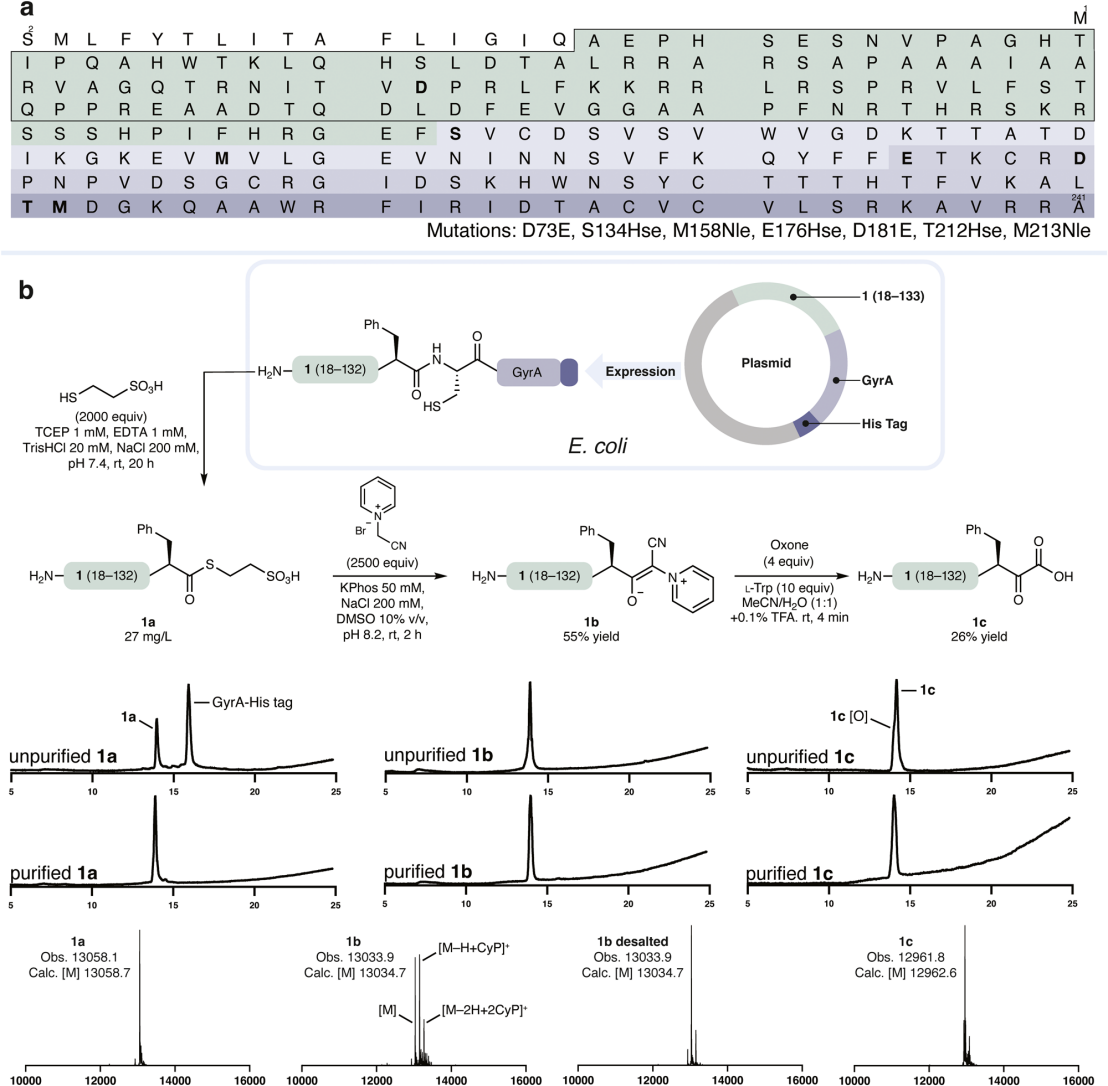
Recombinantly sourced α-ketoacid. a, One-letter amino acid sequence of proNGF (UniProt P01138) with segments indicated with color, mutation sites in bold, and outlined chaperone peptide. b, Chemical modification of recombinantly sourced segment **1** to produce a C-terminal α-ketoacid.

### Expression and Formation of the Chaperone-Peptide α-Ketoacid

We recombinantly expressed chaperone peptide **1** as a GyrA intein fusion in *E. coli*.^17^ As with previous reports of proNGF expression, the fusion protein was almost entirely sequestered in inclusion bodies, necessitating a denaturing extraction and refolding of the intein.^18^ Attempts to increase the amount of the soluble fraction by preparing an N-terminal SUMO fusion were largely unsuccessful.^19^ Following renaturation, the fusion protein was isolated using a His-tag on the C-terminus of the intein,^20^ and the intein fusion incubated with sodium 2-mercaptoethanesulfonate (MesNa) to induce thiolysis, providing recombinant thioester **1a** (27 mg/L, Figure 2b). Prior work from our group has established that C-terminal α-ketoacids can be prepared by chemoselective oxidation of C-terminal cyanosulfur ylides (CSYs), which themselves are installed by nucleophilic addition of an ylide to an activated amino acid. Initial studies using sulfur yildes successfully produced CSY, however, chemoselective oxidation of the 116-residue yilde proved unproductive.

Fortunately, treatment of **1a** with an excess of 1-(cyanomethyl)­pyridin-1-ium bromide at pH 8.2 gave clean conversion to cyanopyridinium ylide (CyPY) **1b**.^21^ The C-terminal CyPY **1b** was stable under basic, neutral, and mildly acidic aqueous conditions but hydrolyzed to the carboxylic acid under strongly acidic aqueous conditions. Interestingly, unreacted cyanopyridinium cations (CyP, *m*/*z* 118) proved to be unusually persistent, with [M–H+CyP]^+^ and [M–2H+2CyP]^+^ ions observed by mass spectrometry (MS) even after HPLC purification. While the subsequent oxidation was tolerant of the presence of the CyP salts, they could also be completely removed by dialysis in Milli-Q water. Treatment of **1b** with Oxone resulted in its oxidation to α-ketoacid **1c**. The choice of the CyPYs, rather than the analogous CSY previously employed by our group, was governed by the higher reactivity of the CyPY toward oxidants, allowing the transformation to proceed at reduced concentrations with a reduced amount of oxidant (830 μM CyPY, 4 equiv Oxone vs 5000 μM CSY, 20 equiv Oxone).^22^ The formation of a byproduct, identified by tryptic digestion and MS/MS to be the desired product with over oxidation at Trp37, could be minimized by the addition of sacrificial tryptophan (10 equiv). The reaction time and Oxone concentrations were also carefully tuned to minimize the formation of the C-terminal carboxylic acid, which may be produced by hydrolysis of the CyPY or oxidation of the α-ketoacid.

### Segment Synthesis and KAHA Ligation

Segments **2**, **3**, and **4** were produced on a sizable scale using standard Fmoc-SPPS methods with previously established monomers for installing the α-ketoacid (segment **3**) and (*S*)-5-hydroxyproline (segments **3**, **4**).^23^ The synthesis of the exceptionally hydrophobic and aggregation prone segment **2**, however, was challenging and prone to truncation, particularly at Cys136 and Asp137. The isolation of this segment was further complicated by its hydrophobicity (as evidenced by prolonged RP-HPLC retention time), initially resulting in an isolated yield of less than 2%. To overcome this issue, we developed SOLACE (SOLubility ACm Enhancer), a semipermanent solubility tag constructed from the cysteine protecting group acetamidomethyl (Acm) (Figure 3, see Supporting Information for synthetic route).^24^ The Fmoc-Cys­(SOLACE)-OH amino acid was directly incorporated at Cys136 using standard SPPS methods and has the apparent benefit of reducing truncation during SPPS in addition to improving the solubility of the peptide. Unlike previously reported semipermanent cysteine solubility tags, this approach requires no additional on-resin coupling steps or orthogonal protecting groups.^25,26^ With the incorporation of SOLACE, segment **2** was obtained in 13% yield.

**Figure 3:**
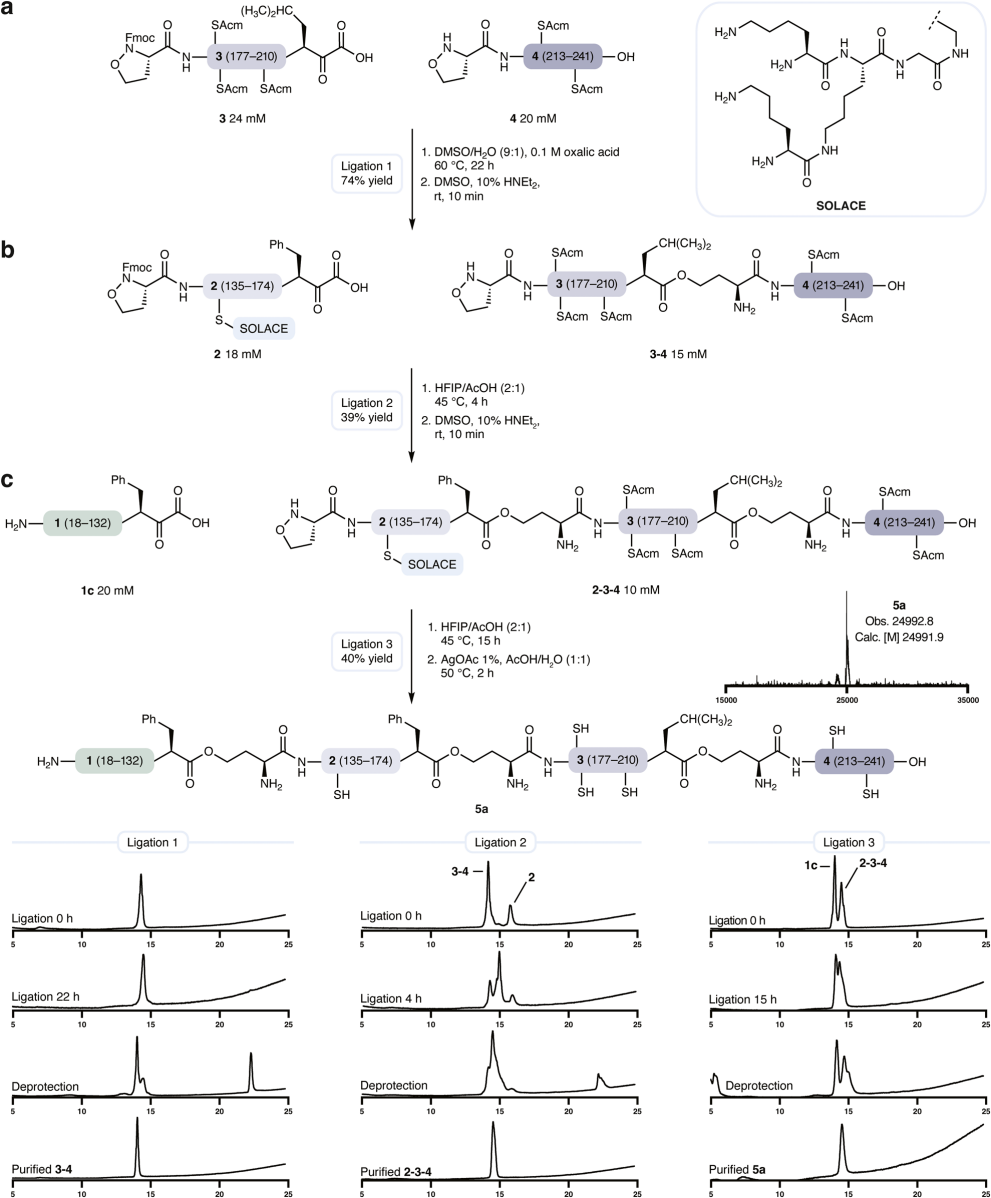
| Assembly of proNGF. a, KAHA ligation of segments **3** and **4** with one-pot Fmoc deprotection. b, KAHA ligation of segments **3–4** and **2** bearing SOLACE with one-pot Fmoc deprotection. c, KAHA ligation of recombinant segment **1c** and **2–3–4** with one-pot Acm and SOLACE removal.

Ligation of segments **3** and **4** proceeded well under standard KAHA ligation conditions for 5-oxaproline: DMSO/H_2_O (9:1), 0.1 M oxalic acid (Figure 3a). While the segments and the resulting ligation product were not initially separable by RP-HPLC, *in situ* Fmoc deprotection resolved the product and allowed for the isolation of **3–4** in 74% yield. The subsequent KAHA ligation with hydrophobic segment **2** benefited from the use of HFIP/AcOH (2:1) as solvent (Figure 3b) and one-pot Fmoc deprotection, affording linear NGF precursor **2–3–4** in 39%.

The union of synthetic **2–3–4** (108 residues) with recombinantly produced chaperon peptide α-ketoacid **1c** (116 residues) required a KAHA ligation of two 100+ residues segments – a feat never before accomplished with this reaction. Fortunately, conducting this ligation at 10 mM HFIP/AcOH (2:1) afforded the desired, 224 residue tri*depsi*-ligation product (Figure 3c). Subsequent one-pot Acm deprotection removed both SOLACE and the five Acm groups on the Cys residues to afford the 25 kDa *depsi*-proNGF **5a** in 40% yield after HPLC purification.

### Folding of Semisynthetic NGF

Previously established folding protocols for recombinant NGF, which we also expressed and folded, employ denaturing, reducing conditions at pH 8.0 prior to dialysis or dilution into folding buffers.^27^ These conditions are similar to those used for the rearrangement of depsipeptides (O–>N shift)^28^ produced by 5-oxaproline-based KAHA ligation,^29^ and we therefore sought to combine the denaturing and O–>N shift steps. Upon treating proNGF **5a** under these conditions (6 M GdnHCl, 100 mM TrisHCl, 100 mM EDTA, 1 mM DTT), we observed HPLC shifts consistent with O–>N rearrangement. Upon dialysis of this product, presumably the fully rearranged unfolded proNGF **5b**, into folding buffer (Figure 4) we observed a change in retention time that was in good agreement with our recombinant control. Folded, semisynthetic proNGF **5c** was isolated by RP-HPLC in 23% yield. Denaturing of the isolated material allowed for complete digestion with trypsin, and MS/MS analysis returned a 92% sequence coverage, confirming the correct assembly and rearrangements of semisynthetic proNGF. As previously noted,^30^ obtaining intact mass spectra of folded NGF proteins is challenging, for both recombinant and synthetic NGF. Prolonged desalting by dialysis was required before the formation of the cysteine knot could be confirmed by MS (see Supporting Information), where we clearly observed a mass difference of 6 Da, corresponding to the formation of three disulfide bridges, through the use of the MaxEntX deconvolution algorithm. In a further control (see Supporting Information), we prepared a synthetic NGF (122–241) lacking the chaperone peptide by KAHA ligation of **2–3–4** and a short α-ketoacid and confirmed that it did not fold under these or related conditions.

**Figure 4:**
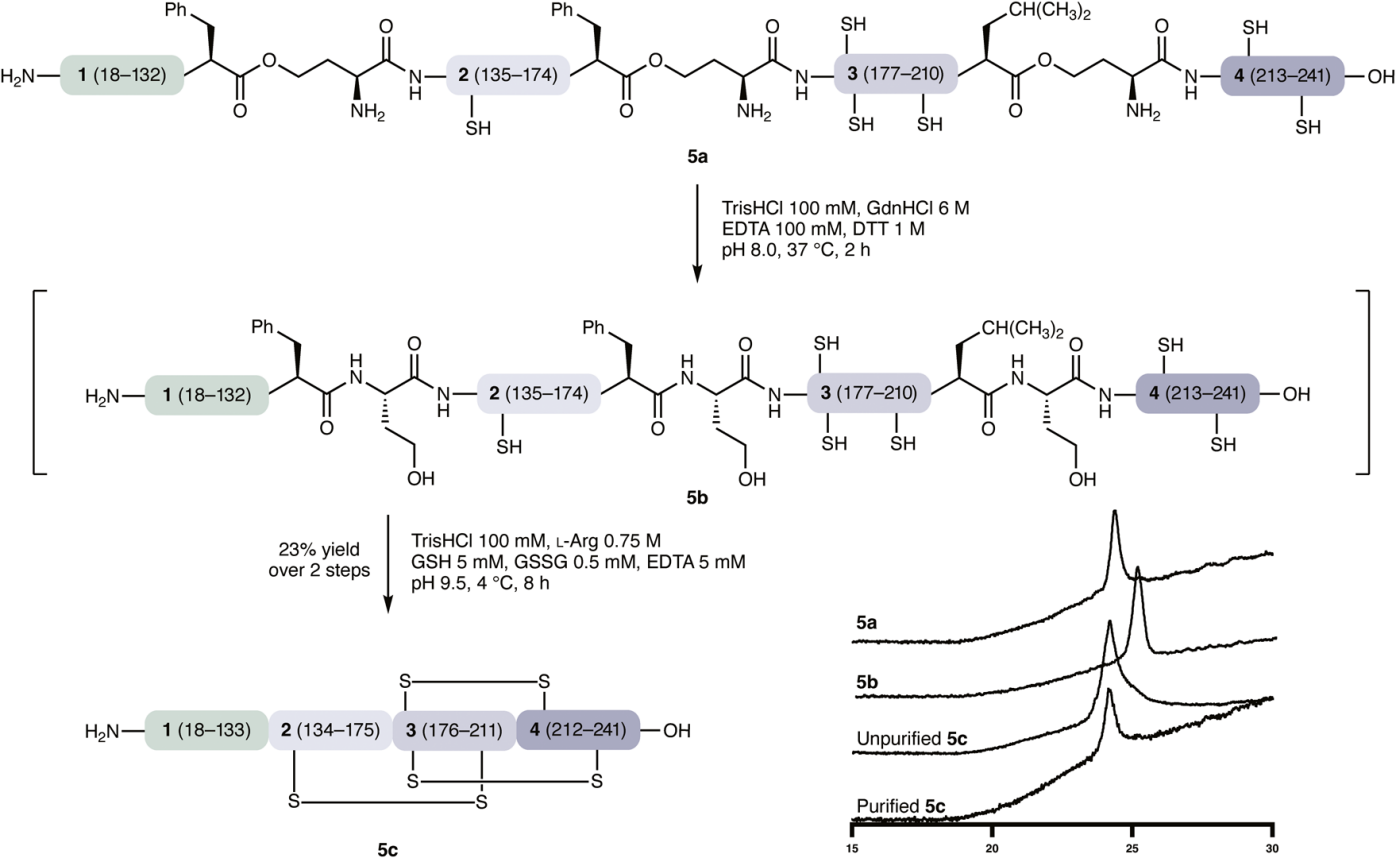
| O–>N shift and folding of semisynthetic proNGF. Conversion of *depsi* **5a** to *amide* **5b** and chaperone-mediated formation of three disulfide bonds.

### Enzymatic Cleavage of proNGF to NGF

The last remaining step in the synthesis of NGF was enzymatic processing of folded proNGF to remove the chaperone peptide, a key step in the maturation of proNGF to afford the 13 kDa βNGF (Figure 5a). We tested two previously reported methods. In the first, furin, a basic amino acid-cleaving enzyme, was used to selectively cleave at the dibasic processing site (KR/SS).^13^ The second uses limited digestion with trypsin, a serine protease which hydrolyses backbone amides after lysine or arginine residues.^31^ Using commercial, recombinant (CHO expressed) βNGF as a positive control, we observed conversion to the desired product by SDS-PAGE using both methods (see Supporting Information). We selected trypsin for processing of the semisynthetic material, as it both cleaves and digests the chaperone peptide.

**Figure 5:**
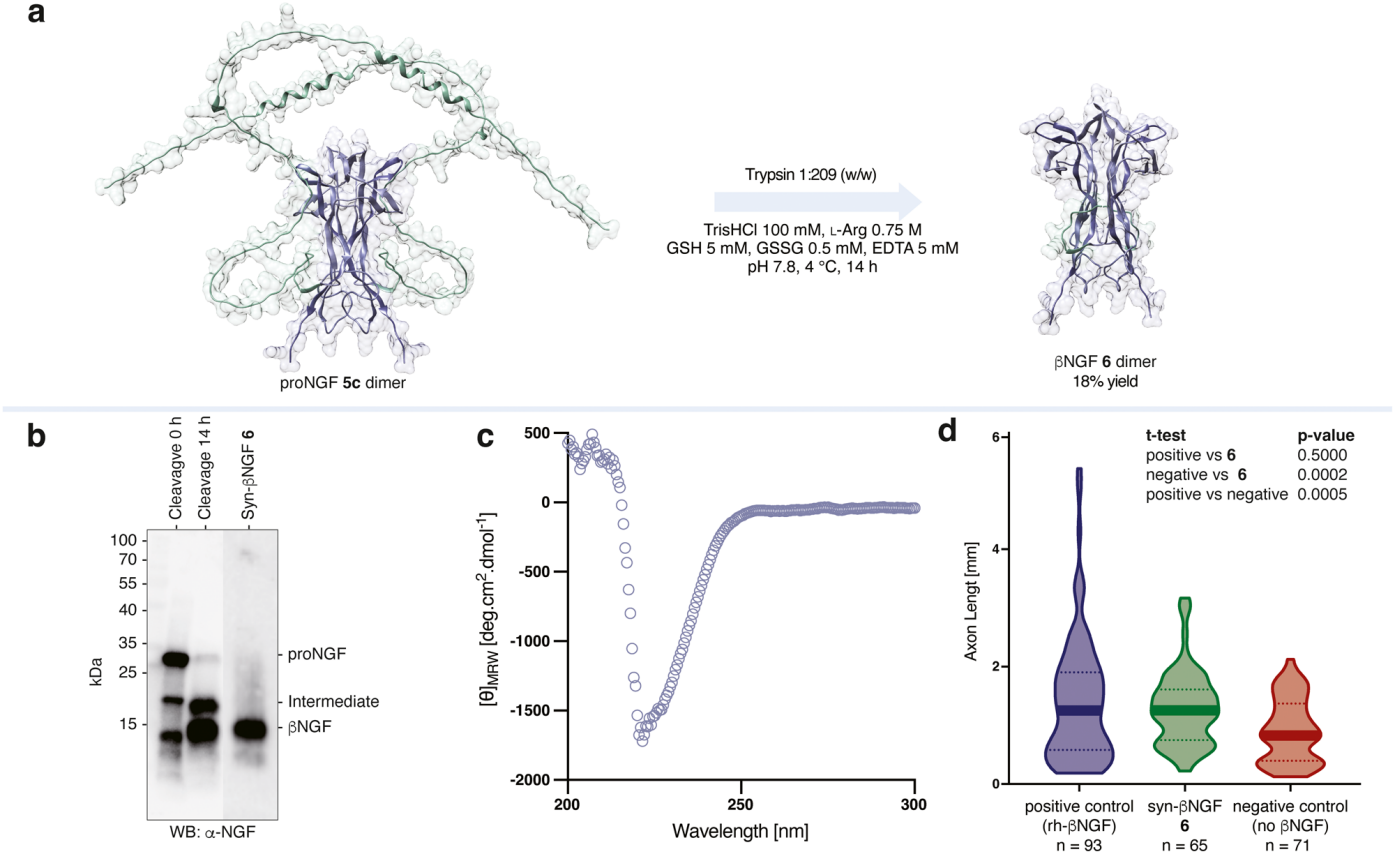
| Chaperone cleavage and biological activity. a, Enzymatic chaperone cleavage to afford mature βNGF dimer. The structures in this panel were generated with AlphaFold 3.^12^ b, Western blot of chaperone peptide cleavage with trypsin. c, CD spectrum of synthetic βNGF 6 in 10 mM KPhos buffer at pH 5.0. d, Violin plot depicting axon growth in neurons treated with recombinant, synthetic, and without βNGF.

Semisynthetic proNGF **5c** was converted to βNGF **6** by incubation with trypsin on ice for 14 h, monitored by RP-HPLC, followed by isolation by SEC. Consistent with previous reports, the chaperone peptide cleavage occurred via a metastable intermediate that could be observed by SDS-page and Western blot (Figure 5b).^31^ The RP-HPLC retention time of **6** was in close agreement with recombinant βNGF (see Supporting Information). Likewise, CD indicated the synthetic material exhibited the correct fold of antiparallel β-sheets (Figure 5c). As with proNGF, obtaining an intact mass spectrum of βNGF proved challenging but Western blot analysis of the isolated βNGF indicated a molecular weight of ∼ 14 kDa, in good agreement with the expected 13 kDa.

### Axon Growth Assay of Synthetic NGF

Recombinant and synthetic βNGF were further compared *in vitro* by analyzing their effects on axon growth in human induced pluripotent stem cell (iPSC)-derived dorsal root ganglion (DRG) neurons. These comparisons were conducted using polydimethylsiloxane (PDMS) microstructures, which provided a controlled environment for assessing neurite outgrowth by optical imaging.^32,33^ Both recombinant and synthetic βNGF promoted axon growth with similar lengths, indicating that the mutations introduced by the synthesis do not impair the desired biological activity of NGF (Figure 5d).

## Conclusion

We have established a chemical synthesis of biologically active human nerve growth factor (shNGF) by ester-forming KAHA ligations of three synthetic peptides and one recombinantly derived segment. Key to preparing and handling the synthetic, linear βNGF (122–241) was the introduction of a new semipermanent, Acm-derived solubility tag, SOLACE (Solubility Acm Enhancer). The Fmoc-Cys­(SOLACE)-OH amino acid could be introduced in Fmoc-SPPS using standard procedures and was stable to SPPS, resin cleavage, ligation, and Fmoc deprotection, but was removed in parallel with Acm. Furthermore, the 104-residue chaperone peptide, which is required for proper folding and disulfide-bond formation, was produced as an α-ketoacid from a recombinant source by site-specific transformation of an intein fusion to a cyanopyridinium ylide. This protocol offers a promising new method for the semisynthesis of proteins by combining materials derived from both synthetic and recombinant sources and assembled under KAHA ligation conditions well suited for handling and solubilizing exceptionally hydrophobic targets.

The unique challenges of NGF render it a difficult synthetic target, but the advances presented here – particularly the generation of the chaperone peptide by a combination of recombinant expression and site-specific introduction of the α-ketoacid – will provide a platform for the development of next generation βNGF-based therapeutics. The demonstration that KAHA ligation of fully synthetic segments can be combined with recombinant expression of the essential, but ultimately discarded, chaperone peptide paves the way for semisynthetic approaches to other neurotrophins with similar structure and function including brain-derived neurotrophic factor (BDNF, 119 residues +110 residue chaperone peptide), bone morphogenic protein (BMP, 114 residues +259 residue chaperone peptide), and transforming growth factor β-1 (112 residues +249 residue chaperone peptide).

## Supplementary Material





## Notes

General directions, experimental procedures, characterization data, NMR spectra, HPLC traces, and mass spectra (PDF)
Transparent Peer Review report available (PDF)
