# Supplementary material for: Chemical Synthesis and Chaperone Peptide Mediated Folding of Human Nerve Growth Factor by Expressed KAHA Ligation
Source: ACS Cent Sci. 2025 May 1;11(8):1321–8. doi: 10.1021/acscentsci.5c00277 (PMC12395302; doi:10.1021/acscentsci.5c00277)

Supporting Information

Chemical Synthesis and Chaperone Peptide Mediated Folding of Human Nerve Growth Factor by Expressed KAHA Ligation

Nicolas Y. Nötel[1†], Angus E. McMillan[1†], Vijaya R. Pattabiraman[1],
Katarina Vulić[2], and Jeffrey W. Bode[1*]

[1] Laboratory of Organic Chemistry, Department of Chemistry and Applied Biosciences, ETH Zürich, 8093 Zürich, Switzerland.

[2] Laboratory of Biosensors and Bioelectronics, ETH Zürich, 8092 Zürich, Switzerland.

*email: bode@org.chem.ethz.ch

† These authors contributed equally to this work.

Table of Contents

1 General Methods **2**

2 Amino Acid Sequence of proNGF **6**

3 Synthesis of 1-(Cyanomethyl)pyridin-1-ium Bromide **7**

4 Synthesis of Fmoc-Cys(SOLACE)-OH **8**

5 Expression and C-Terminal Modification **12**

6 SPPS **19**

7 Ligations and Deprotections **24**

8 Foldings **30**

9 Enzymatic Processing **33**

10 *In vitro* Axon Growth Assay **35**

11 Circular Dichroism Spectra **40**

12 Protein Sequences **41**

13 NMR Spectra **42**

Experimental Procedures

1 General Methods

Organic Synthesis

Unless otherwise noted, reactions were performed under an N_2 atmosphere. Reagents were used as received from commercial suppliers unless described otherwise. Reactions were monitored by thin layer chromatography (TLC) on Merck precoated aluminum-backed silica gel 60 F254 plates and visualized with UV at 254 nm or stained with a $KMnO_4$ solution. Flash chromatography purification was performed on Silicycle Silica Flash F60 (230–400 Mesh) silica gel using a forced flow of eluent at 0.4 bar.

NMR Characterization

NMR spectra were recorded on *Bruker* Avance III at 400 or 500 MHz for ^{1}H NMR and 100 or 125 MHz for ^{13}C NMR. Chemical shifts (δ) are reported in ppm, using the solvent as reference. NMR coupling constants (*J*) are reported in Hertz (Hz). All ^{13}C NMR spectra were measured with proton decoupling.

Mass Spectrometric Characterization

High-resolution mass spectra were recorded by the Molecular and Biomolecular Analysis Service (MoBiAS) at ETH Zürich with a Bruker maXis instrument (ESI-MS measurements) equipped with an ESI source and a QTOF detector. Ligation and protein modification products were analyzed using an ultra-high performance liquid chromatography (Acquity) coupled to a QTOF-MS instrument (Bruker, Compact Q-TOF MS, or Waters, G2 XS Q-TOF). Tandem MS experiments were performed using ESI-TIMS-QTOF-MS system (TimsTOF Pro, Bruker Daltonics, Germany) with collision-induced dissociation (CID) and N_2 as the collision gas.

IR Characterization

IR Spectra were recorded on a Jasco FT/IR-4100 spectrometer as thin films. Only major peaks are reported as frequency of absorption (cm^{-1}).

Melting Point Apparatus

Melting points were determined using a Buchi M-560 apparatus with a temperature gradient of 2.0 °C/min.

Optical Rotation

Optical rotations were measured on a Jasco P-2000 operating at the sodium D line with a 100 mm path length cell. Data reported as $[\alpha]_D$ (concentration (g/100 mL), solvent).

Solid Phase Peptide Synthesis

Peptides were synthetized from Fmoc-amino acids with suitable sidechain protecting groups. HCTU and HATU were purchased from Peptides International (Louisville, KY, USA) and ChemImpex (Wood Dale, IL, USA). Solid phase peptide synthesis (SPPS) on scales greater than 1 mmol were performed on a CS Bio 136X synthesizer. SPPS on scales less than 1 mmol were performed on a Gyros Protein Technologies Symphony X. Manual loading of the first amino acid residue onto the resin and subsequent Fmoc-SPPS followed established protocols. In brief, Fmoc-deprotections were performed with 20% piperidine in DMF (2 x 8 min). Couplings were performed with Fmoc-amino acid (4.0 equiv relative to resin substitution), HCTU (3.9 equiv), and NMM (8.0 equiv) in DMF for 60 min. If required, the coupling step was repeated once (double coupling). After coupling, unreacted free amine was capped by treatment with 20% acetic anhydride and 10% NMM in DMF (2 x 5 min). Amino acid residues prone to epimerization such as cysteine were coupled using preformed HOBt esters. In a typical procedure, Fmoc-Cys(Acm)-OH (5.0 equiv relative to resin loading) was dissolved in DMF, and HOBt (5.0 equiv) and DIC (5.0 equiv) were added. The mixture was added to the resin and allowed to react for 2 h. Valuable non-standard amino acids (Boc-Opr-OH, Fmoc-Opr-OH, and Fmoc-Cys(SOLACE)-OH) were coupled manually. The amino acid (1 equiv relative to resin substitution) was dissolved in a minimal amount of DMF, HATU (0.9 equiv), and DIPEA (2 equiv) were added. After a brief period of preactivation (3 min), the solution was added to the resin and allowed to react for 2 h.

a) Standard Amino Acids

The following standard Fmoc-amino acids were used: Fmoc-Ala-OH (A), Fmoc-Arg(Pbf)-OH (R), Fmoc-Asn(Trt)-OH (N), Fmoc-Asp(OtBu)-OH (D), Fmoc-Cys(Acm)-OH (C), Fmoc-Gln(Trt)-OH (Q), Fmoc-Gly-OH (G), Fmoc-Ile-OH (I), Fmoc-Leu-OH (L), Fmoc-Lys(Boc)-OH (K), Fmoc-Nle-OH (B), Fmoc-Phe-OH (F), Fmoc-Pro-OH (P), Fmoc-Ser(tBu)-OH (S), Fmoc-Thr(tBu)-OH (T), Fmoc-Trp(Boc)-OH (W), Fmoc-Tyr(tBu)-OH (Y), Fmoc-Val-OH (V).

b) Non-Standard Amino Acids

Boc-Opr-OH, Fmoc-Opr-OH, Fmoc-Leu-α-ketoacid, and Fmoc-Phe-α-ketoacid were prepared according to reported procedures.[1,2,3]

---

(1) Murar, C. E.; Harmond, T. J.; Bode, J. W. Improved synthesis of (S)-N-Boc-5-oxaproline for protein synthesis with the α-ketoacid-hydroxylamine (KAHA) ligation. *Bioorg. Med. Chem.* **2017**, *25*, 4996–5001.

(2) Murar, C. E.; Harmond, T. J.; Bode, J. W. Preparation of (S)-N-Boc-5-oxaproline. *Org. Synth.* **2018**, *95*, 157–186.

(3) Wucherpfennig, T. G.; Pattabiraman V. R.; Limberg F. R.; Ruiz-Rodríguez J.; Bode J. W. Traceless Preparation of C-Terminal α-Ketoacids for Chemical Protein Synthesis by α-Ketoacid-Hydroxylamine Ligation: Synthesis of Sumo2/3. *Angew.Chem. Int. Ed.* **2014**, *53*, 12248–12252.

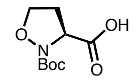

Boc-Oxaproline-OH

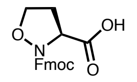

Fmoc-Oxaproline-OH

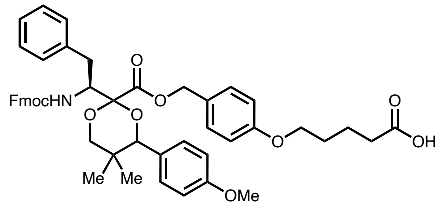

Fmoc-Phe-α-ketoacid-OMe-linker

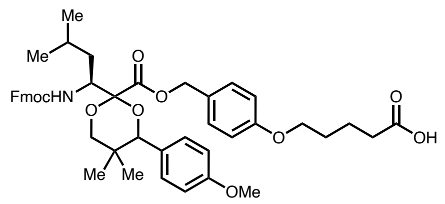

Fmoc-Leu-α-ketoacid-OMe-linker

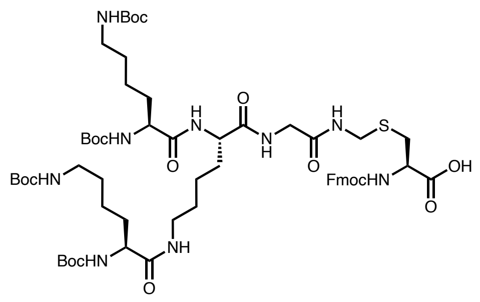

Fmoc-Cys(SOLACE)-OH

Resin Cleavage Protocol

The dry resin was placed in a glass vial, a mixture of TFA/DODT/H_2O (95:2.5:2.5, 15 mL/g of resin) was added, and the suspension shaken for 1.5 h. The resin was removed by filtration and washed with TFA (5 mL/g resin), the filtrate was placed in a plastic falcon tube (40 mL), and volatiles removed under reduced pressure. The residue was triturated with Et_2O (ca. 15 mL/g resin), centrifuged (2500 x g, 4 min), and the supernatant was removed by decantation. The crude material was dried and dissolved in a suitable solvent (CH_3CN/H_2O 1:1 + 0.1% TFA) for RP-HPLC purification.

Reverse Phase High Performance Liquid Chromatography

Reverse phase high performance liquid chromatography (RP-HPLC) was carried out using Jasco analytical and preparative instruments with dual pumps, mixer, and in-line degasser. Monitoring was achieved using a variable wavelength UV Rheodyne detector (recording 220, 254, and 301 nm). The instrument was equipped with a 7725i injector. For further details on the HPLC methods used including columns and gradients, see the RP-HPLC methods table below.

Method	Column	Conditions		
Analytical A	Shiseido	Temp: 60 °C	Inject. Loop: 1000 µL	Flow Rate: 1 mL/min
	Capcell Pak	Time (min)	% CH_3CN (+0.1% TFA)	% H_2O (+0.1% TFA)
	C18 UG120	0	20	80
	250 x 4.6	3	20	80
	AOAD1320	17	95	5
		22	95	5
		22.1	20	80
		25	20	80
Analytical B	Shiseido	Temp: 60 °C	Inject. Loop: 50 µL	Flow Rate: 1 mL/min
	Capcell Pak	Time (min)	% CH_3CN (+0.1% TFA)	% H_2O (+0.1% TFA)
	C18 UG120	0	20	80
	250 x 4.6	3	20	80
	AOAD1320	17	95	5
		22.1	20	80
		25	20	80
Analytical Folding A	Grace Vydac	Temp: rt	Inject. Loop: 1000 µL	Flow Rate: 1 mL/min
	214TP54 C4	Time (min)	% CH_3CN (+0.1% TFA)	% H_2O (+0.1% TFA)
	250 x 4.6	0	5	95
	E041129-4-1	10	5	95
		15	40	60
		30	70	30
		31	95	5
		33	95	5
		33.1	80	20
		38	20	20
Analytical Folding B	Shiseido	Temp: rt	Inject. Loop: 1000 µL	Flow Rate: 1 mL/min
	Capcell Pak	Time (min)	% CH_3CN (+0.1% TFA)	% H_2O (+0.1% TFA)
	C18 MGII	0	5	95
	250 x 4.6	15	5	95
	AKAD03091	23	20	80
		33	20	80
		47	95	5
		52	95	5
		60	5	95
Prep A	Shiseido	Temp: 60 °C	Inject. Loop: 20 mL	Flow Rate: 10 mL/min
	Capcell Pak	Time (min)	% CH_3CN (+0.1% TFA)	% H_2O (+0.1% TFA)
	C18 MGII	0	20	80
	250 x 20	5	20	80
	A4ED01107	35	95	5
		40	95	5
		40.1	20	80
		44.5	20	80
Prep B	Shiseido	Temp: rt	Inject. Loop: 5 mL	Flow Rate: 10 mL/min
	Capcell Pak	Time (min)	% CH_3CN (+0.1% TFA)	% H_2O (+0.1% TFA)
	C18 MGII	0	5	95
	250 x 20	5	5	95
	A4ED01107	35	95	5
		40	95	5
		40.1	5	95
		44.5	5	95
Semi-Prep Folding	Shiseido	Temp: rt °C	Inject. Loop: 20 mL	Flow Rate: 10 mL/min
	Proteonavi C4	Time (min)	% CH_3CN (+0.1% TFA)	% H_2O (+0.1% TFA)
	250 x 10	0	5	95
	J20CD01041	10	5	95
		15	30	70
		35	60	40
		36	95	5
		40	95	5
		40.1	5	95
		44.8	5	95

Size Exclusion Chromatography

Size exclusion chromatography (SEC) was carried out on a Superdex 75 Increase 10/300 GL column and a Jasco analytical instrument. Monitoring was achieved using a fluorescence detector (exciting

280 nm, recording 310 nm). The instrument was equipped with a 7725i injector and a 1 mL injection loop. Samples were eluted with degassed 50 mM sodium acetate buffer at pH 5.5.

2 Amino Acid Sequence of proNGF

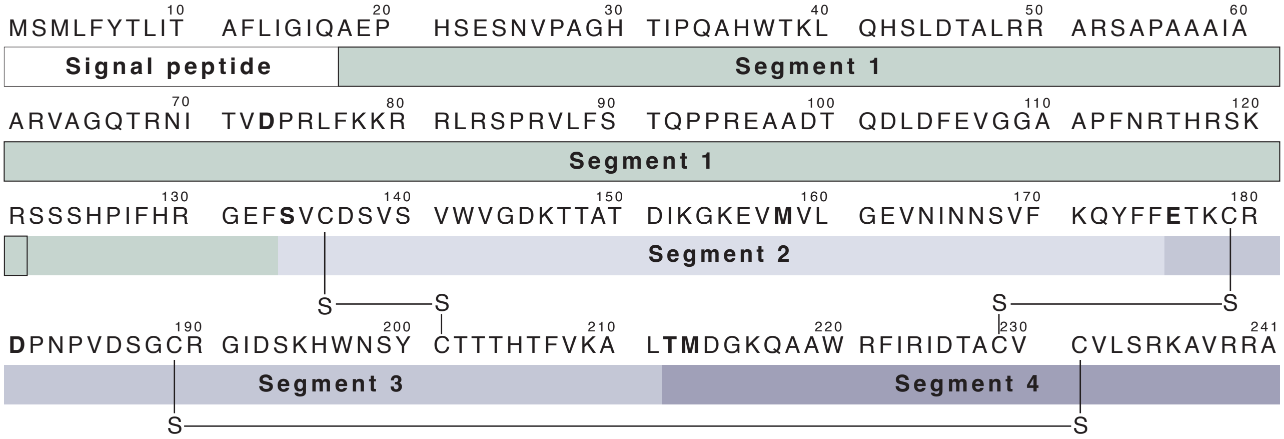

Mutations: D73E, S134Hse, M158Nle, E176Hse, D181E, T212Hse, M213Nle

a) Numbering: the amino acid numbering corresponds to UniProt P01138, with the signal peptide excluded from the synthesis.

b) Ligation sites: Phe133-Ser134, Phe175-Glu176, and Leu211-Thr212 leading to three homoserine (Hse) mutations at the KAHA ligation sites, which are S134Hse, E176Hse, and T212Hse.

c) Norleucine substitutions: two methionine residues were substituted by norleucine residues (M158Nle, M213Nle) to avoid oxidation while handling, storage, and refolding.

d) Asp-to-Glu substitutions: two aspartate residues were substituted by glutamate residues (D73E, D181E) to mitigate cleavage of the Asp-Pro bonds under acidic aqueous conditions.

e) Chaperone peptide: the chaperone peptide is outlined and was removed after folding by enzymatic processing.

3 Synthesis of 1-(Cyanomethyl)pyridin-1-ium Bromide

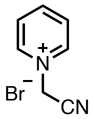

Pyridine (20 g, 0.25 mol, 1.0 equiv) was dissolved in anhydrous THF (125 mL). Bromoacetonitrile (17 mL, 0.25 mol, 1.0 equiv) was added and the reaction mixture was stirred at 50 °C for 44 h. A white precipitate formed which was collected by filtration, washed with Et_2O, and dried under reduced pressure to afford the title compound as a white powder (36.1 g, 0.18 mol, 73% yield).

^{1}H NMR (500 MHz, DMSO-*d*$_6$) δ 9.35 (dd, *J* = 6.7, 1.4 Hz, 2H), 8.79 (tt, *J* = 7.8, 1.4 Hz, 1H), 8.32 (t, *J* = 7.8 Hz, 1H), 6.33 (d, *J* = 1.2 Hz, 2H).

^{13}C NMR (126 MHz, DMSO-*d*$_6$) δ 147.7, 145.3 (2C), 128.6 (2C), 114.2, 47.6.

Characterization agreed with reported data.[4]

(4) Day, J.; Uroos, M.; Castledine, R. A.; Lewis, W.; McKeever-Abbas, B.; Dowden, J. Alkaloid inspired spirocyclic oxindoles from 1,3-dipolar cycloaddition of pyridinium ylide. *Org. Biomol. Chem.* **2013**, *11*, 6502–6509.

4 Synthesis of Fmoc-Cys(SOLACE)-OH

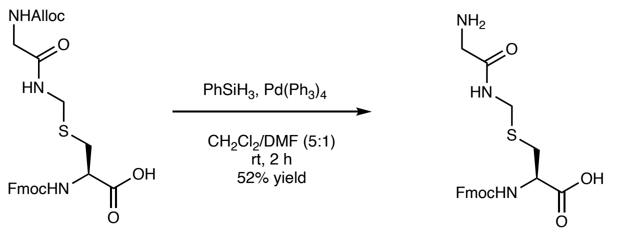

S1 **S2**

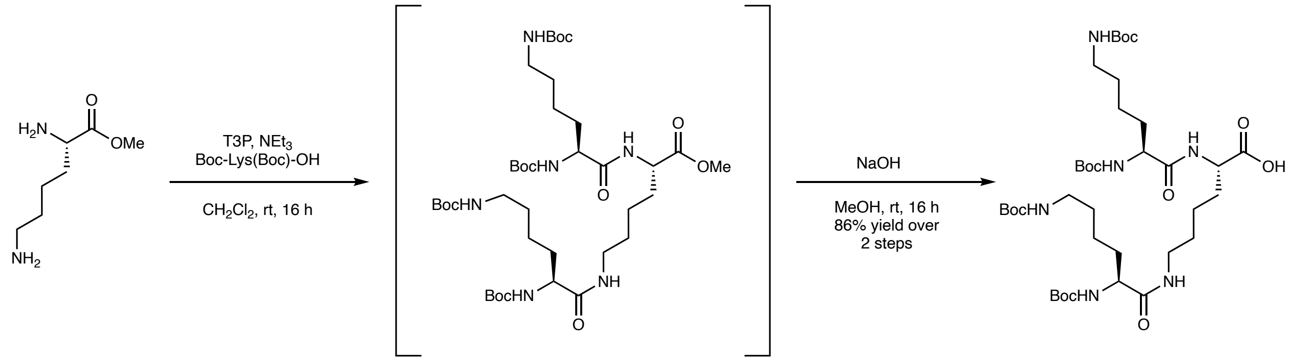

S3 **S4a** **S4b**

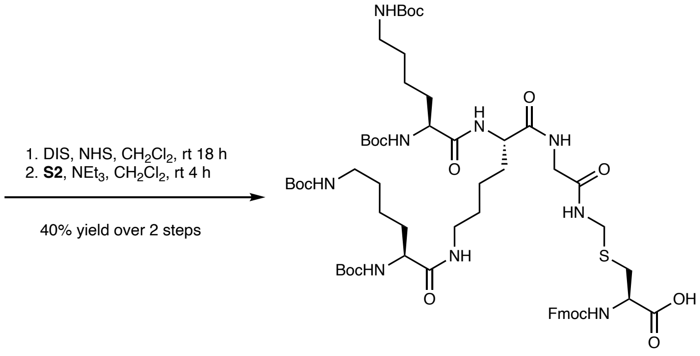

S5

N-(((9H-fluoren-9-yl)methoxy)carbonyl)-S-((2-aminoacetamido)methyl)-L-cysteine (S2)

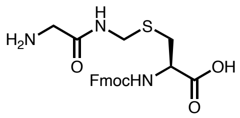

Compound **S1**[5] (0.49 g, 1.0 mmol, 1.0 equiv) was dissolved in degassed CH_2Cl_2/DMF (5:1, 10 mL). Tetrakis-(triphenylphosphin)-palladium (26 mg, 23 μmol, 0.02 equiv) and phenyl silane (270 mg, 2.5 mmol, 2.5 equiv) were added and the reaction was stirred under an N_2 atmosphere at rt for 2 h. The reaction mixture was concentrated and purified by flash silica chromatography (30–40% MeOH in DCM + 1% formic acid). The fractions containing product were combined, concentrated, and azeotropically dried three times with toluene to remove formic acid, affording **S2** as an off white amorphous solid (220 mg, 0.51 mmol, 52% yield).

^{1}H NMR (500 MHz, DMSO-d_6) δ 9.0 (t, J = 5.0 Hz, 1H), 8.3 (s, 1H), 7.9 (dt, J = 7.6, 1.0 Hz, 2H), 7.7 (dd, J = 7.5, 4.7 Hz, 2H), 7.4 (td, J = 7.5, 1.1 Hz, 2H), 7.3 (tdd, J = 7.4, 2.6, 1.2 Hz, 2H), 7.0 (d, J = 7.6 Hz, 1H), 4.9 (br s, 3H), 4.4 – 4.1 (m, 6H), 4.0 (td, J = 7.5, 4.6 Hz, 1H), 3.1 (dd, J = 13.7, 4.3 Hz, 1H), 2.9 (dd, J = 13.7, 7.4 Hz, 1H).

^{13}C NMR (126 MHz, DMSO-d_6, signals from minor Fmoc rotamer*) δ 172.9, 167.6, 164.4, 155.5, 143.9, 143.8, 140.7, 128.9*, 127.6 (2C), 127.3*, 127.1 (2C), 125.3, 125.3, 121.4*, 120.1 (2C), 65.6, 55.6, 46.7, 41.4, 40.8, 33.8.

HR-MS (ESI) found 430.1430, calculated for $C_{21}H_{23}N_3O_5S$ $[M+H]^+$; 430.1431.

m.p. 102 – 105 °C.

IR (thin film) 3347, 1693, 1529, 1254, 1043, 736 cm^{-1}.

$[\alpha]_D^{23}$ = –15.9° (c 0.35, DMSO).

Methyl *N*^{2},*N*^{6}-bis(*N*^{2},*N*^{6}-bis(*tert*-butoxycarbonyl)-*L*-lysyl)-*L*-lysinate (S4a)

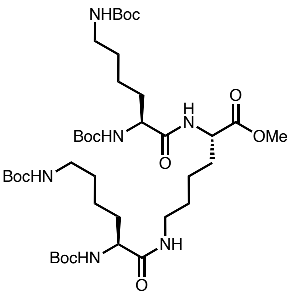

Boc-Lys(Boc)-OH (4.36 g, 12.6 mmol, 2.2 equiv) was dissolved in anhydrous CH_2Cl_2 (15 mL). Triethylamine (5.01 mL, 36 mmol, 6.3 equiv) and 2,4,6-tripropyl-1,3,5,2,4,6-trioxatriphosphinane 2,4,6-trioxide (8.5 mL, 12.5 mmol, 2.2 equiv, 50% in EtOAc) were added. The reaction mixture was

(5) Brailsford, J. A.; Stockdill, J. L; Axelrod, A. J.; Peterson, M. T.; Vadola, P. A.; Johnston, E. V.; Danishefsky, S. J. Total chemical synthesis of human thyroid-stimulating hormone (hTSH) *β*-subunit: Application of arginine-tagged acetamidomethyl (Acm^R) protecting groups. *Tetrahedron*, 2018, **74**, 1951–1956.

stirred for 5 min and compound **S2** (1.40 g, 5.67 mmol, 1.0 equiv) was added in CH_2Cl_2 (15 mL). The reaction mixture was stirred at rt for 16 h. The reaction mixture was diluted with CH_2Cl_2 (100 mL), washed with water (3 x 20 mL) and a saturated aq. solution $NaHCO_3$ (2 x 20 mL). The solution was dried over anhydrous sodium sulphate, concentrated under reduced pressure, and purified by flash silica chromatography (40–80% EtOAc in *n*-hexane) to afford compound **S4a** as a white foam (4.23 g, 5.18 mmol, 91% yield).

^{1}H NMR (500 MHz, $CDCl_3$) δ 7.5 – 7.3 (m, 1H), 7.0 – 6.9 (m, 1H), 6.0 – 5.8 (m, 1H), 5.6 – 5.5 (m, 1H), 5.0 – 4.8 (m, 1H), 4.8 – 4.7 (m, 1H), 4.4 – 4.3 (m, 2H), 4.2 – 4.1 (m, 1H), 3.7 (s, 3H), 3.6 – 3.5 (m, 1H), 3.2 – 3.0 (m, 4H), 3.0 (m, 1H), 1.9 – 1.8 (m, 3H), 1.7 – 1.6 (m, 3H), 1.6 – 1.2 (m, 48H).

^{13}C NMR (126 MHz, $CDCl_3$) δ 173.7, 173.3, 172.7, 156.6, 156.3, 156.2, 156.2, 80.1, 80.0, 79.1, 79.0, 54.6, 53.9, 52.4, 52.1, 40.3 (2C), 37.8, 32.7, 31.7, 30.8, 29.8, 29.5, 28.6 (6C), 28.6 (3C), 28.5 (3C), 23.1, 22.7 (2C), 22.0.

HR-MS (ESI) found 817.5277, calculated for $C_{39}H_{72}N_6O_{12}$ $[M+H]^+$: 817.5281.

IR (thin film) 3297, 26976, 2932, 2865, 1688, 1652, 1510, 1364, 1247, 1164 cm^{-1}.

$[\alpha]_D^{22}$ = –31.3° (c 1.9, $CDCl_3$).

m.p. 74 – 76 °C.

***N*^{2},*N*^{6}-bis(*N*^{2},*N*^{6}-bis(*tert*-butoxycarbonyl)-*L*-lysyl)-*L*-lysine (S4b)**

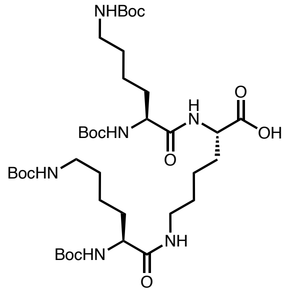

Compound **S4a** (2.24 g, 2.74 mmol, 1.00 equiv) was dissolved in MeOH (50 mL). NaOH (5.48 mL of a 1 M solution, 5.48 mmol, 2.00 equiv) was added and the reaction mixture was stirred at rt for 16 h. The reaction mixture was concentrated under reduced pressure, brine was added, and the pH was adjusted to 3.0 by addition of AcOH. The product was extracted in EtOAc (4 x 150 mL), dried over anhydrous sodium sulfate, and concentrated under reduced pressure to afford compound **S4b** as a white foam (2.09 g, 2.60 mmol, 95% yield).

^{1}H NMR (500 MHz, DMSO-*d*$_6$) δ 7.9 (d, *J* = 7.8 Hz, 1H), 7.7 (t, *J* = 5.6 Hz, 1H), 6.8 – 6.6 (m, 3H), 4.2 (td, *J* = 8.4, 5.0 Hz, 1H), 3.9 (td, *J* = 8.7, 4.8 Hz, 1H), 3.8 (td, *J* = 8.5, 4.9 Hz, 1H), 3.1 – 2.9 (m, 2H), 2.9 – 2.8 (m, 4H), 1.8 – 1.6 (m, 1H), 1.6 – 1.2 (m, 55H).

^{13}C NMR (126 MHz, DMSO-*d*$_6$) δ 174.0 (2C), 172.7, 172.4, 156.0 (2C), 155.7, 155.7, 78.4, 78.4, 77.8 (2C), 54.8, 54.6, 52.0, 40.0, 38.7, 32.3, 32.1, 31.2, 29.7, 29.7, 29.1, 28.7 (6C), 28.6 (5C), 28.5, 23.3, 23.2, 23.0.

HR-MS (ESI) found 803.5121, calculated for $C_{38}H_{70}N_6O_{12}$ $[M+H]^+$: 803.5124.

m.p. 185 – 187 °C.

IR (thin film) 3307, 2976, 1652, 1515, 1365, 1248, 1164 cm^{-1}.

[α]$_D^{27}$ = –6.6° (c 0.8, EtOH).

Fmoc-Cys(SOLACE)-OH (S5)

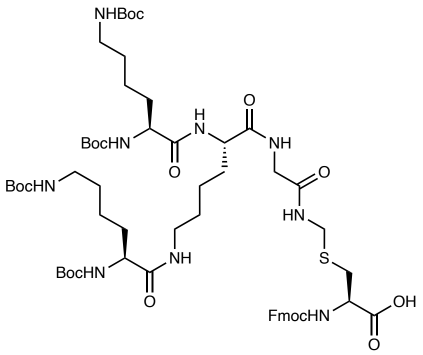

N,N′-diisopropylcarbodiimide (86 μL, 550 μmol, 1.1 equiv) and compound **S4b** (400 mg, 500 μmol, 1.0 equiv) were dissolved in CH_2Cl_2 (5 mL) and stirred at rt for 5 min. *N*-hydroxysuccinimide (63 mg, 550 μmol, 1.1 equiv) was added and the reaction mixture was stirred for a further 18 h at rt. Triethylamine (153 μL, 110 μmol, 2.2 equiv) and compound **S2** (215 mg, 500 μmol, 1.0 equiv) were added. The reaction mixture was stirred for a further 4 h at rt, concentrated under reduced pressure, and purified by flash silica chromatography (10–40% MeOH in EtOAc + 0.1% formic acid). The relevant fractions were combined, concentrated, and azeotropically dried three times with toluene to remove formic acid. The resulting colorless oil was dissolved in CH_3CN/H_2O (1:1) and lyophilized to obtain a white solid (240 mg, 200 μmol, 40% yield).

^{1}H NMR (500 MHz, DMSO-d6) δ 8.5 – 8.5 (m, 1H), 8.2 – 8.2 (m, 1H), 7.9 – 7.8 (m, 3H), 7.8 – 7.7 (m, 4H), 7.4 (td, J = 7.5, 1.1 Hz, 2H), 7.3 (td, J = 7.4, 1.2 Hz, 2H), 6.9 – 6.9 (m, 1H), 6.8 – 6.7 (m, 3H), 4.3 – 4.2 (m, 7H), 3.9 – 3.8 (m, 2H), 3.8 – 3.6 (m, 3H), 3.1 – 2.9 (m, 3H), 2.9 – 2.8 (m, 5H), 1.7 – 1.6 (m, 1H), 1.6 – 1.4 (m, 6H), 1.4 – 1.3 (m, 41H), 1.3 – 1.1 (m, 6H).

^{13}C NMR (126 MHz, DMSO-*d$_6$*) δ 172.8, 172.7, 172.4, 169.3, 158.9, 158.6, 156.5, 156.0 (2C), 155.9, 155.7, 144.3 (2C), 141.2 (2C), 128.1 (2C), 127.6 (2C), 125.8 (2C), 120.6 (2C), 78.6, 78.4 (4C), 77.8, 66.3, 54.8, 54.8, 54.4, 52.9, 47.1 (2C), 42.4, 40.9, 40.2, 38.8, 32.3, 32.0, 31.9, 29.7, 29.2, 28.7 (9C), 28.7 (3C), 23.3, 22.9.

HR-MS (ESI) found 1214.6373, calculated for $C_{59}H_{91}N_9O_{16}S$ $[M+H]^+$: 1214.6377.

IR (thin film) 32297, 2916, 1656, 1513, 1366, 1159, 1046, 857 cm^{-1}.

[α]$_D^{23}$ = –8.1° (c 0.8, DMSO-*d$_6$*).

5 Expression and C-Terminal Modification

Segment 1a

Chemically competent BL21 (DE3) cells were heat-shock transformed with the pET-29a-Segment **1**-GyrA-His_6 and a single colony was used to inoculate an overnight preculture in selective lysogeny broth (LB) Miller medium. See section *12 Protein Sequences* for fusion protein sequence. The following expression media (0.5 L / flask) was prepared in 10 x 2 L baffled expression flasks then autoclaved; Na_2HPO_4 6 g/L, KH_2PO_4 3 g/L, tryptone 20 g/L, yeast extract 5 g/L, NaCl 5 g/L, water (0.5 L/flask). Once cool the following were added: glycerol (60% v/v, 10 mL/L), glucose (10% w/v, 5 mL/L), lactose (8% w/v, 25 mL/L), and kanamycin (100 mg/L). The media was inoculated with overnight culture (1 mL/L) and incubated at 37 °C, 120 rpm until the O.D. reached 0.6 (typically 4–6 h). The temperature was adjusted to 30 °C for 20 h.

The cells were harvested at 22,000 x g then resuspended in lysis buffer (tris 50 mM, NaCl 200 mM, pH 7.2, 50 mL/L). DNase (20 μg/mL) and lysozyme (0.4 μg/mL) were added and the suspension was incubated at 4 °C for 0.5 h. On ice the suspension was sonicated (70% power, 2 x 1 min in 40 mL batches, Bandelin Sonopuls HD 2070 ultrasonic homogenizer). The suspension was centrifuged at 22,000 x g for 0.5 h then the supernatant was removed. The precipitate was resuspended in lysis buffer (tris 50 mM, NaCl 200 mM, pH 7.5, 50 mL/L) then centrifuged again at 22,000 x g for 0.5 h. The supernatant was removed, and the precipitate stored at –80 °C. The precipitate was resuspended in denaturing buffer (tris 50 mM, NaCl 200 mM, GdnHCl 6 M, DTT 100 mM, pH 7.2, 50 mL/L). The suspension was centrifuged at 22,000 x g for 0.5 h, the precipitate was discarded, and the supernatant was dialyzed twice in refolding buffer (tris 20 mM, NaCl 200 mM, pH 7.2, 1.25 L/L). A significant amount of precipitate was removed by centrifugation at 22,000 x g, 0.5 h then the supernatant was purified using Ni-NTA resin (2.5 mL/L) with elution in imidazole buffer (tris 50 mM, NaCl 200 mM, imidazole 300 mM, pH 7.2). The protein containing fractions were combined and diluted to 3.4 mg/mL then dialyzed three times against dialysis buffer (tris 20 mM, NaCl 200 mM, pH 7.2, 1.25 L/L), giving a yield of 36.0 mL/L at 3.4 mg/mL (122.4 mg/L).

To the solution of Segment **1**-GyrA-His_6 (150 mL, 3.4 mg/mL), MesNa (4.9 g, 200 mM), TCEP (43 mg, 1 mM), and EDTA (44 mg, 1 mM) were added. The pH was adjusted to 7.4 by addition of NaOH (2M). The solution was stirred at rt for 20 h, passed through a 0.2 μm filter then purified by RP-HPLC using Prep A method. The fractions containing segment **1a** were combined and lyophilized to give a final yield of 27 mg/L.

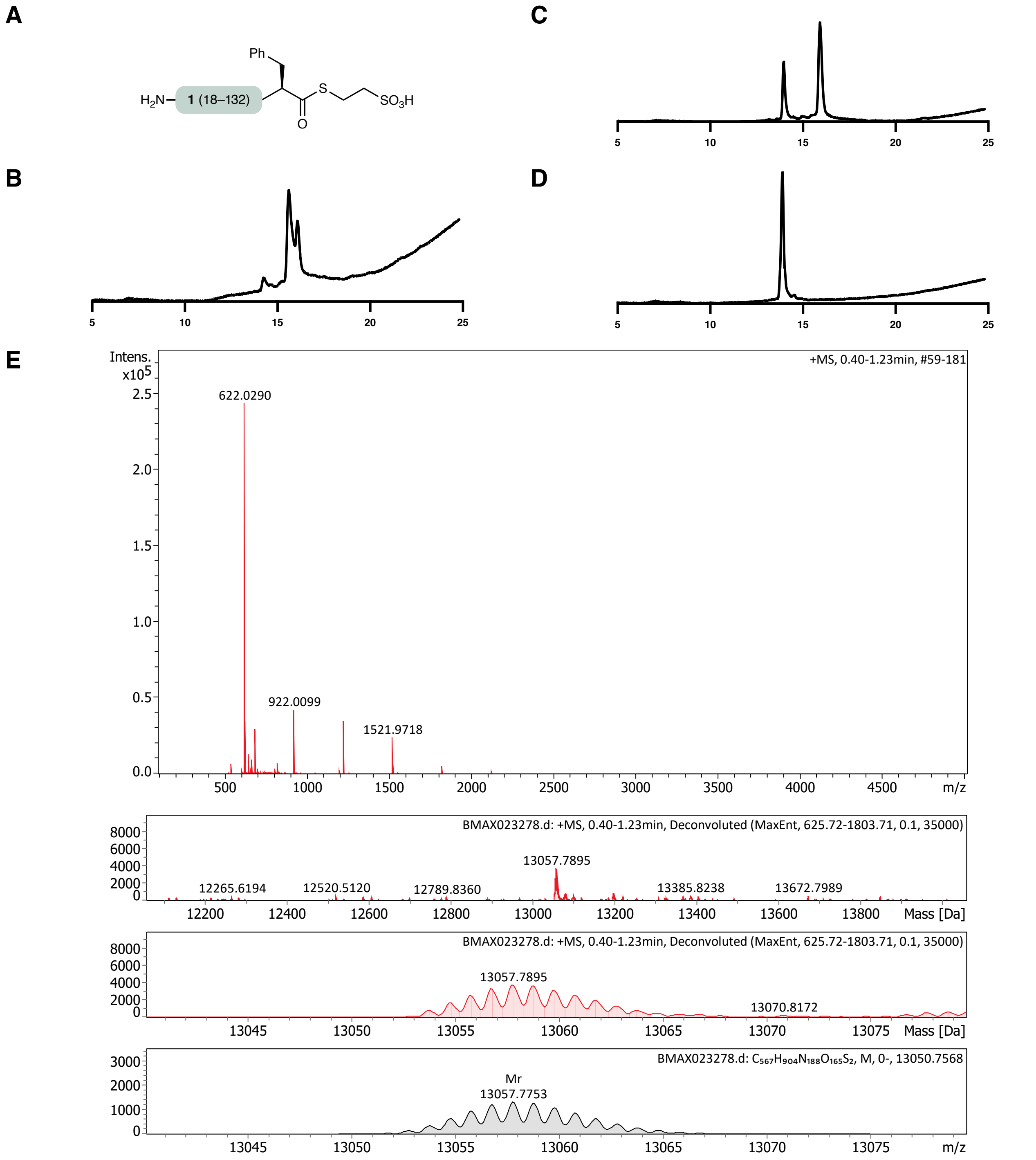

Figure S1. (A) Structure of segment **1a**. (B) HPLC chromatogram of crude thiolysis at time 0 h using analytical A method. (C) HPLC chromatogram of crude thiolysis at time 20 h using analytical A method. (D) HPLC chromatogram of purified product using analytical A method. (E) HR-MS(ESI), found: 13057.7895 (second and third panels), calculated for $C_{567}H_{904}N_{188}O_{165}S_2$ [M]: 13057.7753 (fourth panel).

Segment 1b

Segment **1a** (105 mg, 8.0 μmol) was dissolved in DMSO (806 μL) and diluted with buffer (KPhos 50 mM, NaCl 200 mM, 7.25 mL) to give a final concentration of 1 mM. 1-(Cyanomethyl)pyridin-1-ium bromide (4.01 g, 20.1 mmol, 2500 equiv) was added and the pH was adjusted to 8.2 by careful addition of aq. NaOH (2 M). The mixture was incubated at rt for 1.5 h. The product was diluted with H_2O (+0.1 % formic acid, 8.06 mL), purified by RP-HPLC using Prep B method, and lyophilized to afford segment **1b** (54.5 mg, 4.2 μmol, 55% yield). The persistent pyridinium cations (CyP, m/z 118) were optionally removed by dialyzing the sample at 200 μM in Milli-Q water, 45 mL x 3 over 24 h, in a Slide-A-Lyzer MINI Dialysis Device (3.5 K MWCO, 2 mL). The product was lyophilized and recovered in quantitative yield.

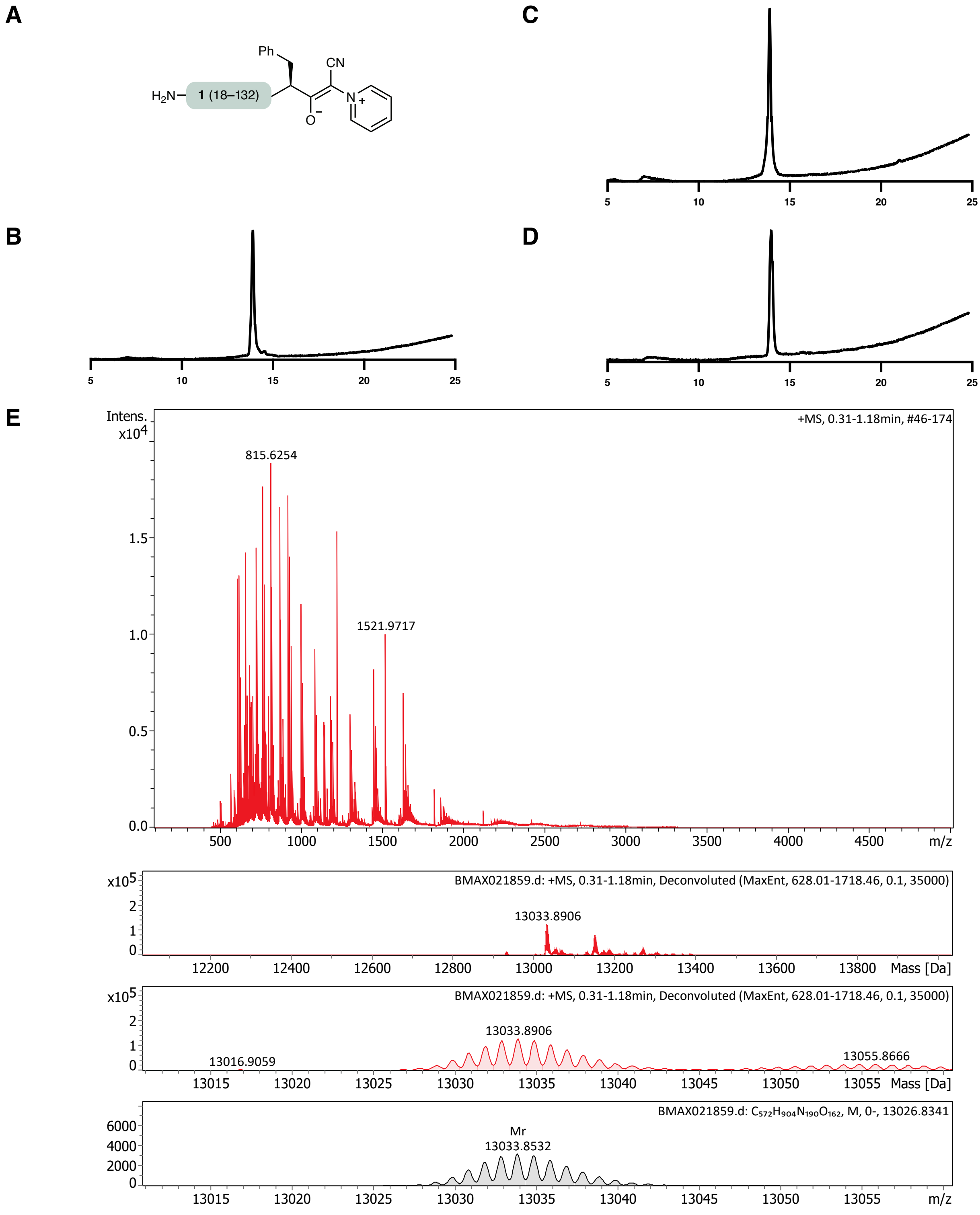
A
H2N– 1 (18–132)
Ph
CN
N+
O−
B
C
D
E
Intens.
x10^4
+MS, 0.31-1.18min, #46-174
815.6254
1521.9717
m/z
BMAX021859.d: +MS, 0.31-1.18min, Deconvoluted (MaxEnt, 628.01-1718.46, 0.1, 35000)
13033.8906
Mass [Da]
13016.9059
13055.8666
BMAX021859.d: C572H904N190O162, M, 0-, 13026.8341
Mr
13033.8532

F

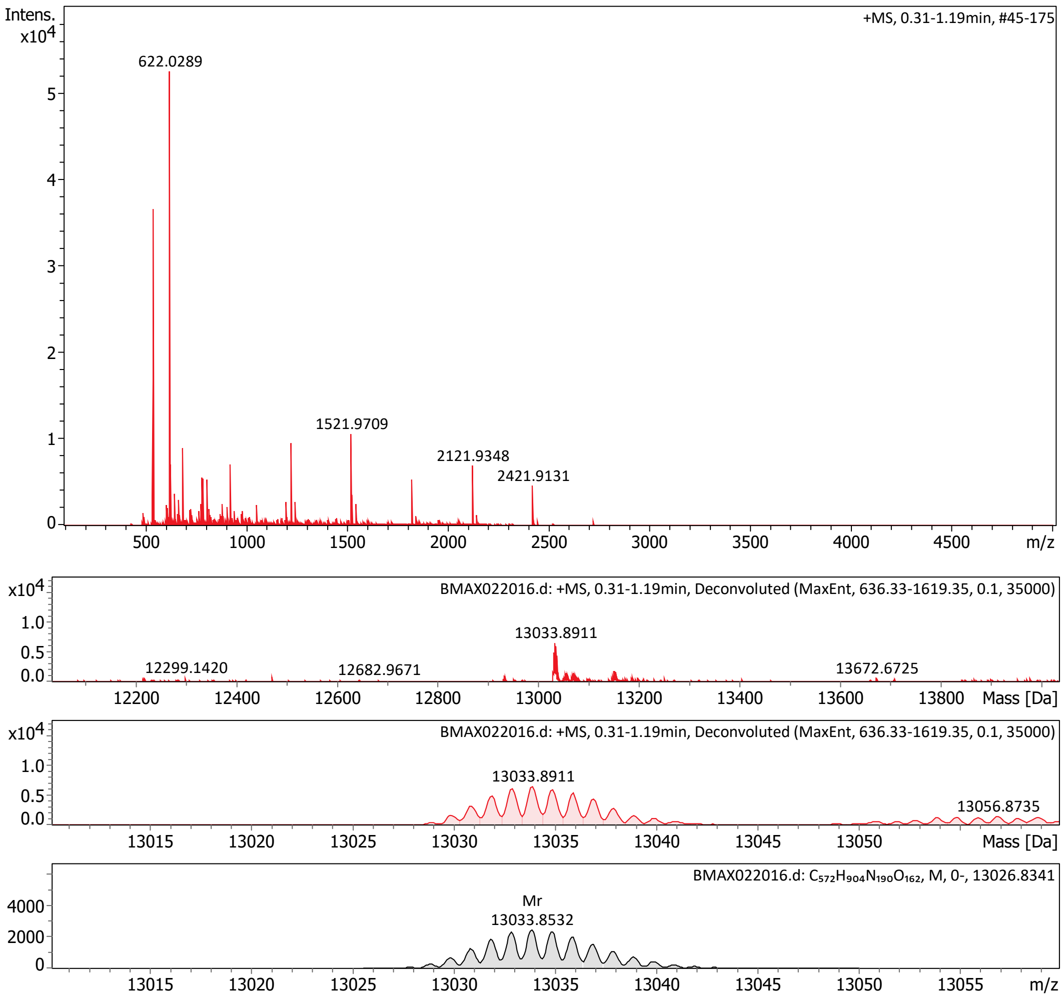

Figure S2. (A) Structure of segment **1b**. (B) HPLC chromtogram of **1b** using analytical A method. (C) HPLC chromatogram of the crude reaction mixture after 1.5 h using analytical A method. (D) HPLC chromatogram of the purified product using analytical A method. (E) HR-MS (ESI) before dialysis shows $[M–H+CyP]^+$ and $[M–2H+2CyP]^+$ ions (second panel). Found: 13033.8906 (second and third panels), calculated for $C_{572}H_{904}N_{190}O_{162}$ [M]: 13033.8532 (fourth panel). (F) HR-MS (ESI) after dialysis. Found: 13033.8911 (second and third panels), calculated for $C_{572}H_{904}N_{190}O_{162}$ [M]: 13033.8532 (fourth panel).

Segment 1c

Segment **1b** (69 mg, 5.3 μmol) was dissolved in CH_3CN/H_2O (1:1, 2.7 mL). A solution of tryptophan (40 mM stock solution in CH_3CN/H_2O 1:1 +0.1% TFA, 2.7 mL) and Oxone (20 mM stock solution in CH_3CN/H_2O 1:1 +0.1% TFA, 1.1 mL) were added to give final concentrations of segment **1b** (830 μM, 1.0 equiv), tryptophan (16.6 mM, 20 equiv), and Oxone (3.41 mM, 4.0 equiv). The reaction was mixed for 4 min, then quenched by addition of excess dimethyl sulfide (500 μL, 6.8 mmol, 1283 equiv). The excess dimethyl sulfide was removed by purging the reaction solution with N_2 and the product was purified by RP-HPLC using Prep A method. Lyophilization of the relevant fractions afforded segment **1c** (18 mg, 1.4 μmol, 26% yield).

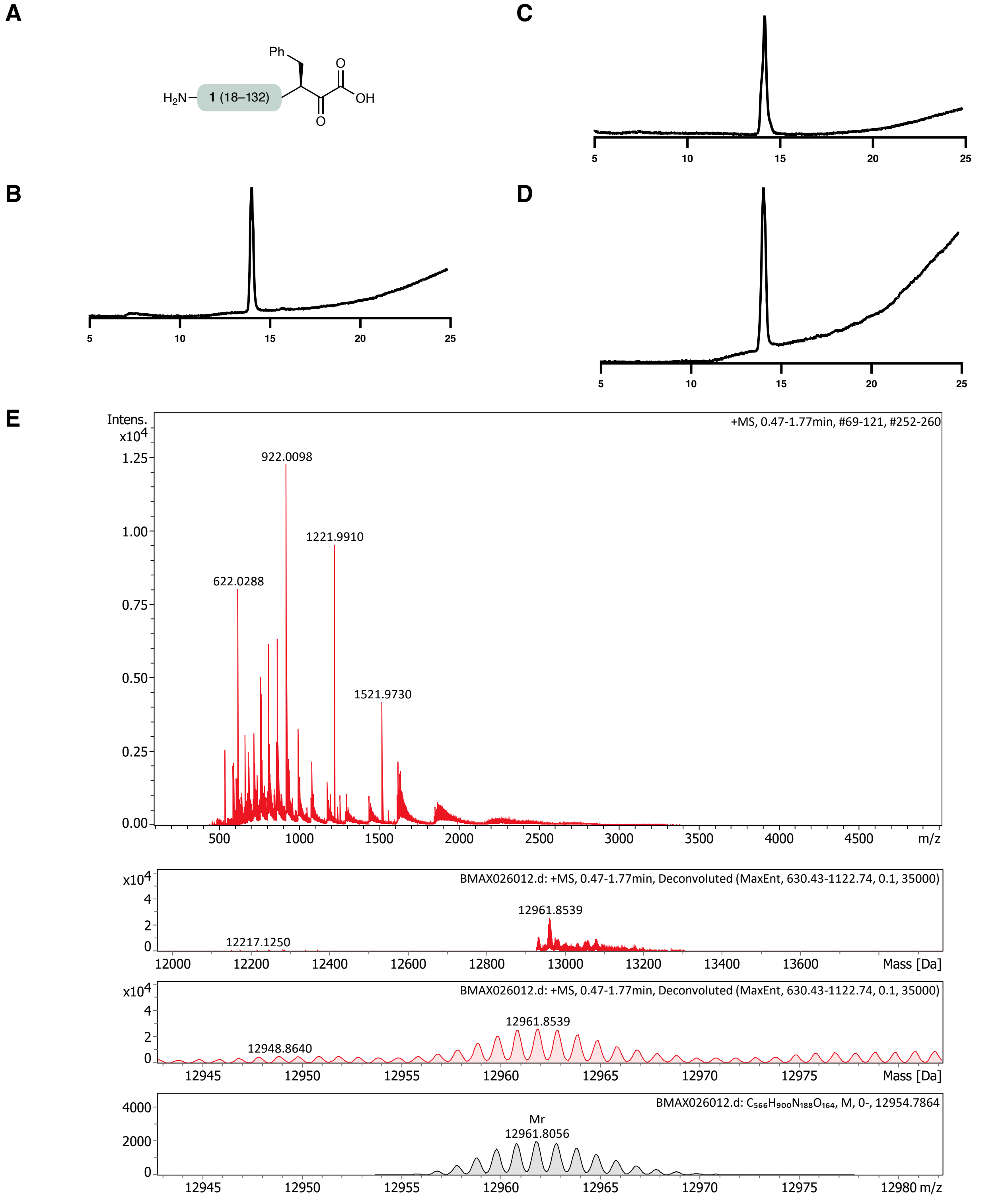

Figure S3. (A) Structure of segment **1c**. (B) HPLC chromatogram of **1c** using analytical A method. (C) HPLC chromtaogram of the crude reaction mixture after quenching with DMS using analytical A method. (D) HPLC chromatogram of purified product using analytical A method. (E) HR-MS (ESI), found: 12961.8539 (second and third panels), calculated for $C_{566}H_{900}N_{188}O_{164}$ [M] 12961.8056 (fourth panel).

6 SPPS

Segment 2

MBHA rink amide resin (0.56 mmol/g, 100-200 mesh, Iris Biotech GMBH) was preloaded with Fmoc-Phe-α-ketoacid-OMe-linker, giving an Fmoc determined loading of 0.18 mmol/g, 0.55 g, 100 μmol. After performing SPPS, the resin was processed according to the cleavage protocol. The product was purified by RP-HPLC using Prep A method and lyophilized to afford a white powder (71 mg, 13 μmol, 13% yield).

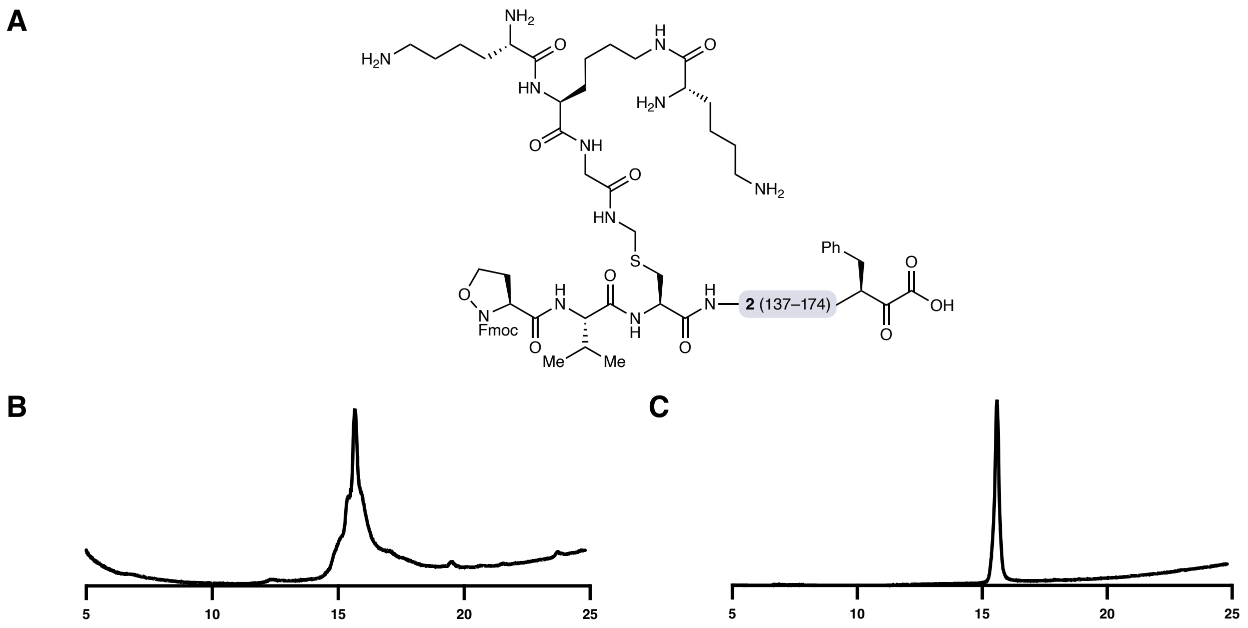

D

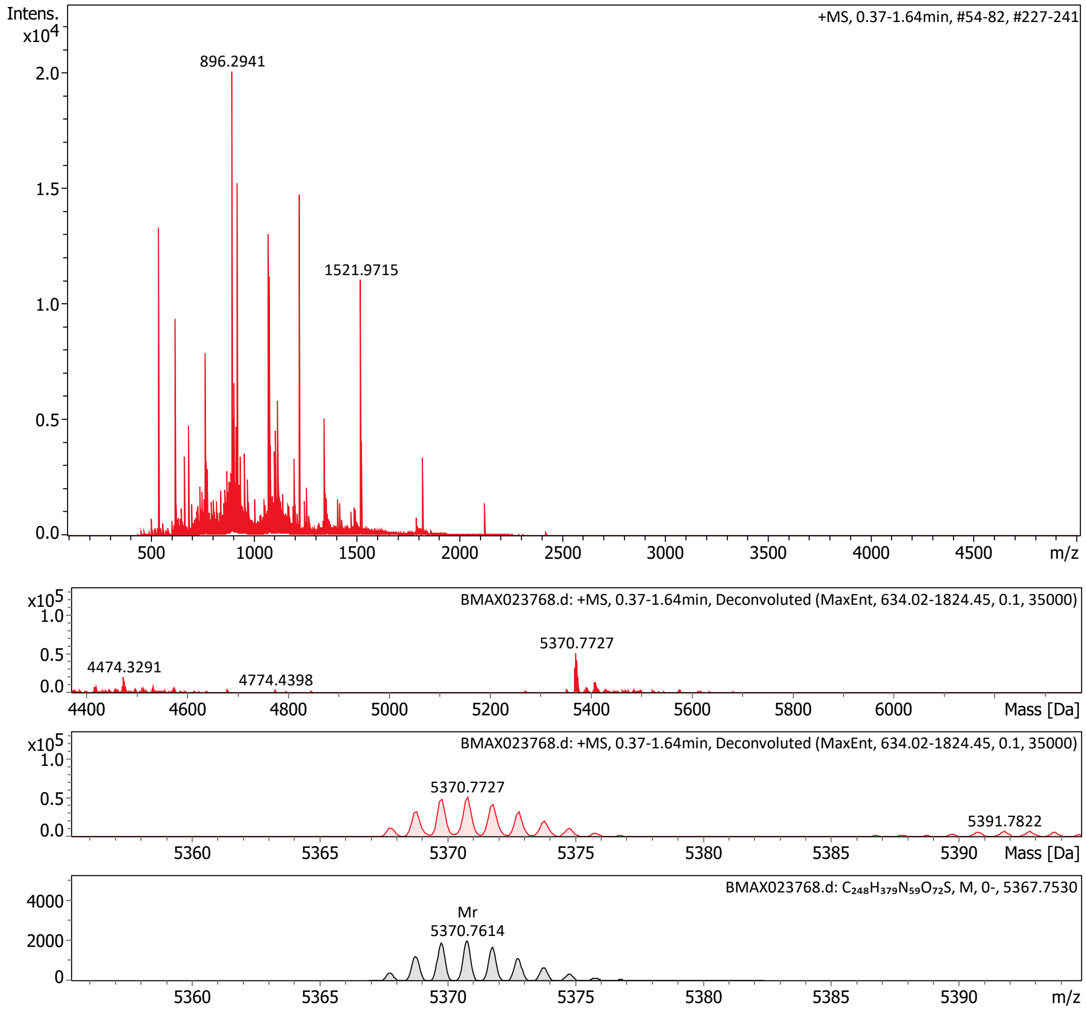

E

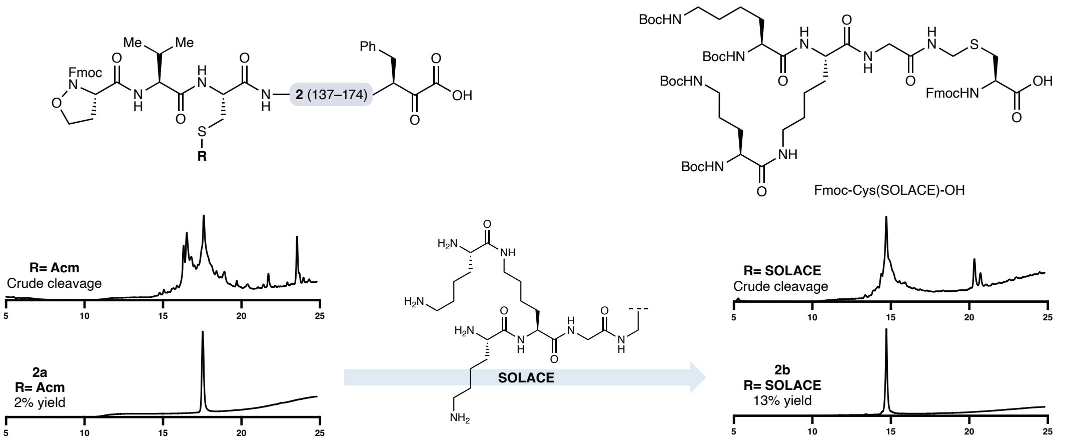

Figure S4. (A) Structure of segment **2**. (B) HPLC chromatogram of crude peptide cleavage using analytical A method. (C) HPLC chromatogram of purified product using analytical A method. (D) HR-MS (ESI) found: 5370.7727 (second and third panels), calculated for $C_{248}H_{379}N_{59}O_{72}S$ [M]: 5370.7614 (fourth panel). (E) Comparison of Acm and SOLACE on segment **2** retention time and yield using HPLC analytical B method.

Segment 3

MBHA rink amide resin (0.56 mmol/g, 100-200 mesh, Iris Biotech GMBH) was preloaded with Fmoc-Leu-α-ketoacid-OMe-linker, giving an Fmoc determined loading of 0.28 mmol/g, 3.20 g, 0.90 mmol. After performing SPPS, the resin was processed according to the cleavage protocol. The product was purified by RP-HPLC using Prep A method and lyophilized to afford a white powder (784 mg, 174 μmol, 19% yield).

A

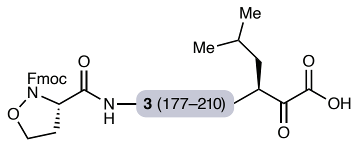

B

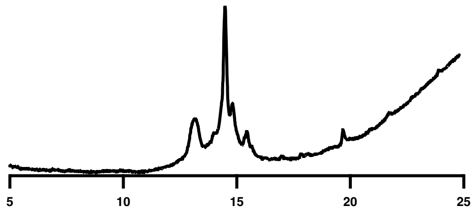

C

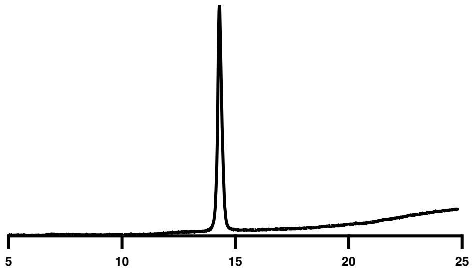

D

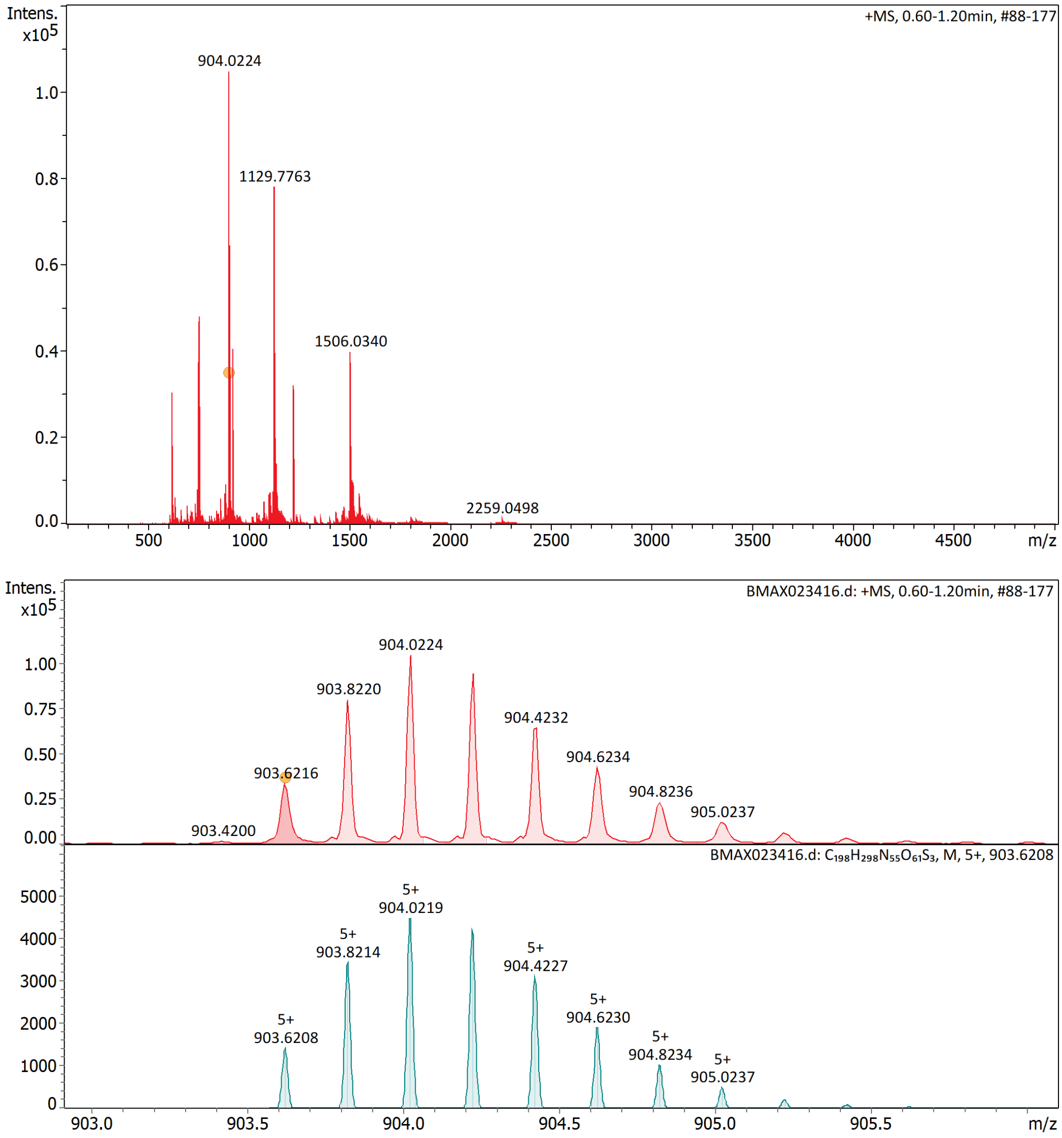

Figure S5. (A) Structure of segment **3**. (B) HPLC chromatogram of crude peptide cleavage using analytical A method. (C) HPLC chromatogram of purified product using analytical A method. (D) HR-MS (ESI) found 904.0224 (second panel), calculated for $C_{198}H_{298}N_{55}O_{61}S_3$ $[M+5H]^{5+}$: 904.0219 (third panel).

Segment 4

2-Chloro-trityl resin (1.5 mmol/g, 100-200 mesh, Iris Biotech GMBH) was preloaded with Fmoc-Ala-OH, giving an Fmoc determined loading of 0.35 mmol/g, 2.3 g, 0.80 mmol. After performing SPPS, the resin was processed according to the cleavage protocol. The product was purified by RP-HPLC using Prep A method and lyophilized to afford a white powder (190 mg, 54 μmol, 7% yield).

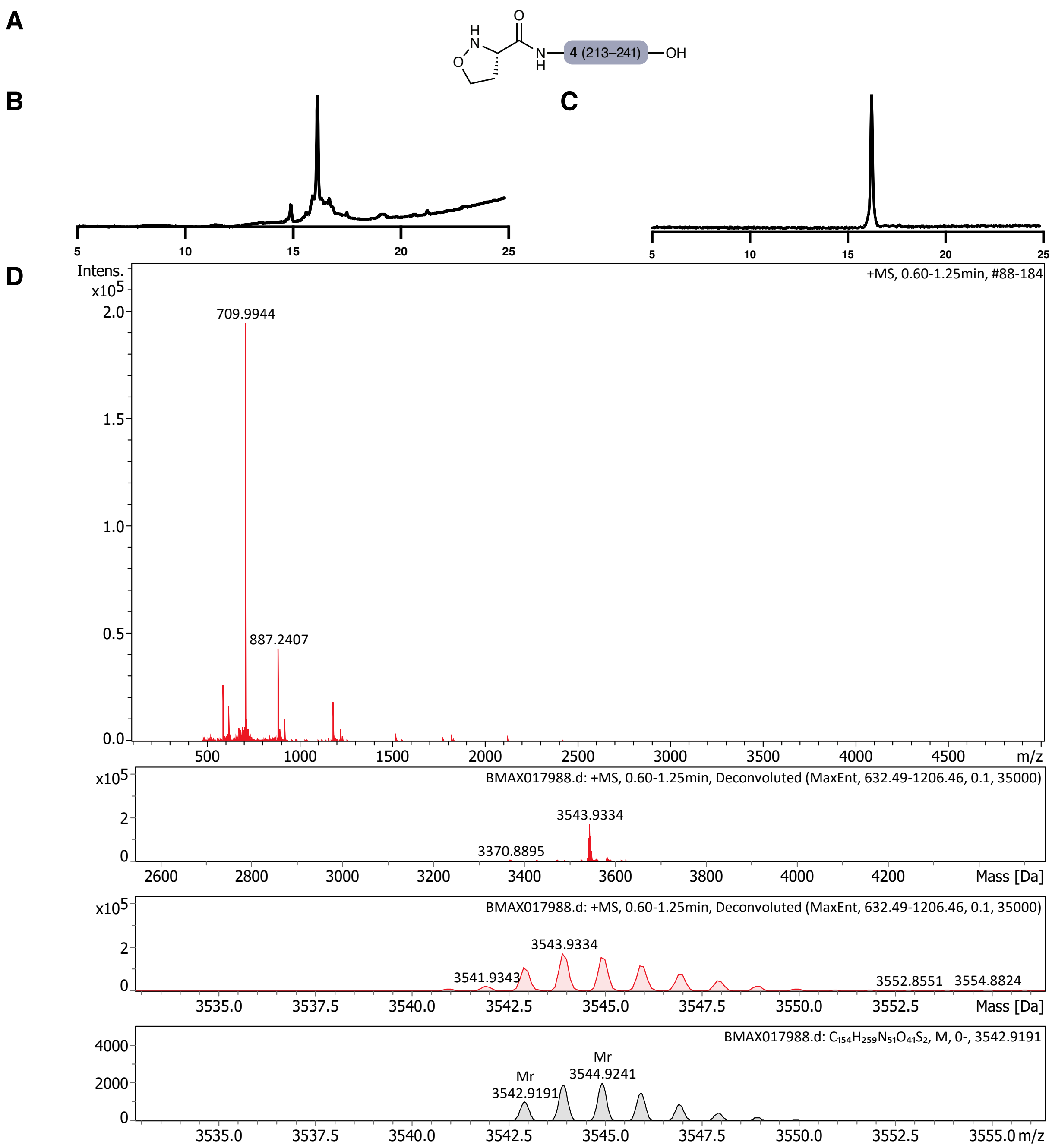

Figure S6. (A) Structure of segment **4**. (B) HPLC chromatogram of the crude crude peptide cleavage using analytical A method. (C) HPLC chromatogram of the purified product using analytical A method. (D) HR-MS (ESI) found 3543.994 (second and third panels), calculated for $C_{154}H_{259}N_{51}O_{41}S_2$ [M]: 3543.9191 (fourth panel).

7 Ligations and Deprotections

Synthesis of Segment 3-4

Segment **4** (76 mg, 21 μmol, 1.0 equiv) and segment **3** (115 mg, 26 μmol, 1.2 equiv) were weighed into a Falcon tube. DMSO/H_2O (9:1 + 0.1 M oxalic acid, 1066 μL) was added and the reaction was heated to 60 °C for 22 h. The reaction mixture was diluted with DMSO (19.5 mL) and $NHEt_2$ (2.1 mL) was added. The reaction mixture was shaken at rt for 10 min then quenched with TFA (4.2 mL). The product **3-4** was purified by RP-HPLC by Prep A method and lyophilized to afford a white powder (122 mg, 15.7 μmol, 74% yield).

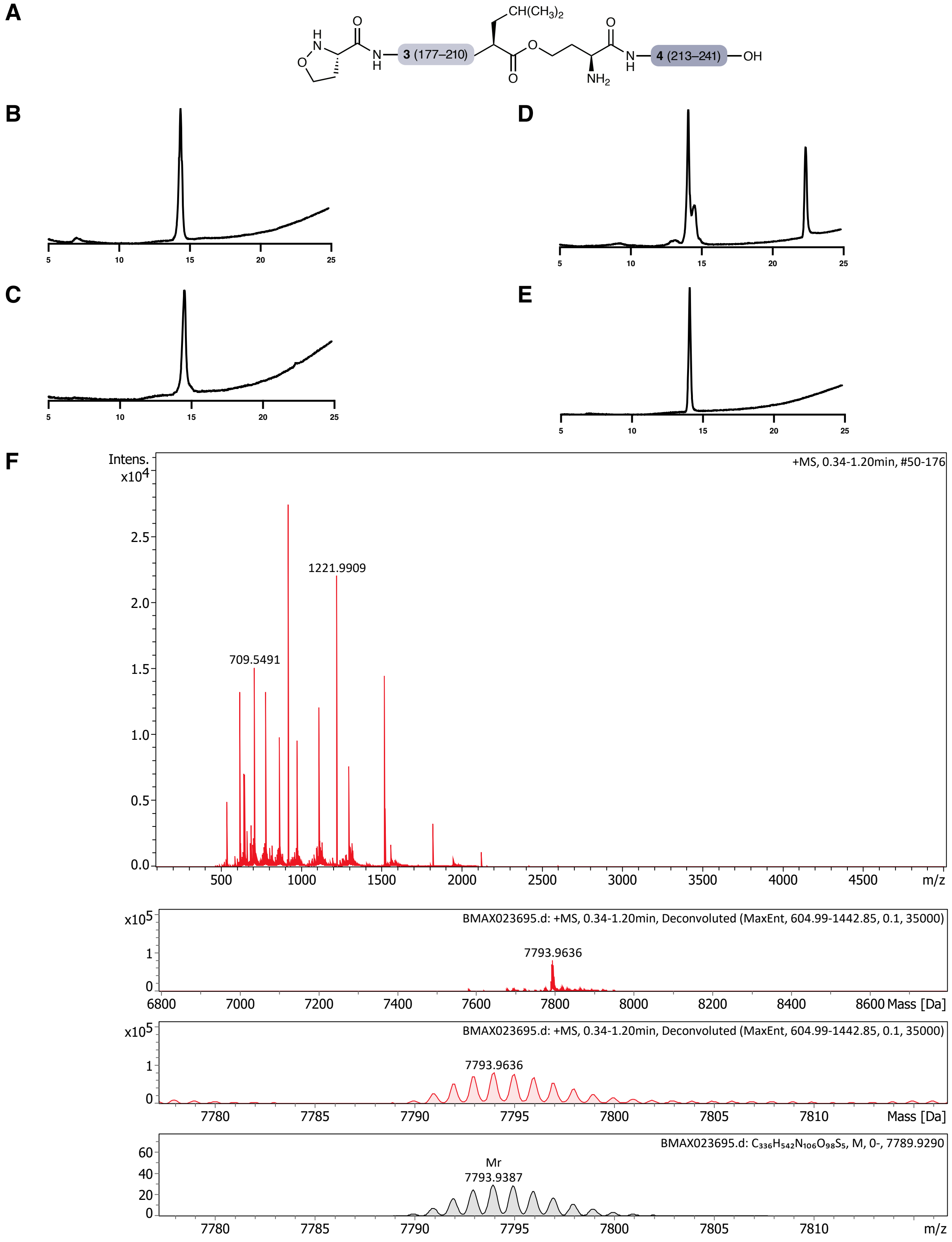

Figure S7. (A) Structure of segment **3-4**. (B) HPLC chromatogram of the crude reaction mixture at time 0 h using analytical A method. (C) HPLC chromatogram of the crude reaction mixture at time 22 h using analytical A method. (D) HPLC chromatogram of the crude reaction mixture after Fmoc deprotection using analytical A method. (E) HPLC chromatogram of the purified product using analytical A method, (F) HR-MS (ESI), found: 7793.9636 (second and third panels), calculated for $C_{336}H_{542}N_{106}O_{98}S_5$ [M]: 7793.9387 (fourth panel).

Synthesis of Ligation Product 2-3-4

Segment **3-4** (39.2 mg, 5.03 μmol, 1.0 equiv) and segment **2** (27.0 mg, 5.03 μmol, 1.0 equiv) were weighed into a Falcon tube. HFIP/AcOH (2:1, 335 μL) was added and the reaction mixture was heated to 45 °C for 4 h. The reaction mixture was diluted with DMSO (4.5 mL) and $HNEt_2$ (503 μL) was added. The reaction mixture was shaken at rt for 10 min then quenched with TFA (1.0 mL). The product **2-3-4** was purified by RP-HPLC using Prep A method and then lyophilized to afford a white powder (25.4 mg, 1.97 μmol, 39% yield).

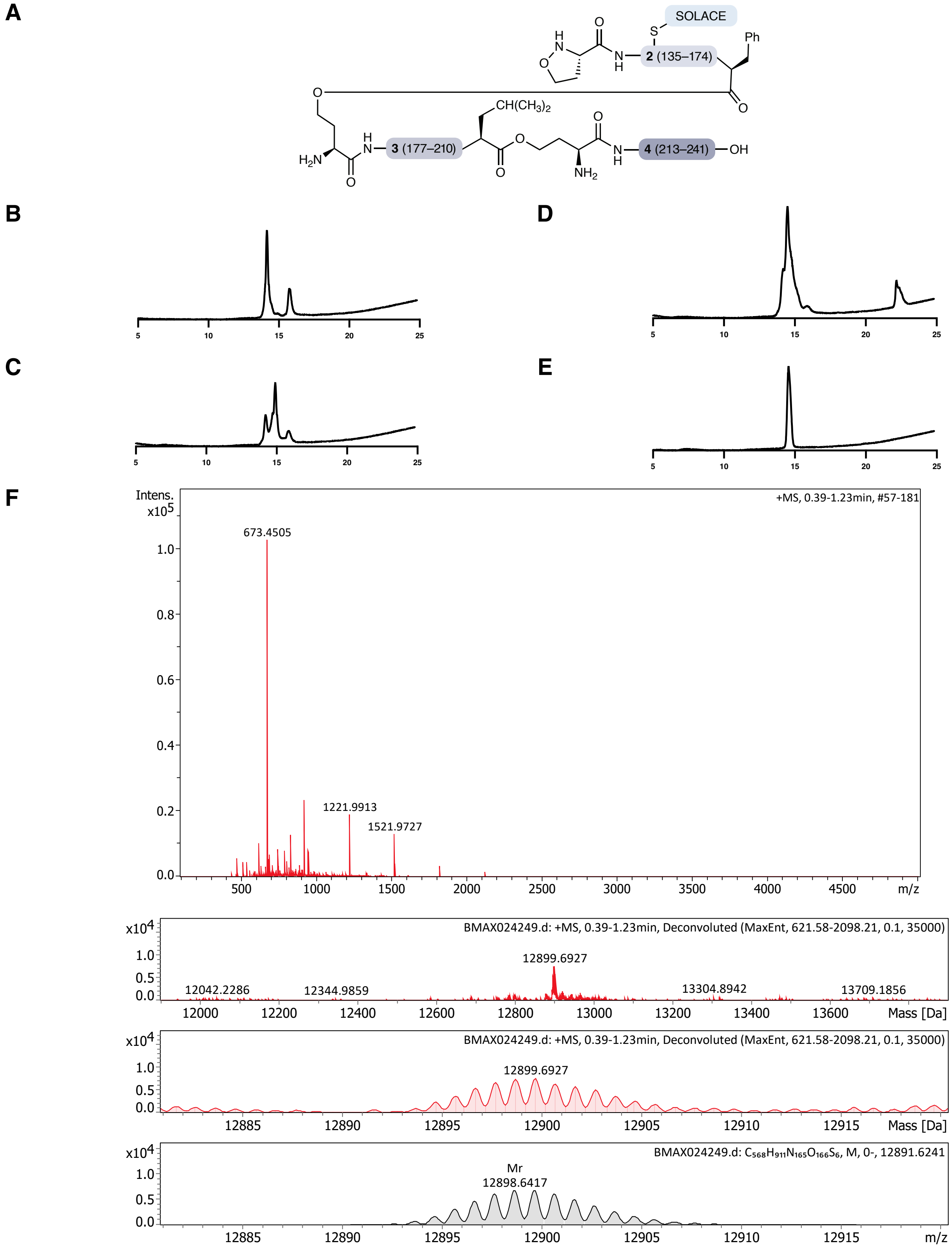

Figure S8. (A) Structure of segment **2-3-4**. (B) HPLC chromatogram of the crude reaction mixture at time 0 h using analytical A method. (C) HPLC chromatogram of the crude reaction mixture at time 4 h using analytical A method. (D) HPLC chromatogram of the crude Fmoc deprotection using analytical A method. (E) HPLC chromatogram of the purified product using analytical A method. (F)

HR-MS (ESI), found 12899.6927 (second and third panels), calculated for $C_{568}H_{911}N_{165}O_{166}S_6$ [M]: 12891.6241 (fourth panel).

Synthesis of *depsi*-proNGF 5a

Segment **1c** (22 mg, 1.70 μmol, 2.0 equiv) and **2-3-4** (11 mg, 0.85 μmol, 1.0 equiv) were dissolved in HFIP/AcOH (2:1, 85 μL) and heated to 45 °C for 15 h. The mixture was diluted with AcOH/Milli-Q water (1:1, 3.9 mL), AgOAc (39.7 mg, 238 μmol, 280 equiv) was added, and the reaction mixture heated to 50 °C in the dark for 2 h. A 50% aq. acetic acid solution containing 10% DTT (2.0 mL) was added to the mixture, and the formed precipitate was removed by centrifugation. The precipitate was washed with the same solution (2.0 mL). The combined supernatant containing *depsi*-proNGF **5a** was purified by RP-HPLC using Prep A method and lyophilized to afford a white solid (8.5 mg, 0.34 μmol, 40% yield).

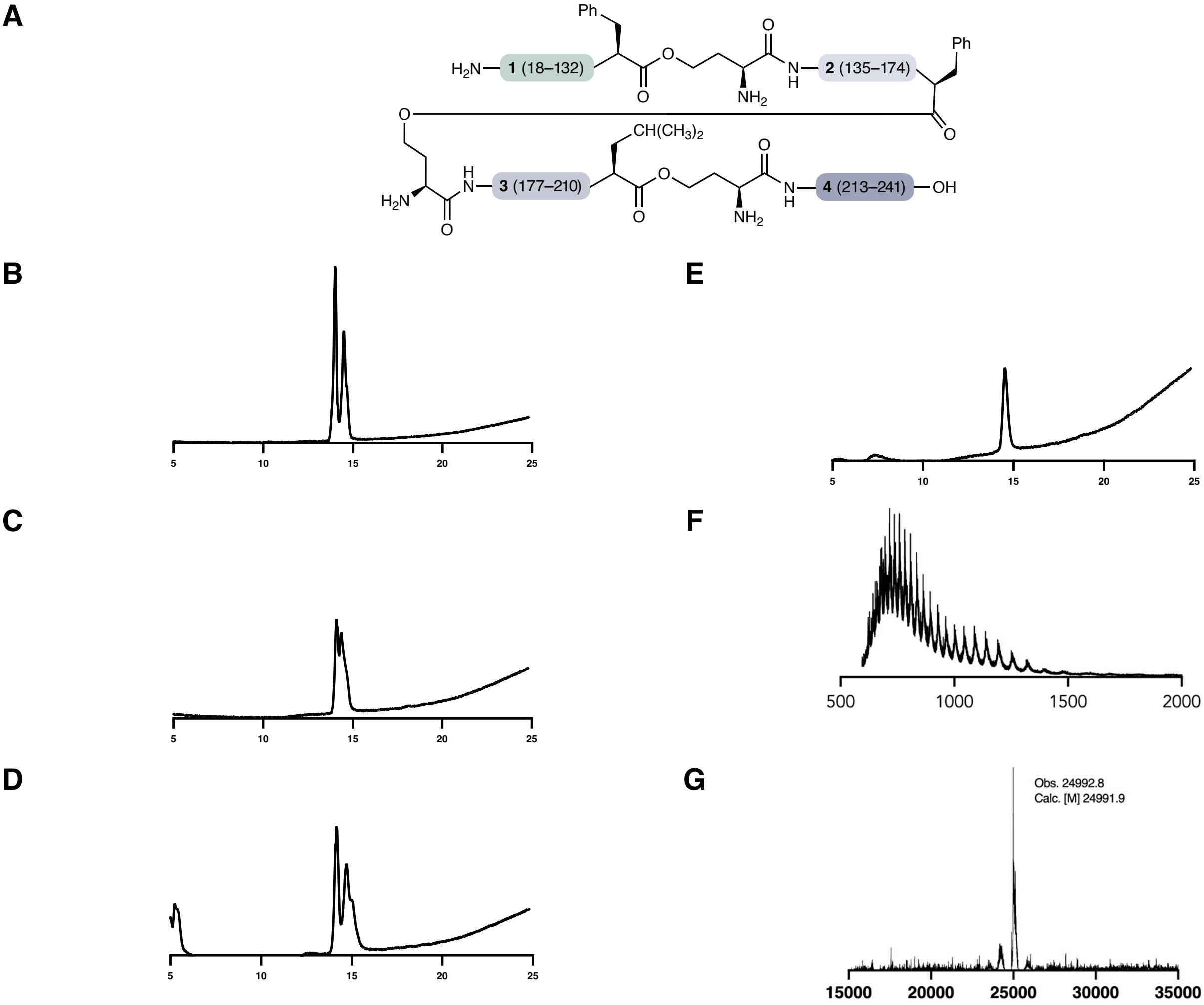

Figure S9. (A) Structure of proNGF **5a**. (B) HPLC chromatogram of the crude reaction mixture at time 0 h using analytical A method. (C) HPLC chromatogram of the crude reaction mixture at time 15 h using analytical A method. (D) HPLC chromatogram of the crude cysteine deprotection using analytical A method. (E) HPLC chromatogram of purified product using analytical A method. (F) MS

(ESI) of proNGF **5a**. (G) Deconvoluted MS (ESI) of *depsi*-proNGF **5a**, found 24992.8, calculated for $C_{1097}H_{1744}N_{340}O_{319}S_6$ [M]: 24991.9.

8 Foldings

Folding of proNGF 5c

Depsi-proNGF **5a** (1.2 mg, 48 nmol) was denatured in 4.8 mL of GdnHCl 6 M, TrisHCl 100 mM, EDTA 100 mM, DTT 1 M, pH 8. After incubating for 2.5 h at 37 °C, DTT was removed by dialyzing the sample against GdnHCl 6 M, EDTA 10 mM, 45 mL x 3. Folding of rearranged proNGF **5b** was induced by dialyzing the sample into TrisHCl 100 mM, L-arginine 0.75 M, GSH 5 mM, GSSG 0.5 mM, EDTA 5 mM pH 9.5 at 4 °C, 45 mL x 4 over 24 h. The product was purified by RP-HPLC using Semi-Prep Folding method and lyophilized (272 μg, 11 nmol, 23% yield).

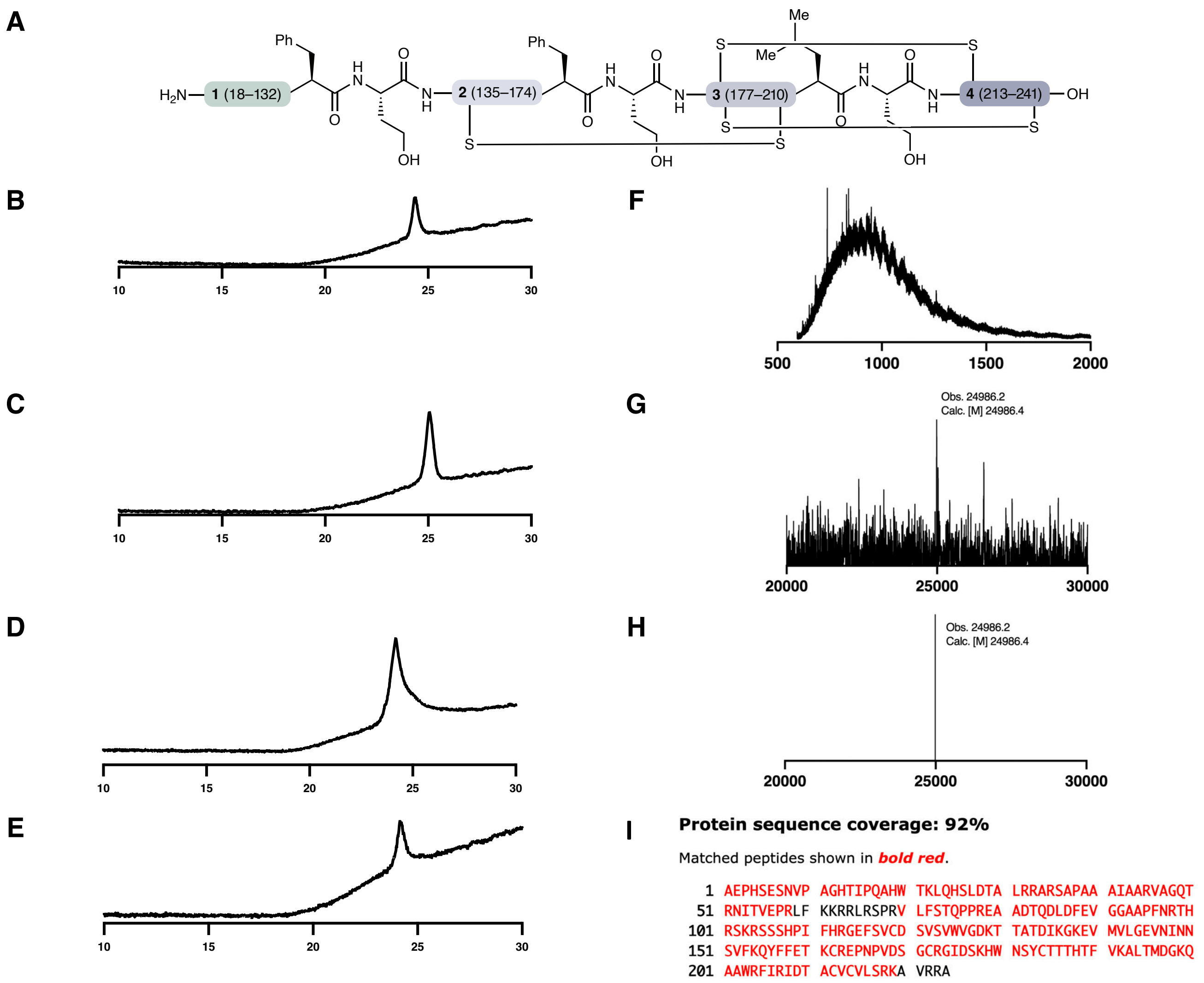

Figure S10. (A) Structure of folded proNGF **5c**. (B) HPLC chromatogram of *depsi*-proNGF **5a** using analytical folding A method. (C) HPLC chromatogram of rearranged proNGF **5b** using analytical folding A method. (D) HPLC chromatogram after folding for 24 h using analytical folding A method. (E) HPLC chromatogram of **5c** using analytical folding A method. (F) MS (ESI) of **5c**. (G) Deconvoluted MS (ESI) of **5c**, found 24986.2, calculated for $C_{1097}H_{1738}N_{340}O_{319}S_6$ [M]: 24986.4. (H) MaxEntX deconvoluted MS (ESI) of **5c**, found 24986.2, calculated for $C_{1097}H_{1738}N_{340}O_{319}S_6$ [M]: 24986.4. (I) In-solution tryptic digestion and MS/MS analysis of **5c** yielded a sequence coverage of 92%.

Folding comparisons of rh-proNGF, syn-proNGF, and syn-βNGF

In this comparative study, the synthetic proNGF did not contain the D56E and D164E mutations, and the synthetic βNGF did not contain the D164E mutation. Rh-proNGF, syn-proNGF, and syn-βNGF were prepared, denatured, and folded as described in McMillan.[6] See section *12 Protein Sequences* for rh-proNGF sequence.

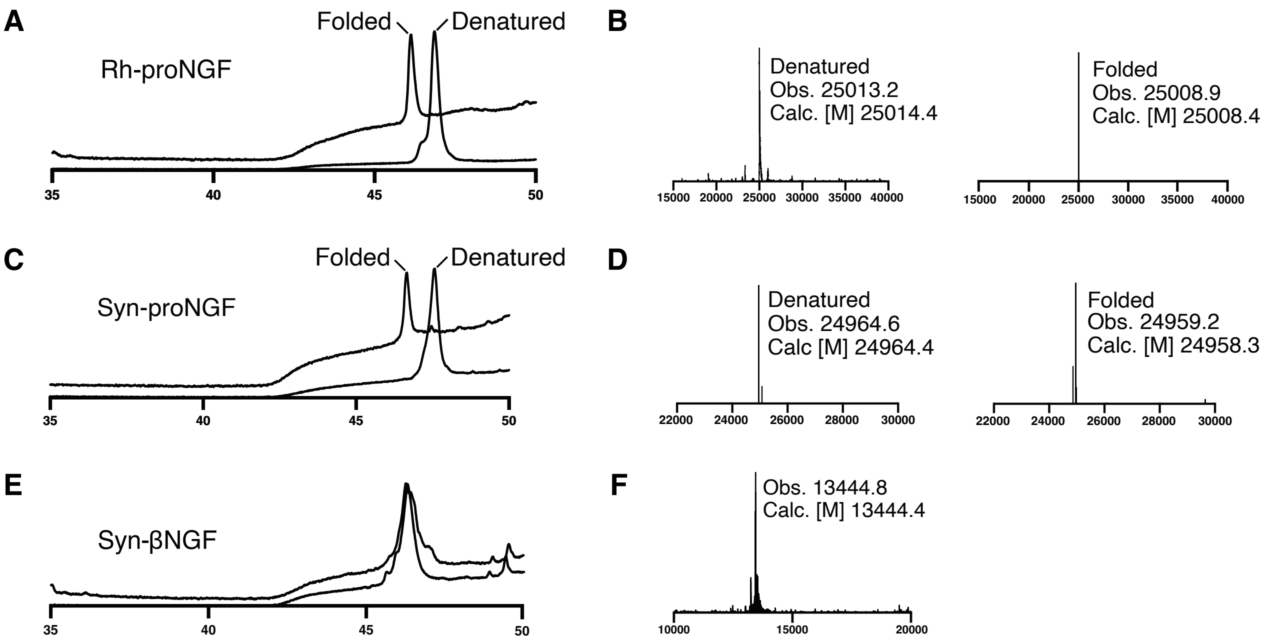

Figure S11. (A) HPLC comparison of denatured and folded recombinant proNGF using analytical folding B method. (B) MaxEntX deconvoluted MS (ESI) of denatured and folded recombinant proNGF. (C) HPLC comparison of denatured and folded synthetic proNGF using analytical folding B method. (D) MaxEntX deconvoluted MS (ESI) of denatured and folded synthetic proNGF. (E) HPLC analysis comparing denatured synthetic βNGF and its folding after 7 days using analytical folding method B revealed no detectable folded βNGF. (F) MaxEntX deconvoluted MS (ESI) of synthetic βNGF.

(6) McMillan, A. E. Automated Chemical Systems and the Synthesis of Human Nerve Growth Factor. (ETH Zurich, **2023**). doi:10.3929/ethz-b-000637508.

9 Enzymatic Processing

βNGF 6

Crude folded proNGF **5c** (261 μg/mL, 500 μL) in TrisHCl 100 mM, L-arginine 0.75 M, GSH 5 mM, GSSG 0.5 mM, EDTA 5 mM pH 7.8 was transferred into an Eppendorf tube. Trypsin (6 μg, 1:209 (w/w)) in 6 μL 50 mM AcOH was added, and the mixture was incubated at 4 °C for 14 h. The mixture was purified by SEC and the product (βNGF **6**) was desalted using Pierce C18 spin columns and lyophilized (12.7 ug, 0.94 nmol, 18% yield).

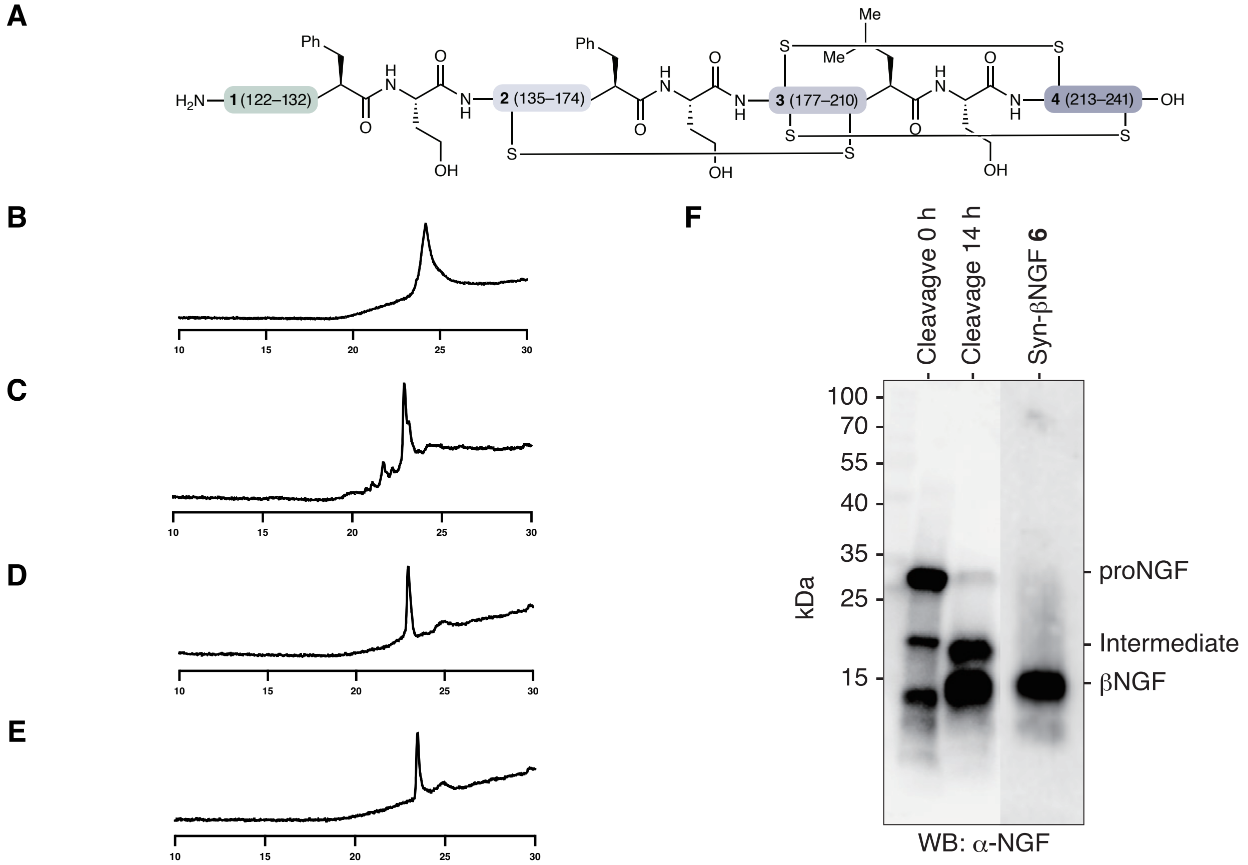

Figure S12. (A) Structure of folded βNGF **6**. (B) HPLC chromatogram of folded proNGF **5c** using analytical folding A method. (C) HPLC chromatogram of crude reaction mixture at time 14 h using analytical folding A method. (D) HPLC chromatogram of **6** using analytical folding A method. (E) HPLC chromatogram of recombinant βNGF using analytical folding A method. (F) Western blot of the crude reaction mixture at time 0 h and 14 h, and isolated **6**.

Comparison of Furin and Trypsin in Enzymatic Processing

In this comparative study, the synthetic proNGF did not contain the D56E and D164E mutations and was prepared as described in McMillan.[6]

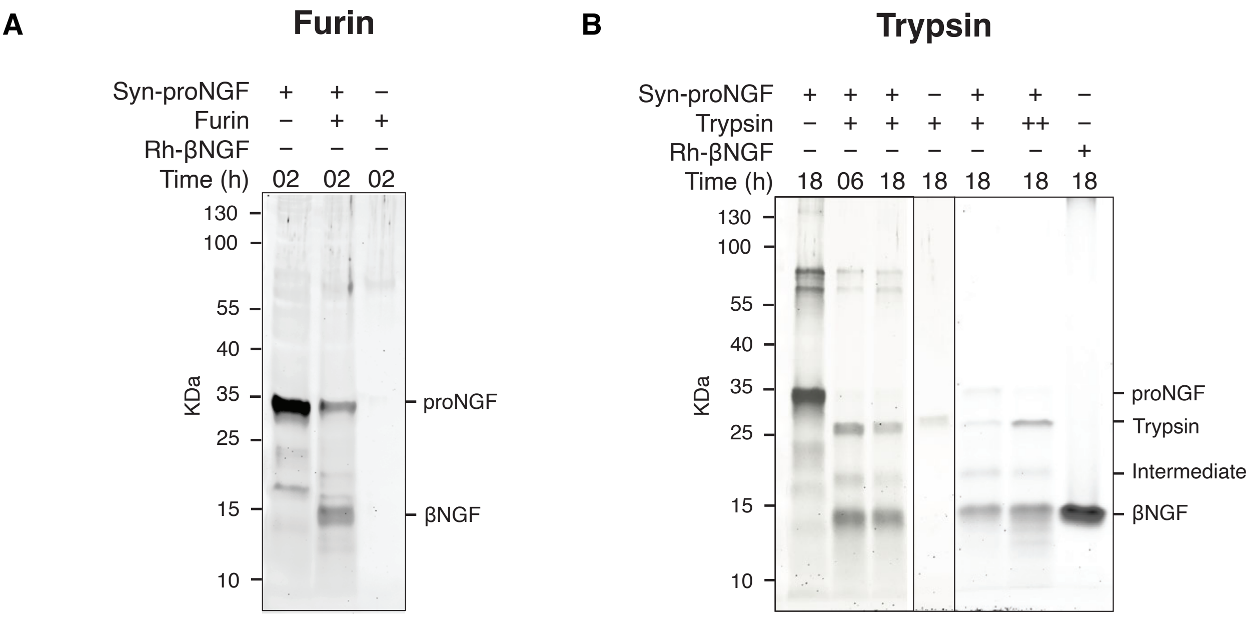

C

Furin Lane	1	2	3
ProNGF, 0.8 µg/mL in NaPhos, pH 7.4 (µL)	2	2	0
Furin, 2000 U/mL in 100 mM HEPES, 4 mM $CaCl_2$, pH 7.4 (µL)	0	1	1
Buffer (100 mM HEPES, 4 mM $CaCl_2$, pH 7.4) (µL)	8	7	9

D

Trypsin Lane	1	2	3	4	5	6	7
ProNGF, 0.8 µg/mL in NaPhos, pH 7.4 (µL)	2	2	2	0	2	2	Commercial βNGF (2 µL)
Trypsin, 1 µg/mL in 50 mM AcOH (µL)	0	0.<br>2	0.<br>2	0.<br>2	0.<br>2	0.<br>2	0
Buffer (25 mM NH_4CO_3, pH 9) (µL)	2	1.<br>8	1.<br>8	3.<br>8	1.<br>8	1.<br>6	0
Time (h) on ice	18	6	18	18	18	18	18

Figure S13. (A) SDS-page of furin digests using SYPRO Ruby gel stain. (B) SDS-page of trypsin digests using SYPRO Ruby gel stain. (C) Volumes of stock solutions for furin samples combined and incubated at 30 °C for 2 h. (D) Volumes of stock solutions for trypsin samples that were combined and incubated on ice for the the specified duration, then quenched with 0.2 µL PMSF (100 mM in EtOH).

10 *In vitro* Axon Growth Assay

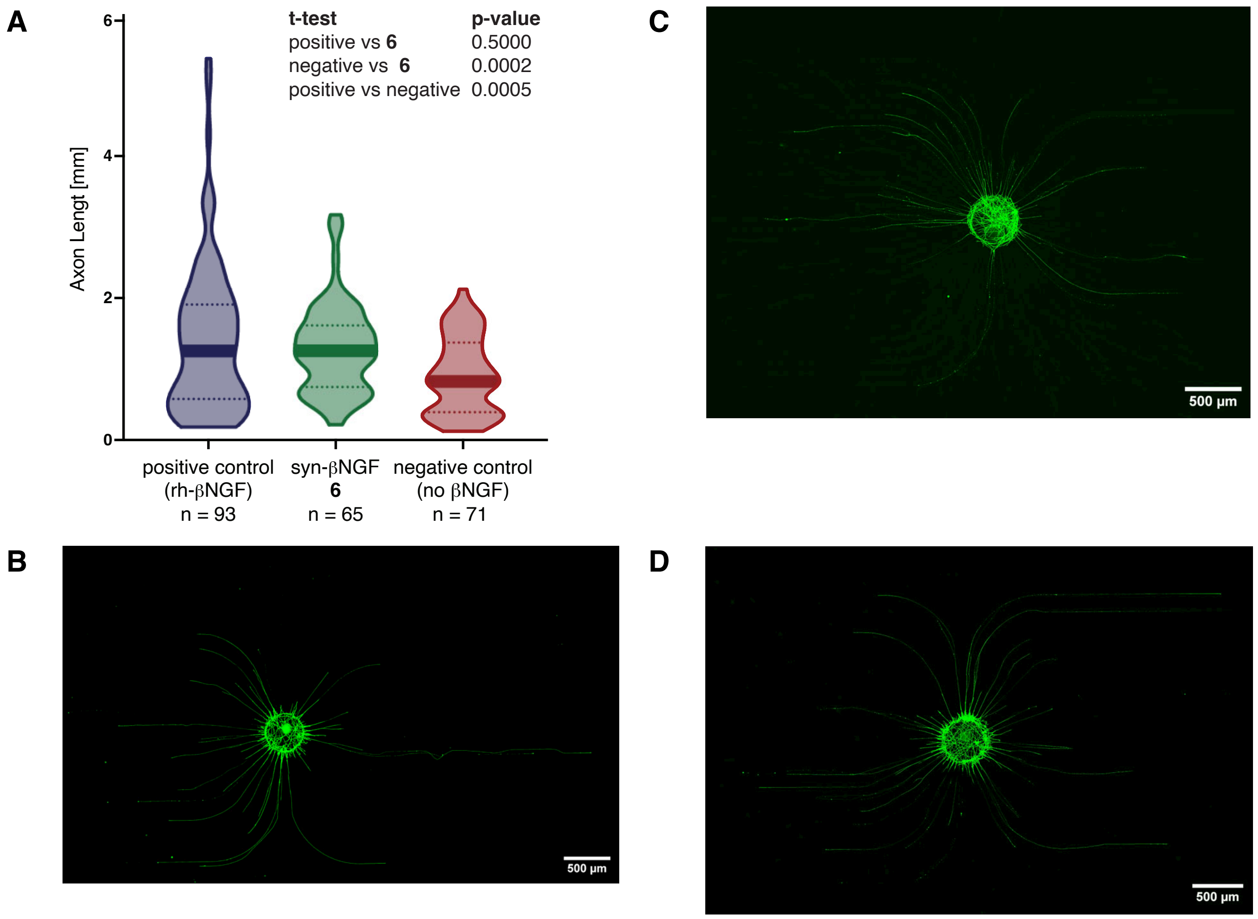

Figure S14. (A) *In vitro* comparison of axon growth in human iPSC-derived neurons treated with recombinant βNGF, synthetic βNGF **6**, and a control group without βNGF. (B) Axon growth in a neuron treated with synthetic βNGF **6**. (C) Axon growth in the control group without βNGF. (D) Axon growth in a neuron treated with recombinant βNGF.

PDMS Microstructures

PDMS microstructures for guiding cells and axons were designed using CAD software (AutoCAD 2021) and fabricated by Wunderlichips (Zurich, Switzerland), following the fabrication methods outlined in previous publications.[7,8] These microstructures have a total thickness of 200 µm and feature a central well with a diameter of 400 µm. The well is open at the top and extends into 6 to

(7) Forró, C.; Thompson-Steckel, G.; Weaver, S.; Weydert, S.; Ihle, S.; Dermutz, H.; Aebersold, M. J.; Pilz, R.; Demkó, L.; Vörös, J. Modular microstructure design to build neuronal networks of defined functional connectivity. *Biosens. and Bioelectron.* **2018**, *122*, 75–87.

(8) Mateus, J. C.; Weaver, S.; van Swaay, D.; Renz, A. F.; Hengsteler, J.; Aguiar, P.; Vörös, J. Nanoscale patterning of in vitro neuronal circuits. *ACS Nano* **2022**, *16*, 5731–5742.

60 microchannels, which are covered on top and measure 4 µm in height, 50 µm in width, and 8 mm in length. Additional details on these microstructures can be found in Vulić *et al.*[9]

Substrate Preparation

A sterile µ-Dish with a 35 mm glass bottom (Ibidi, Germany) was coated with 500 µL of 0.01% Poly-L-Ornithine (PLO) solution (A-004-M, 81158, Sigma- Aldrich, Switzerland) for 30 minutes at room temperature. After coating, the PLO was rinsed three times with ultrapure water (PURELAB Flex, Elga Lab- water, United Kingdom), and the dish was then carefully dried using a nitrogen gun. Following this, the PDMS microstructure was cut with a scalpel and po- sitioned onto the dried, PLO-coated substrate using tweezers. The assembled substrate was placed in an oven at 37 °C for 30 minutes to ensure proper at- tachment of the microstructure. Phosphate buffered saline (PBS) (10010-023, ThermoFisher) was then added to the dishes, which were placed in a desiccator for approximately 10 minutes or until no air bubbles were visible from the microstructures. After desiccation, the PBS was replaced with a Laminin-based solution (iMatrix-511 Silk, Anatomic Inc., USA) and left overnight for secondary coating. The following day, the Laminin solution was replaced with 500 µL of the initial cell culture medium, Senso-MM (Anatomic, USA).

Cell Culture

The cells used in the experiments are human induced pluripotent stem cell derived (hiPSC) RealDRG™ Sensory Neurons (Anatomic, USA).

Cell Thawing

Cells are kept frozen in liquid nitrogen until usage. A frozen aliquot of sensory neurons was taken out of liquid nitrogen and rapidly thawed at 37 °C. The 1 mL thawed cell solution was transferred dropwise into 3 mL of warm Senso- MM medium and centrifuged for 5 min at 200 g. Cells were re-suspended in Senso-MM at a concentration of 1x106 cells/mL.

Spheroid Preparation

Spheroids were prepared as previosuly described in Vulić *et al.*[9] Namely, after cell counting the volume of cell suspension needed to create spheroids containing 100 cells each was added to an AggreWell microwell plate (Stemcell Technologies Inc., Canada). The wells were then filled with cell medium Senso-MM up to approximately 2 mL. The AggreWell plate was centrifuged for 3 minutes

(9) Vulić, K.; Amos, G.; Ruff, T.; Kasm, R.; Ihle, S. J.; Küchler, J.; Vörös, J.; Weaver, S. Impact of microchannel width on axons for brain-on-chip applications. *Lab Chip* **2024**, *24*, 5155–5166.

to ensure uniform cell distribution and facilitate spheroid formation. Once prepared, the AggreWell plate was placed in an incubator until the spheroids were ready for seeding.

The day after dissociation, spheroids formed in the AggreWells were ready for transfer into the PDMS microstructures. For seeding, 0.5 mL of the spheroid solution was carefully transferred to a small Petri dish to maintain the rest of the AggreWell at a stable temperature with CO_2 levels. Next, 5–10 spheroids were aspirated from the Petri dish using a 10 µL pipette, and each spheroid was carefully pipetted into the central wells of the microstructures one by one. After all seeding spots were filled (but within 10 minutes of removal from the incubator), the culture dish (either a glass dish or a microelectrode array) was placed back in the incubator, set to 37 °C, 90% humidity, and 5% CO_2. For experiments involving spheroids, the day of seeding into the PDMS microstructures was recorded as day *in vitro* (DIV) zero.

Cell Maintenance

Cultures with cells confined inside the PDMS microstructures contained ~1 mL of medium. This was sufficient to completely cover the PDMS microstructures and allow for passive nutrient diffusion inside the microchannels. Twice a week the cell medium was exchanged from the dish under the laminar flow hood by pipetting out ~0.5 mL of the old medium from the edge of the dish and adding ~0.6 mL of the fresh warm medium. Two types of medium were used as instructed by the cell provider. In the initial culturing phase (DIV 0-3) cell medium senso-MM was used, and in the second culturing phase (¿DIV 3) cell medium Senso-MMx2 (Anatomic, USA) was used. From DIV 0, medium was supplemented with neural growth factor (NGF) depending on the experimental condition. In all experimental conditions where NGF was used, NGF diluted in the 0.1 % bovine serum albumin (BSA) solution in PBS was added to the culture to reach a final concentration of 50 ng/mL. In the first condition, commercially available Human βNGF Recombinant Protein, (PeproTech 450-01, ThermoFisher) was added. In the second condition synthetic βNGF **6** was added. In the third condition, medium was not supplemented with βNGF.

Fluorescent Labelling

On the day of imaging, a 2 µm solution of calcein AM (L3224, ThermoFisher) in Dulbecco's PBS (DPBS) (14190-144, ThermoFisher) was added in culture and left in the incubator for 20 min. For some experiments, 8 µM of ethidium homodimer-1 (L3224, ThermoFisher) was added. After incubation, sample was washed once with DPBS and left in warm DPBS for imaging.

Image Acquisition and Analysis

Stained cultures were imaged using a confocal laser scanning microscope (CLSM) (Fluoview 3000, Olympus). The images were acquired using a 10x objective (Olympus, UPLFLN10X2PH, NA = 0.3) with a laser wavelength of 488 nm (for Calcein-stained cultures) and 561 nm (for ethidium homodimer-1 stained cultures). Acquired images were analysed using Fiji and custom-made Python scripts.[10] To assess the length of axon growth in the microchannels, images were overexposed and the length of the segmented line drawn on top of the axon was measured, as described in Vulić *et al.*[9] The collected data points are presented in the table below.

Positive Control Recombinant βNGF			Synthetic βNGF (**6**)			Negative Control No βNGF		
Sample	Length [μm]	Div	Sample	Length [μm]	Div	Sample	Length [μm]	Div
1	967.08	15	1	3005.215	15	1	920.944	15
1	2058.893	15	1	655.913	15	1	362.279	15
1	746.234	15	1	1479.077	15	1	369.799	15
1	5041.557	15	1	1822.31	15	1	324.187	15
1	578.752	15	1	1622.243	15	1	1727.713	15
1	3447.158	15	1	1215.683	15	1	184.104	15
1	1753.278	15	1	1404.889	15	1	746.829	15
1	807.733	15	1	1084.699	15	1	1159.071	15
1	5373.942	15	1	1318.01	15	1	396.988	15
1	481.475	15	1	724.119	15	1	363.543	15
1	539.279	15	1	1196.326	15	1	433.914	15
1	230.642	15	1	1299.94	15	1	1811.308	15
1	2472.976	15	1	691.763	15	1	406.789	15
1	583.013	15	1	1810.199	15	1	1565.059	15
1	1540.799	15	1	1849.368	15	1	1111.163	15
1	313.899	15	1	611.267	15	1	1094.299	15
1	473.854	15	1	1158.424	15	1	1602.085	15
1	1912.931	15	1	548.577	15	1	149.229	15
1	2200.303	15	1	1396.243	15	1	665.242	15
1	1444.449	15	2	566.566	15	1	996.104	15
1	1794.395	15	2	1081.85	15	1	1823.374	15
1	1638.596	15	2	513.521	15	1	592.602	15
1	1363.509	15	2	535.007	15	1	816.237	15
1	791.619	15	2	221.547	15	1	840.084	15
1	226.009	15	2	3168.556	15	1	434.02	15
1	348.652	15	2	297.868	15	1	196.203	15
1	742.31	15	2	1923.865	15	1	1680.304	15
1	583.813	15	2	2565.371	15	1	2122.518	15
1	1127.942	15	2	1589.268	15	1	1261.856	15
2	2610.302	15	2	1067.03	15	1	774.699	15
2	1851.805	15	2	796.236	15	1	1278.862	15

(10) Schindelin, J.; Arganda-Carreras, I.; Frise, E.; Kaynig, V.; Longair, M.; Pietzsch, T.; Preibisch, S.; Rueden, C.; Saalfeld, S.; Schmid, B.; Tinevez, J.-Y.; White, D. J.; Hartenstein, V.; Eliceiri, K.; Tomancak, P.; Cardona, A. Fiji: an open-source platform for biological-image analysis. *Nat. Methods* **2012**, *9*, 676–682.

2	362.286	15
2	1732.613	15
2	1841.703	15
2	1175.008	15
2	201.465	15
2	1691.819	15
2	1883.71	15
2	1214.044	15
2	494.596	15
2	933.387	15
2	394.915	15
2	4310.689	15
2	1494.953	15
2	2518.91	15
2	2644.43	15
2	2181.788	15
2	3346.243	15
2	230.641	15
2	1895.093	15
2	1266.493	15
2	1493.674	15
2	1696.805	15
2	2407.11	15
2	661.154	15
2	234.961	15
2	2112.238	15
2	2172.163	15
2	2188.666	15
3	3374.007	15
3	3194.8	15
3	566.624	15
3	971.009	15
3	916.117	15
3	979.26	15
3	328.605	15
3	536.137	15
3	666.369	15
3	248.023	15
3	809.404	15
3	613.564	15
3	1437.147	15
3	696.604	15
3	726.8	15
3	2347.094	15
3	1411.241	15
3	2554.344	15
3	637.036	15
3	1047.95	15
3	1740.853	15
3	1254.891	15
3	1241.853	15
3	1393.546	15
3	1978.266	15

2	1524.704	15
2	2006.375	15
2	1111.576	15
2	492.432	15
2	1068.428	15
2	770.729	15
2	1602.551	15
2	2188.898	15
2	1025.637	15
2	582.124	15
2	1193.665	15
2	1403.872	15
2	807.868	15
3	698.511	15
3	3000.882	15
3	1500.456	15
3	1923.953	15
3	1680.274	15
3	1179.443	15
3	1412.671	15
3	1118.538	15
3	860.583	15
3	1577.371	15
3	1508.485	15
3	1255.104	15
3	724.6	15
3	1864.457	15
3	1598.358	15
3	1835.491	15
3	1869.12	15
3	660.357	15
3	1255.717	15
3	599.332	15
3	1424.04	15

1	1223.012	15
1	959.054	15
1	1797.507	15
2	1371.947	15
2	760.115	15
2	805.053	15
2	826.369	15
2	1603.767	15
2	932.39	15
2	769.329	15
2	623.329	15
2	1959.918	15
2	813.308	15
2	922.073	15
2	990.061	15
2	1059.419	15
2	1418.807	15
2	1071.839	15
2	1770.971	15
2	762.323	15
2	931.159	15
2	1424.243	15
2	1605.269	15
3	342.363	15
3	760.21	15
3	422.977	15
3	191.158	15
3	286.066	15
3	1278.807	15
3	1674.313	15
3	390.438	15
3	1477.584	15
3	364.865	15
3	180.786	15
3	323.63	15
3	1464.771	15
3	453.639	15
3	217.813	15
3	313.581	15
3	124.122	15

3	2013.669	15	
3	296.977	15	
3	320.89	15	
3	1272.152	15	
3	350.869	15	
3	203.098	15	
3	185.824	15	
3	1460.712	15	
3	1672.381	15	

11 Circular Dichroism Spectra

Recombinant (PeproTech, M = 13494 g/mol) and synthetic βNGF **6** (M = 13452 g/mol) were dissolved in buffer at concentrations (c) of 200 μg/mL and 254 μg/mL, respectively. The CD spectra were measured by a J-715 spectropolarimeter (Jasco, Tokyo) at room temperature with a 1 cm optical path length (d). The absorbance was recorded in units of millidegrees (m°) and converted to mean residue ellipticity (deg.cm^2.dmol^{-1}) using the following formula:

$$[\theta]_{MRW} = MRW \times m° / (10 \times c \times d)$$

The mean residue weight was calculated using the following formula:

$$MRW = M / (N - 1)$$

with N = 120 being the number of amino acids.

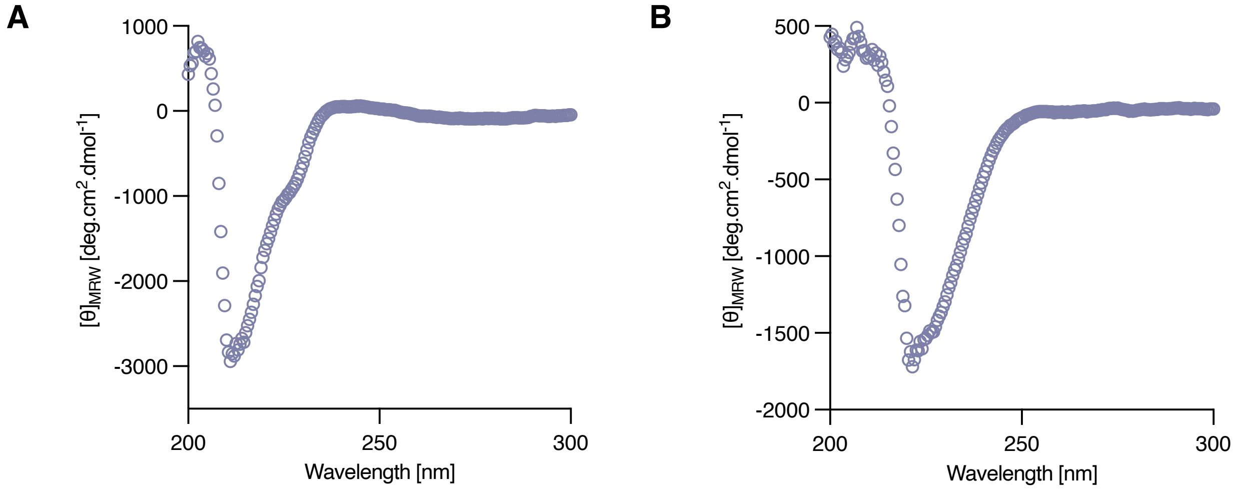

Figure S15. (A) CD spectrum of recombinant βNGF (PeproTech) in 50 mM sodium phosphate buffer at pH 7.0. (B) CD spectrum of synthetic βNGF **6** in 10 mM potassium phosphate buffer at pH 5.0.

12 Protein Sequences

Protein	Sequence
Segment **1**-*GyrA-His*$_{6}$	**M**AEPHSESNV PAGHTIPQAH WTKLQHSLDT ALRRARSAPA AAIAARVAGQ TRNITVEPRL FKKRRLRSPR VLFSTQPPRE AADTQDLDFE VGGAAPFNRT HRSKRSSSHP IFHRGEF*CIT GDALVALPEG ESVRIADIVP GARPNSDNAI DLKVLDRHGN PVLADRLFHS GEHPVYTVRT VEGLRVTGTA NHPLLCLVDV AGVPTLLWKL IDEIKPGDYA VIQRSAFSVD CAGFARGKPE FAPTTYTVGV PGLVRFLEAH HRDPDAQAIA DELTDGRFYY AKVASVTDAG VQPVYSLRVD TADHAFITNG FVSHALEHHH HHH*
Rh-proNGF	**M**AEPHSESNV PAGHTIPQAH WTKLQHSLDT ALRRARSAPA AAIAARVAGQ TRNITVDPRL FKKRRLRSPR VLFSTQPPRE AADTQDLDFE VGGAAPFNRT HRSKRSSSHP IFHRGEFSVC DSVSVWVGDK TTATDIKGKE VMVLGEVNIN NSVFKQYFFE TKCRDPNPVD SGCRGIDSKH WNSYCTTTHT FVKALTMDGK QAAWRFIRID TACVCVLSRK AVRRA

Methionine marked in bold processed following translation. Interphase between fusion proteins indicated with italics.

13 NMR Spectra

1-(Cyanomethyl)pyridin-1-ium bromide ¹H NMR

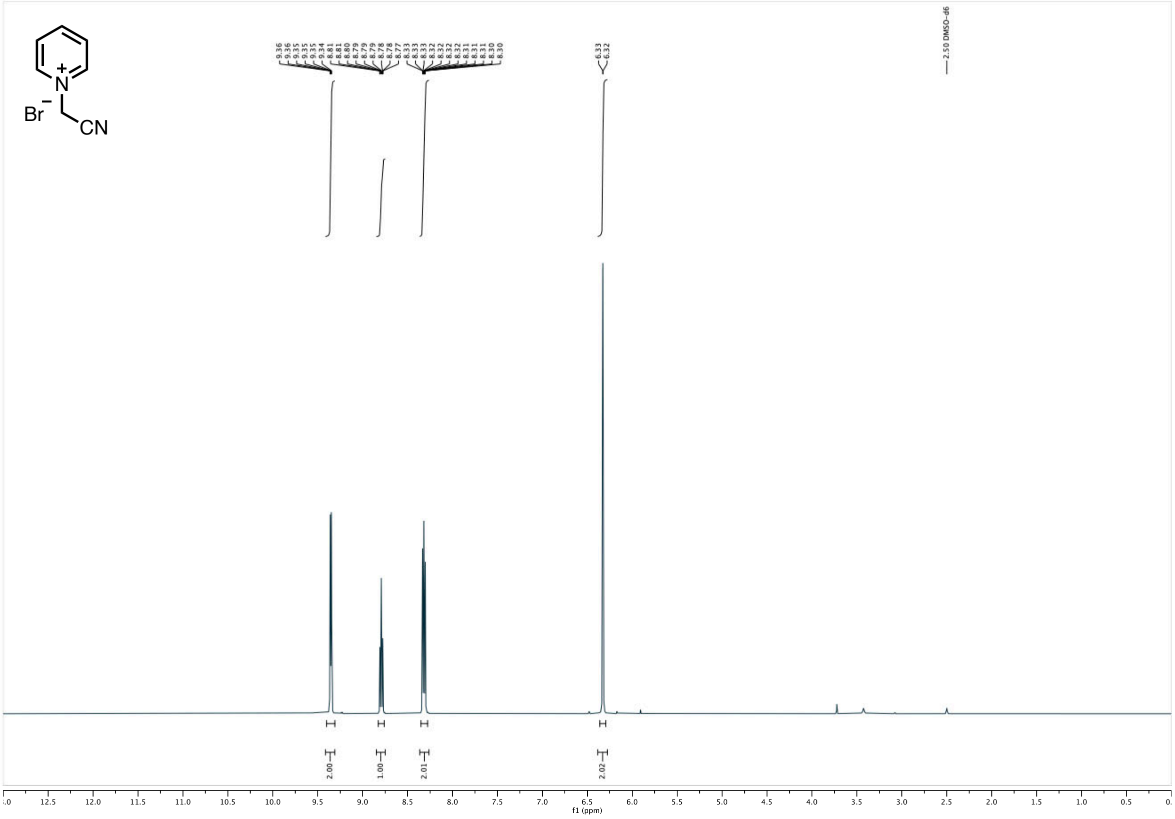

1-(Cyanomethyl)pyridin-1-ium bromide ¹³C NMR

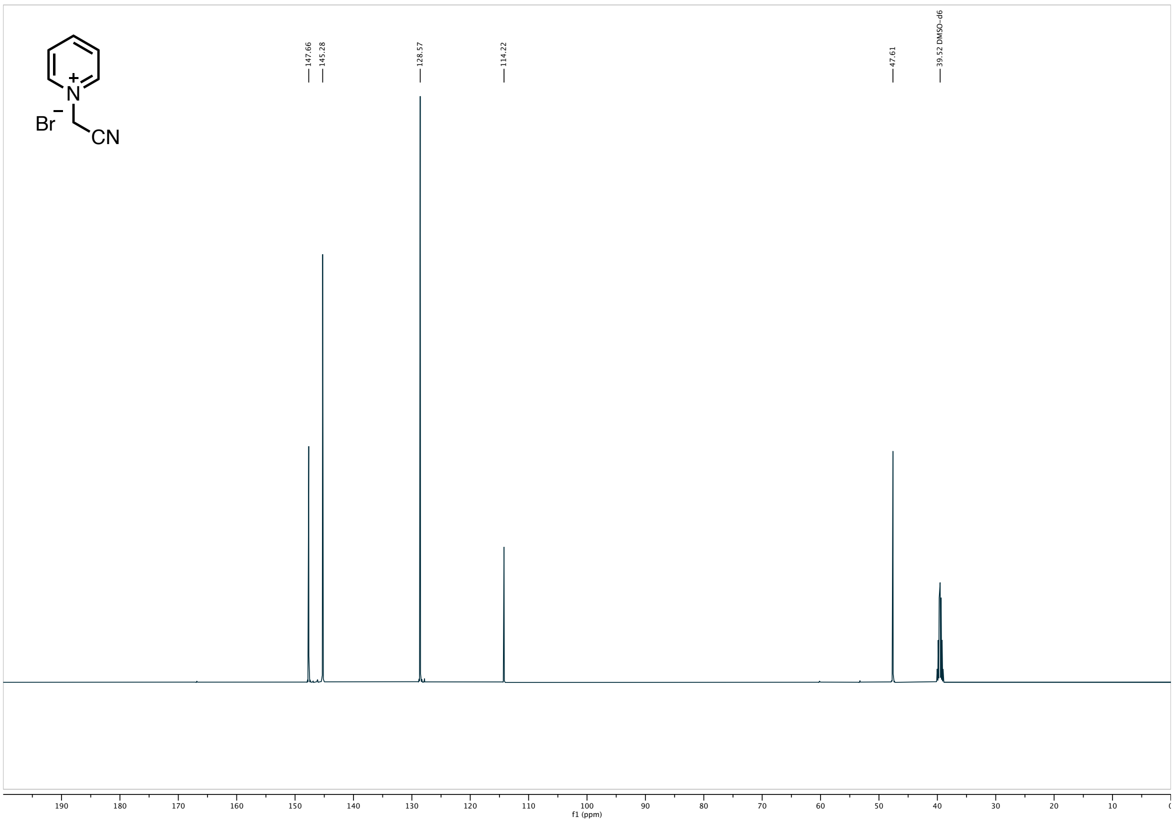

S4 ^{1}H NMR

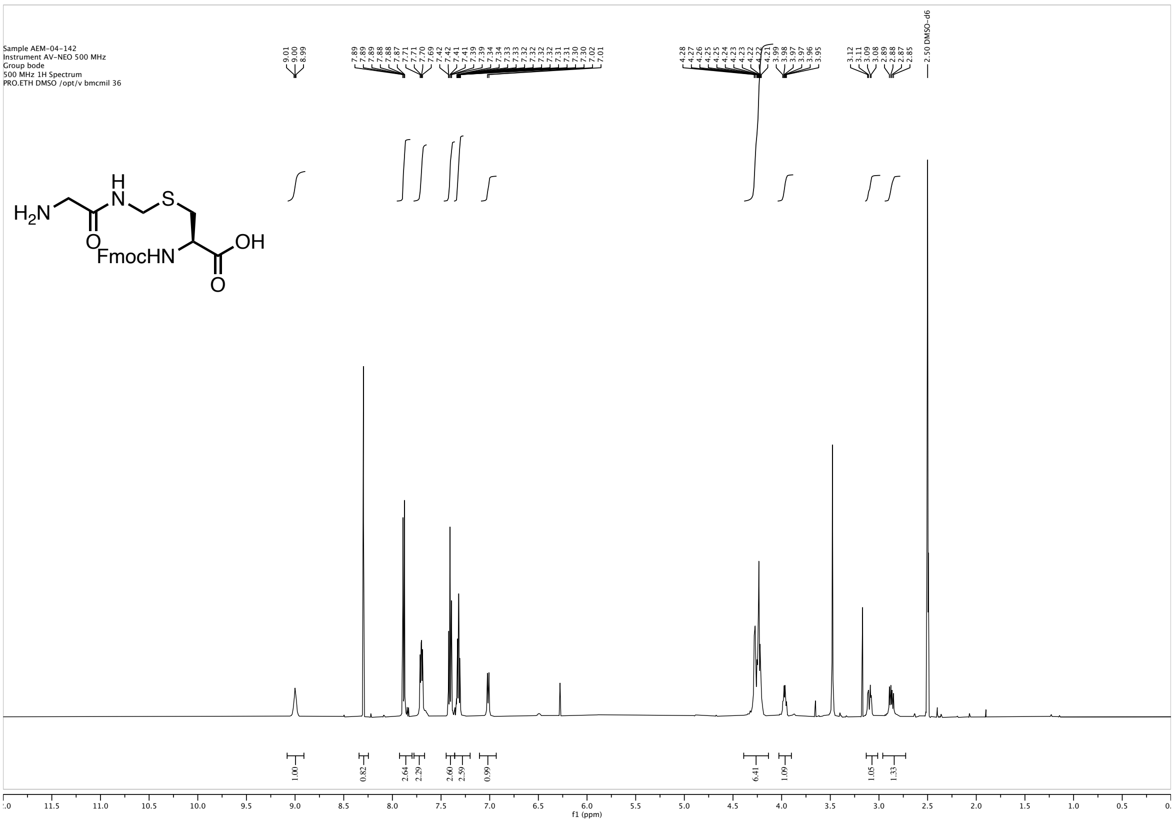

S4 ^{13}C NMR

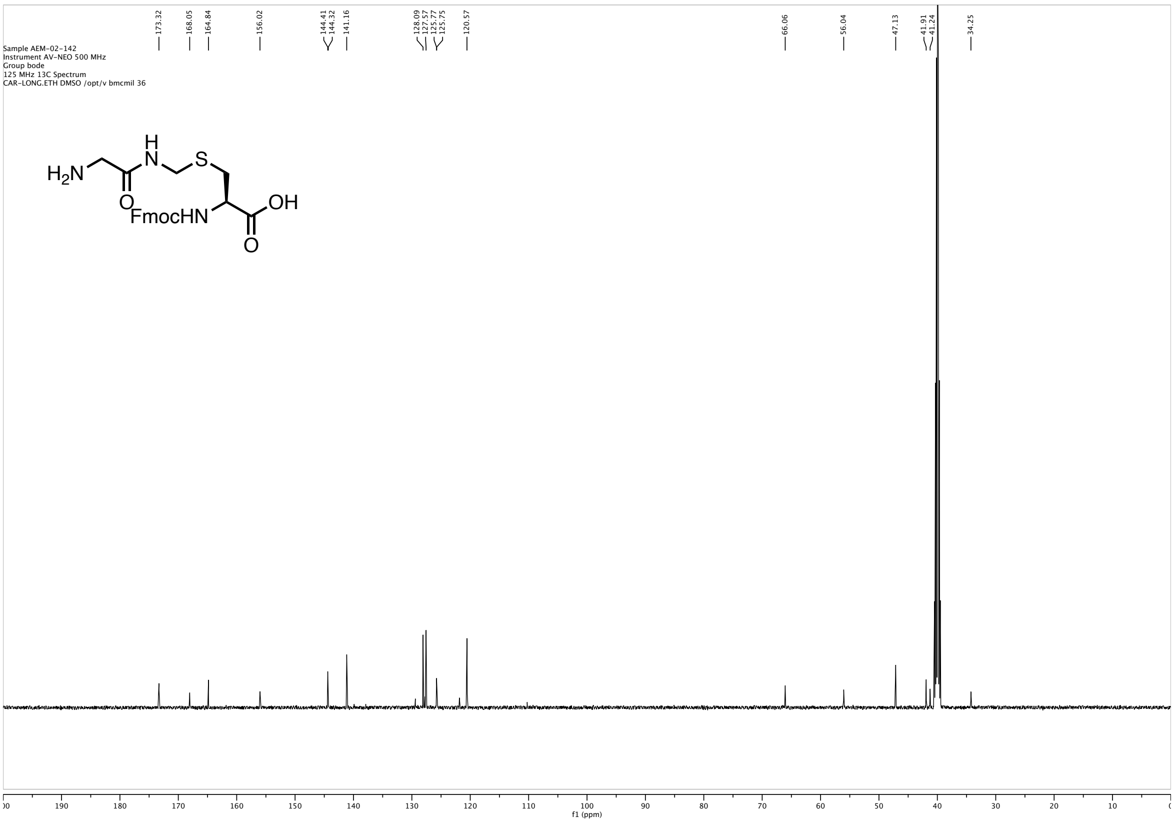

S2a ^{1}H NMR

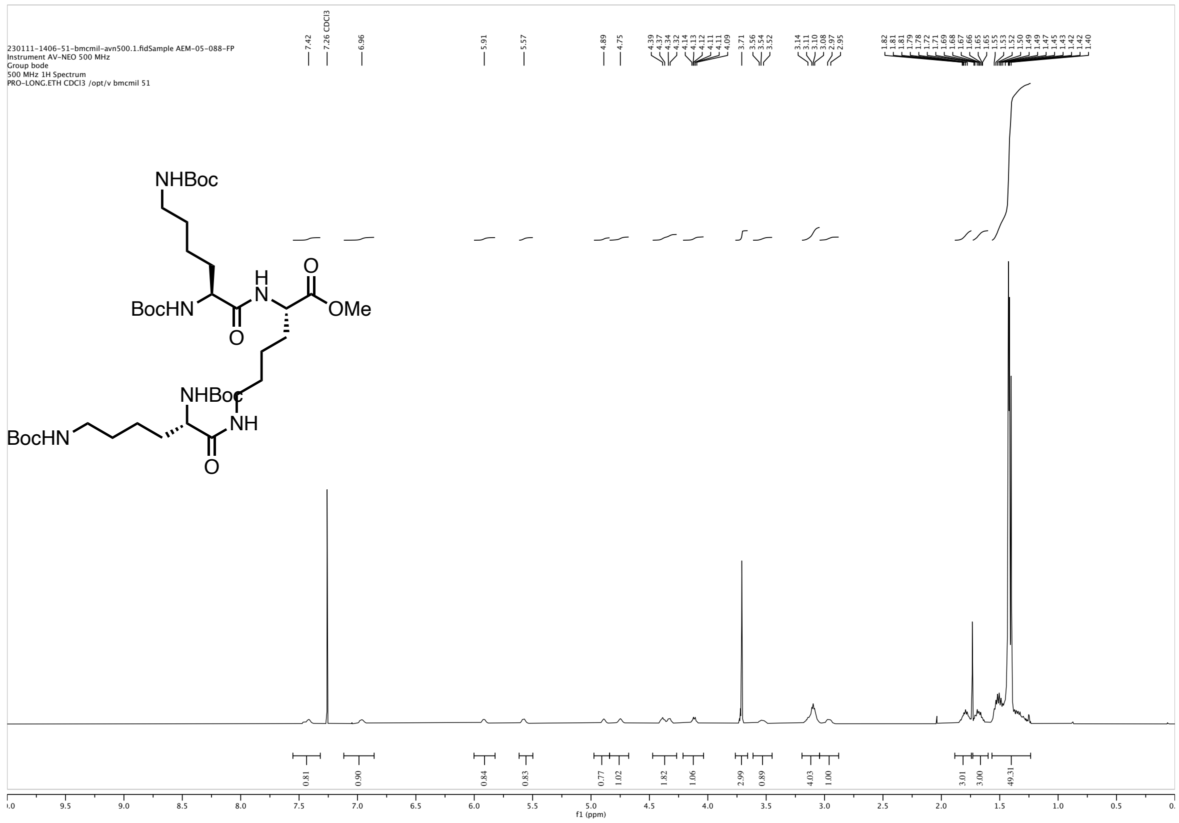

S2a ^{13}C NMR

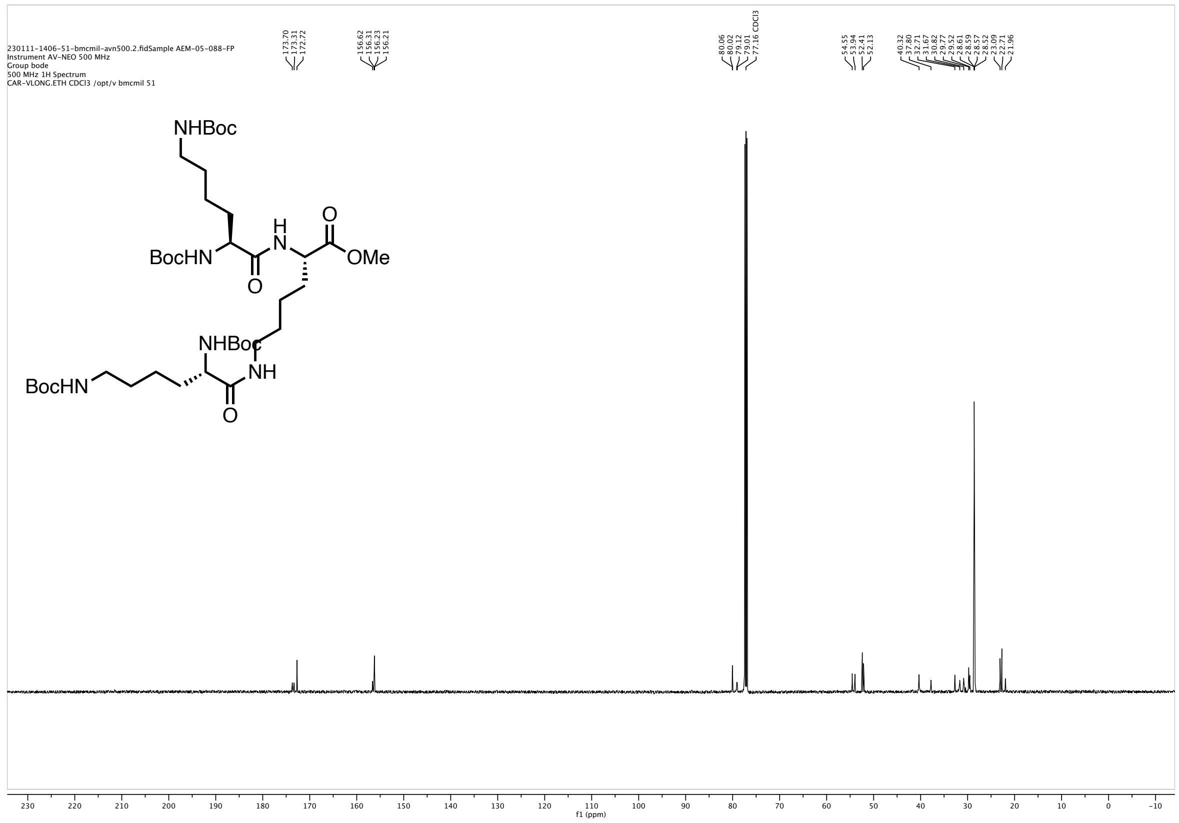

S2b ¹H NMR

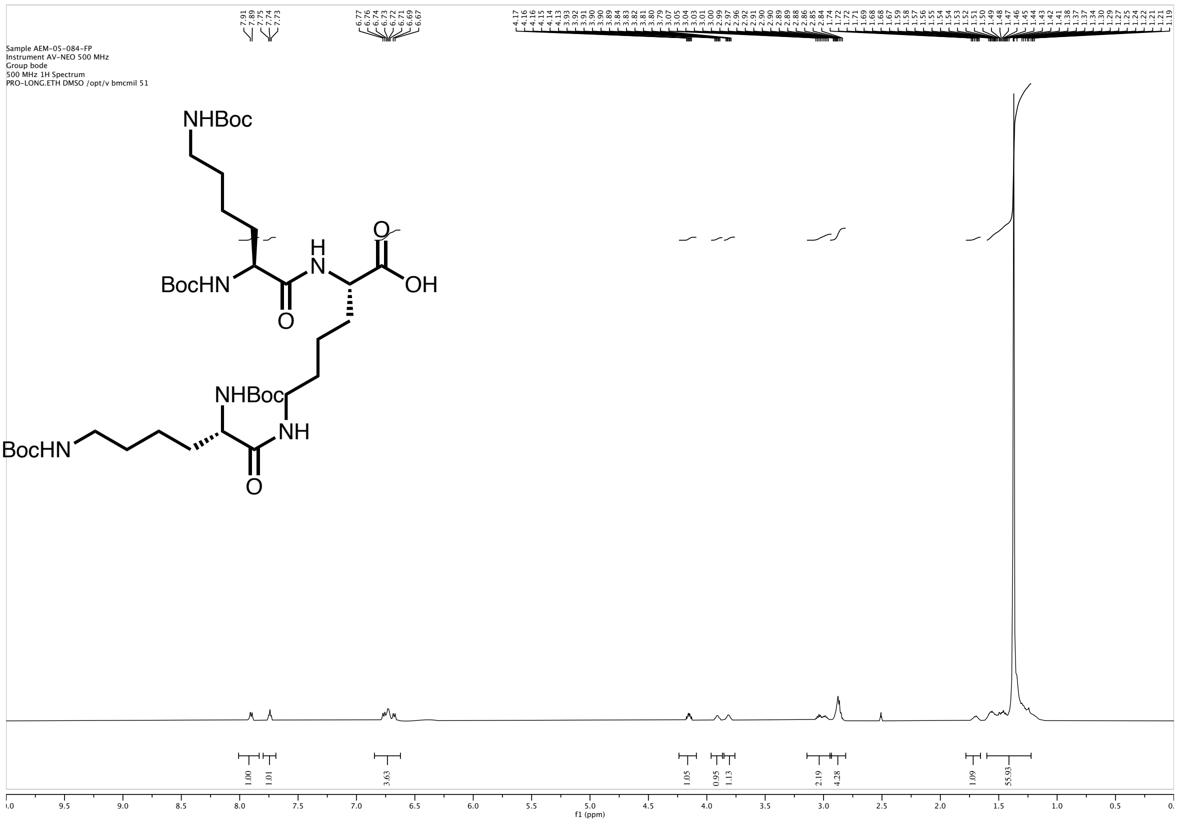

S2b ¹³C NMR

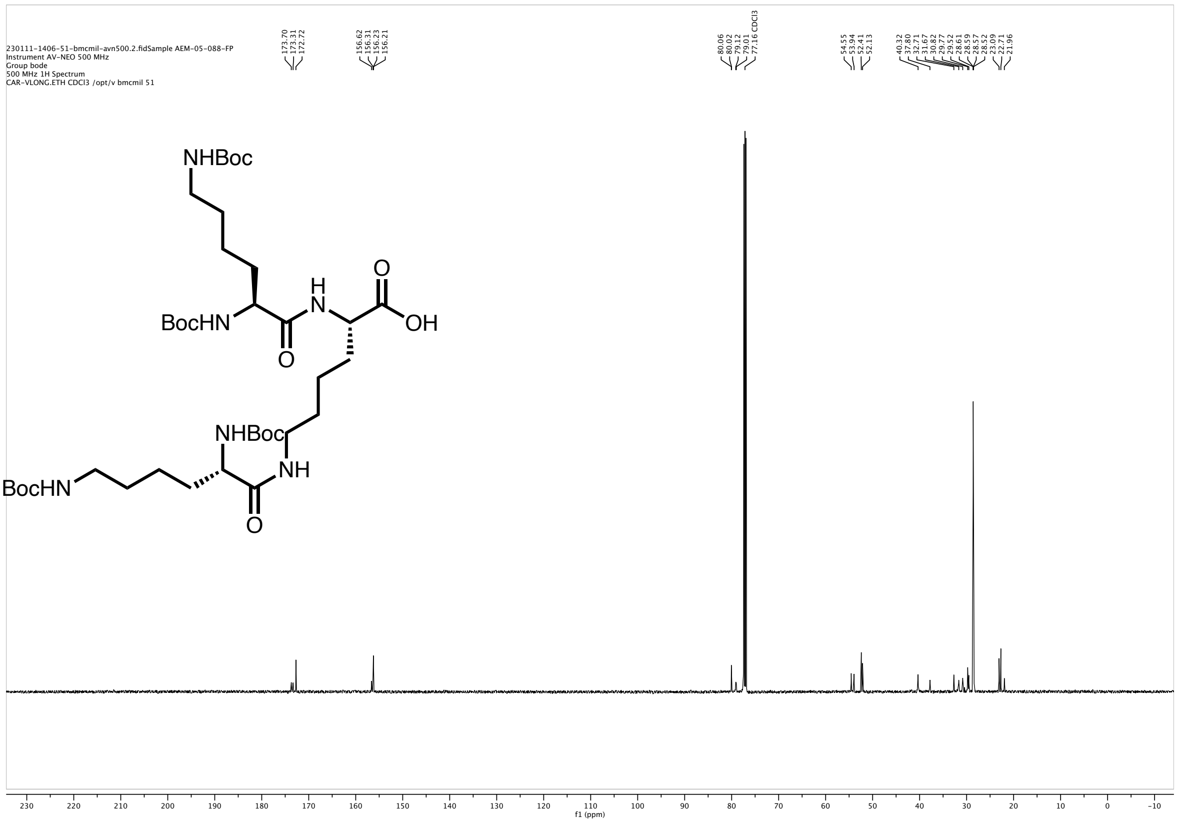

S5 ¹H NMR

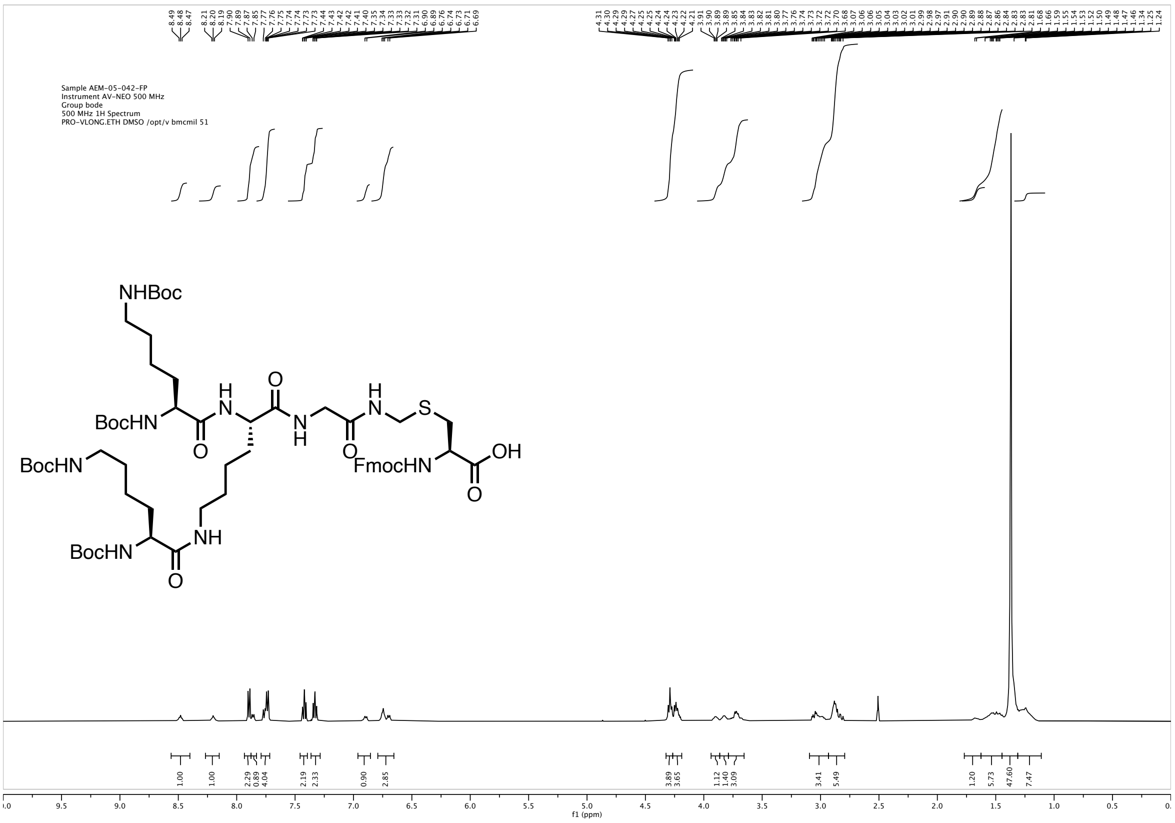

S5 ¹³C NMR

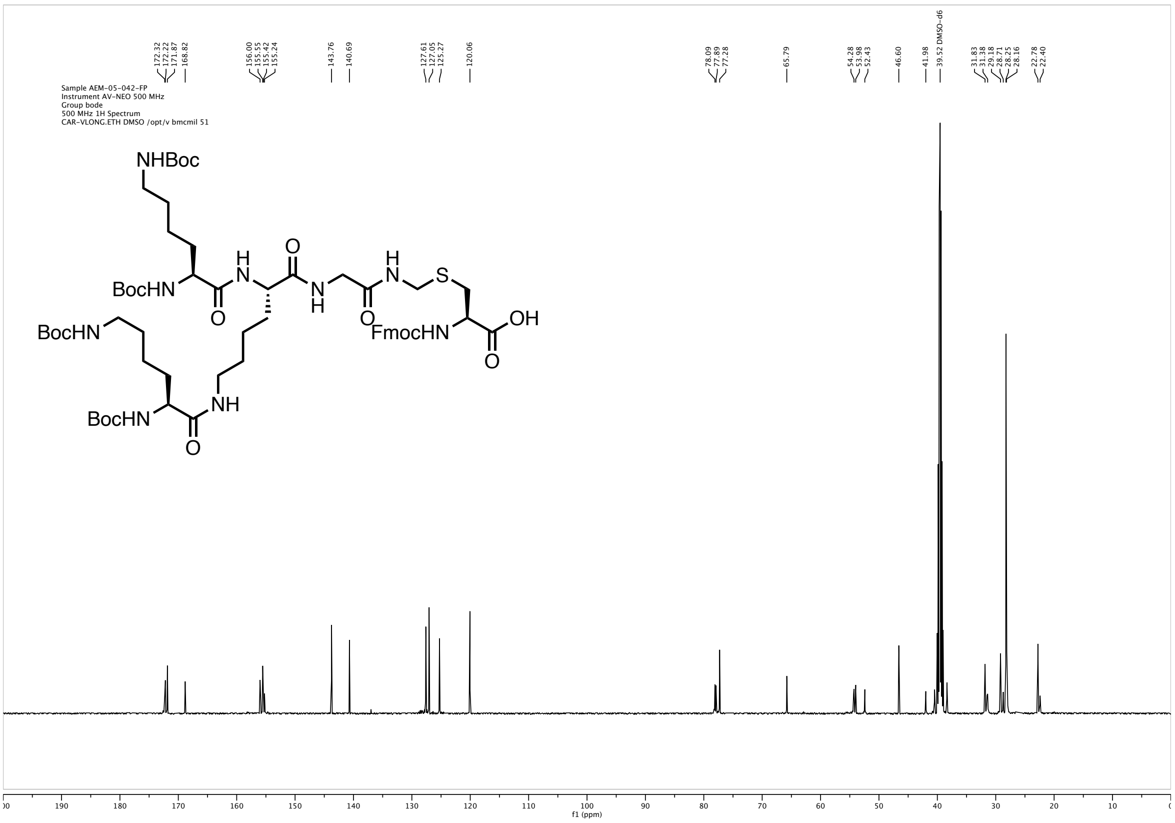

Supplement: Supplementary file 1 [file oc5c00277_si_001.pdf]
