# Supplementary material for: Chemical Synthesis and Chaperone Peptide Mediated Folding of Human Nerve Growth Factor by Expressed KAHA Ligation
Source: ACS Cent Sci. 2025 May 1;11(8):1321–8. doi: 10.1021/acscentsci.5c00277 (PMC12395302; doi:10.1021/acscentsci.5c00277)
Supplement: Supplementary file 2 [file oc5c00277_si_002.pdf]

oc-2025-00277n.R1

Name: Peer Review Information for "Chemical Synthesis and Chaperone Peptide Mediated Folding of Human Nerve Growth Factor by Expressed KAHA Ligation"

First Round of Reviewer Comments

Reviewer: 1

Comments to the Author

Nerve Growth Factor (NGF) is a potent neurotrophic protein effective against central nervous system diseases, but its use is hampered by side effects like hyperalgesia due to pleitropic receptor binding. Chemical synthesis of NGF is challenging because of its small size (13 kDa), the long N-terminal "chaperone peptide" (116 aa) for folding, and its hydrophobic nature. In this manuscript, the authors reported a successful chemical synthesis of NGF variants using α-ketoacid–hydroxylamine (KAHA) ligations. It involves recombinant production of the chaperone peptide and its chemoselective conversion to a C-terminal α-ketoacid. The synthetic NGF variant was assembled from three synthetic and one recombinant segment with the help of a novel solubility tag, SOLACE, and ester-forming KAHA ligations. Controlled folding, disulfide bond formation, and proteolytic cleavage yielded active synthetic NGF as a noncovalent dimer. The synthetic NGF matched the activity of recombinant NGF in axon growth assays, offering a platform for engineering tailored NGF variants. This method provides a versatile pathway for semi-synthesizing other neurotrophins and related proteins requiring long chaperone peptides for correct folding. It is suitable for publication in ACS Central Science , provided the following comments are addressed.

Comments:

1. The author developed a new solubilization strategy, SOLACE. Compared to previously reported strategies for solubilization, such as removable backbone modification and the Brik’s Cys-based method, what are the advantages of this new approach?

2. Recently, glycosylation as an artificial chaperone to assist in the folding of disulfide-bonded proteins was developed. Did the authors consider to use this strategy for the synthesis of β-NGF, which could avoid the introduction of a long chaperone peptide?

3. It was noted that the authors used RP-HPLC to purify the folded products. What were the elution conditions for HPLC, and would denaturation occur under these conditions?

4. It is noteworthy that trypsin was used in the final step to remove the chaperone peptide, which might hydrolyze backbone amides after K/R residues. It is observed that the synthetic beta-NGF contains several K/R residues. Why did trypsin not degrade it?

5. The citation format is inconsistent and needs to be corrected.

Reviewer: 2

Comments to the Author

Nötel et al. report the chemical synthesis of semi-synthetic human nerve growth factor (hNGF) through the ketoacid hydroxylamine (KAHA) ligation of a chaperone peptide containing a C-terminal ketoacid with synthetic hNGF made via iterative KAHA ligations, to obtain properly folded, semi-synthetic pro-hNGF. This pro-polypeptide is then enzymatically treated to yield active hNGF and is shown to be physiologically active when compared to a recombinant standard. The key technological advances in this manuscript are as noted: 1) the development of a synthetic method for obtaining a C-terminal ketoacid from a recombinantly sourced C-terminal thioester 2) a new removable solubility tag for peptide synthesis on aggregation prone regions of their hNGF segment 2 and 3) successful KAHA ligation of two synthetic polypeptide segments containing more than 100 amino acid residues each.

While this manuscript is a tour-de-force in KAHA ligation-based protein semi-synthesis, this reviewer feels that the manuscript fails to seize an opportunity to distinguish itself from recombinant methods. As the manuscript is currently presented, there appears to be no biochemical advantage in using KAHA ligation for the production of designer neurotrophins. To further burnish the strengths of this KAHA ligation strategy, this reviewer suggests installing a functional post-translational modification or some unnatural amino acid (or biophysical/biochemical moiety) to probe the binding interactions of NGF to either

TrkA or p75 (as alluded to in the introduction). While the reviewer appreciates the work involved in making such an analog, the addition of such data now that the synthetic scheme is in place would greatly increase the impact of the work among the broader readership of this journal.

Notes and suggestions:

1) There are two peaks in the MS provided for cyanopyridinium ylide 1b (bottom of Figure 2), owing to the +CyP+ cation. The authors should note this in the figure.

2) The overoxidation of 1b to 1c seems to be the limiting step in the production of "recombinant" ketoacid and is likely the major byproduct in this reaction. Can the authors provide an estimated yield of this oxidized product?

3) The iterative KAHA ligation steps in Figure 3 seem to result in either unreacted starting material or some byproducts. Can the authors provide some comment as to the identity of these species?

4) The lack of a convincing intact mass spectrum for the folded pro-hNGF (5c) is a bit concerning. The MaxEntX deconvoluted spectrum shown in the main figure 4 is a bit misleading when one looks at the raw parent ESI spectra in the SI Figure 10. Have the authors tried using alternative methods such as MALDI-TOF to obtain an intact mass spectrum?

Reviewer: 3

Comments to the Author

In this work Bode and co-workers reported the chemical synthesis of the hydrophobic human NGF, featuring two enabling technologies, i.e., expressed KAHA ligation and the SOLACE tag. To the best of my knowledge, excitingly, this is the first time the KAHA ligation can be carried out with recombinantly expressed protein segment, which for sure can greatly improve the synthetic efficiency if one wants to use this ligation chemistry for protein synthesis. The manuscript is clearly written and all of the arguments are well supported, I thus support its publication in ACS Central Science given that the following issues being addressed:

1) In transferring the expressed protein thioester to its CyPY derivative, would the C-terminal amino acid affect the reaction outcome? Is it generally applicable and have the authors tried the reaction at other sites?

2) In the following Oxone oxidation, the authors observed oxidation at Trp20, are there any other amino acids susceptible to oxidation during this process, like Cys or Met?

3) In this specific case, do you think the oxidation at Trp20 may affect the following folding or proteolytic cleavage? And if not, one may skip the purification of this form?

4) The authors used trypsin for "partial" cleavage of the chaperone peptide, how is it controlled to avoid cleavage at other site?

5) Double check the numbering in HPLC traces for ligations 1-3 in Fig.3, e.g. purified 5/6/7a., which disagree with the text numbering.

SI File:

1) Page 30, Figure 10-I, is the initial Met also covered in the MS/MS analysis? It may be necessary to include it in the sequence.

2) Page 31, Figure 11-E, which is the denatured and the folded NGF trace? Also, in F, "beat-NGF" should be "beta-NGF"

3) Page 39, why are the CD experiments for the recombinant and synthetic proteins carried out in different buffers at different pH?

4) Double check the references in the SI whether they are the same, like refs 6 and 7, refs 10, 11 and 13.

Reviewer: 4

Comments to the Author

Nötel and co-workers report the semisynthesis and chaperone-mediated folding of a 13 kDa Nerve Growth Factor (NGF) variant. This synthetic platform will enable access to analogues to improve the pharmacological properties of NGF (e.g., eliminate off-target effects such as hyperalgesia). Linear NGF is assembled via KAHA ligation from a recombinant chaperone pro-peptide which is essential for folding, and chemically

synthesised fragments of the mature protein. Folding ensures, followed by enzymatic cleavage of the pro-peptide to produce the mature dimeric NGF analogue.

This work is thorough, well-presented, novel, and I commend the authors for their synthetic achievements. Moreover, two key advances in protein synthesis are reported: (i) the production of a recombinant precursor with an α-ketoacid at the C-terminus (to facilitate KAHA ligation), and (ii) the development of a removable solubilising tag on cysteine (SOLACE). Therefore, given these innovations, and the synthetic feat in producing a mature, biologically active NGV dimer, I recommend publication with only minor revisions (see below).

Page 3, line 19. In the SOLACE structure, one of the lysines appears to be Boc-protected ornithine. This mistake also appears in the SI (Section 4).

Page 4, line 7. According to UniProt (P01138), NGF has 241 amino acids, with the first 17 residues omitted in the SI figure. I am wondering whether the numbering should be altered, such that Ala1 becomes Ala18 and so on. This is not a critical issue, but consistency with UniProt and other protein databases would be ideal.

Page 6, line 3. It would be helpful to the reader to show the disulfide connectivity. Or at least sketch in the disulfide connectivity in Section 2 of the SI.

Page 6, line 22. Was there any significant hydrolysis of the thioester observed at pH 8.2, during formation of 1b?

Page 7, line 10. Was there any difficulty in coupling valine (which is β-branched) onto Cys(SOLACE)? Was double coupling or high temperature required? This will be useful information to know as this appears to be a difficult coupling (due to significant steric hindrance).

Page 11, line 38. A brief paragraph on the limitations of this approach for preparing NGF is warranted (e.g., non-native residues, low yield). Moreover, some suggestions for improving the synthesis would be welcome (e.g., improving the crude quality of the peptide precursors, and whether there are key reactions that could be optimised further).

Reviewer: 5

Comments to the Author

In this paper, Bode and co-workers report the synthesis of bioactive Nerve Growth Factor (NGF) using a new method called 'expressed KAHA ligation. NGF has a chaperone segment that assists in its folding within a cell, and after the folding process, the chaperone segment is cleaved by a protease. However, it is known that the entire NGF polypeptide, including the chaperone segment, cannot be expressed in E. coli.

To address this problem, the authors demonstrated a new strategy for the synthesis of the NGF domain. They used E. coli expression of the chaperone segment and then combined it with the NGF domain prepared by a chemical method through KAHA ligation. The KAHA ligation successfully yielded a whole segment consisting of the chaperone segment and the NGF segment. Subsequent folding experiments and proteolysis yielded folded NGF in good yield.

The key advancement in their chemistry is the preparation of a chaperone segment with an α-keto amino acid at its C-terminus through E. coli expression and chemical modification. The authors prepared the chaperone segment in its thioester form using an intein system. Then, they converted it into the α-keto form through a reaction with cyano sulfur ylide. The authors successfully established a new method for preparing the KAHA ligation segment through an E. coli expression method. This is a very interesting idea. By fully utilizing expressed KAHA ligation and a lysine-based solubility tag (SOLACE), they ultimately achieved the synthesis of bioactive NGF.

This reviewer recommends publication in ACS Central Science after minor revisions.

Although the new KAHA ligation strategy is very interesting, the authors prepared a thioester first. However, this means that these peptides could be coupled using conventional native chemical ligation (NCL). In this case, the junction involved a thioester and cysteine, followed by subsequent oxidation using the B. Davis method, which

successfully yielded serine from cysteine. A broad readership may wonder why KAHA ligation was chosen over NCL. This referee acknowledges that hydrophobic protein ligation often requires organic solvents and sometimes high-temperature reaction conditions. Under these circumstances, KAHA ligation is an excellent alternative. This referee recommends that the authors include this discussion.

After the 1st and 2nd KAHA ligation, they cleaved the Fmoc by treatment with HNEt2. Under this basic condition, was O-to-N shift to form an amide bond observed?

・For the conversion of thioester to α-ketoacid, they applied basic condition (pH = 8.2). If they have information about epimerization under these conditions, they should mention that.

・On page 5, line 20, "hydrolyses" should be "hydrolyzed".

・In Figure 3, the compound numbers in HPLC profile, for example, Purified 5, 6 and 7a, should be corrected.

Author's Response to Peer Review Comments:

Dear Editor,

Thank you for the prompt and constructive review of our manuscript. Please find the point-by-point responses and changes made to the manuscript attached. We have also included a version of the main text and Supporting Information with highlights of the revisions made. We hope these will address the concerns of the reviewers.

Best wishes,

Jeff

Jeffrey Bode
Department of Chemistry and Applied Biosciences
ETH Zürich
bode@org.chem.ethz.ch

Zürich, 24 March 2025

Point-by-Point Response

Dear Reviewers,

We appreciate the detailed feedback provided and are grateful for the opportunity to revise the manuscript. Below, we have addressed the points raised in the review and provide clarifications and revisions. We hope these changes meet the expectations of the reviewers and further improve the quality of the manuscript.

Reviewer 1

1. *The author developed a new solubilization strategy, SOLACE. Compared to previously reported strategies for solubilization, such as removable backbone modification and the Brik's Cys-based method, what are the advantages of this new approach?*

   Brik's cysteine-based solubility tag would have been an outstanding solution. We used SOLACE in this case simply because we had begun its development years ago (in 2015) for a different project and resurrected it (including materials on hand) when we ran into difficulties with this synthesis of NGF.

2. *Recently, glycosylation as an artificial chaperone to assist in the folding of disulfide-bonded proteins was developed. Did the authors consider to use this strategy for the synthesis of β-NGF, which could avoid the introduction of a long chaperone peptide?*

   We did not investigate glycosylation as an artificial chaperone for the synthesis of βNGF. The existing literature on NGF makes a strong case that the presence of the chaperone peptide is essential for proper folding, and we therefore prioritized this strategy. We did attempt to fold βNGF without a chaperone peptide, but this approach proved unsuccessful. Given the complex disulfide bond arrangement in βNGF, its correct folding is highly dependent on the precise formation of these bonds.

3. *It was noted that the authors used RP-HPLC to purify the folded products. What were the elution conditions for HPLC, and would denaturation occur under these conditions?*

   The folded proNGF was purified by RP-HPLC using a C4 semi-preparative column with a gradient of H_2O and CH_3CN, both containing 0.1% TFA. Further details on the method and instrument can be found in the supporting information. We have found that these non-reducing conditions maintain the integrity of the disulfide bonds and preserve the native conformation of the protein. The fact that proNGF has three disulfide bonds and compact structure makes it exceptionally stable even to HPLC conditions.

4. *It is noteworthy that trypsin was used in the final step to remove the chaperone peptide, which might hydrolyze backbone amides after K/R residues. It is observed that the synthetic beta-NGF contains several K/R residues. Why did trypsin not degrade it?*

   It is a well-established method to cleave the chaperone peptide in proNGF using trypsin rather than the endogenous enzyme furin. Mature βNGF is relatively stable against trypsin degradation due to its cysteine knot structure that shields potential cleavage sites from the enzyme. Additionally, we carefully optimized the experimental conditions, including reaction time and the enzyme-to-substrate ratio, to ensure selective cleavage of the chaperone peptide while preserving the integrity of the mature βNGF product. The advantage of using trypsin lies not only in its efficient removal of the chaperone peptide but also in its complete digestion, thereby simplifying the subsequent purification process.

5. *The citation format is inconsistent and needs to be corrected.*

   We have corrected the citation format.

Reviewer 2

C. As the manuscript is currently presented, there appears to be no biochemical advantage in using KAHA ligation for the production of designer neurotrophins. To

further burnish the strengths of this KAHA ligation strategy, this reviewer suggests installing a functional post-translational modification or some unnatural amino acid (or biophysical/biochemical moiety) to probe the binding interactions of NGF to either TrkA or p75 (as alluded to in the introduction). While the reviewer appreciates the work involved in making such an analog, the addition of such data now that the synthetic scheme is in place would greatly increase the impact of the work among the broader readership of this journal.

The primary goal of this manuscript is to develop a robust chemical synthesis of βNGF and confirming its biological activity, addressing the challenge of correctly folding the protein using an expressed chaperone peptide. The KAHA ligation approach does introduce a few small mutations, and it was essential to establish that our "wild type" structure could be properly folded, processed, and exhibits biological activity. We fully intend to build upon this work by introducing non-canonical side chains aimed at improving receptor selectivity, particularly toward TrkA over p75. We will also definitely introduce permanent groups to improve it solubility and provide handles for bioconjugation.

1. *There are two peaks in the MS provided for cyanopyridinium ylide 1b (bottom of Figure 2), owing to the +CyP+ cation. The authors should note this in the figure.*

   We will more clearly indicate the adducts in Figure 2.

2. *The overoxidation of 1b to 1c seems to be the limiting step in the production of "recombinant" ketoacid and is likely the major byproduct in this reaction. Can the authors provide an estimated yield of this oxidized product?*

   The overoxidized by-product is likely the species resulting from additional oxidation at Trp20. This byproduct was isolated as a mixture with the desired compound in 16% yield.

3. *The iterative KAHA ligation steps in Figure 3 seem to result in either unreacted starting material or some byproducts. Can the authors provide some comment as to the identity of these species?*

   The KAHA ligation HPLC traces in Figure 3 show unreacted starting materials alongside the desired products. KAHA ligation generally performs best at concentrations between 10 to 20 mM. The unreacted starting materials are likely the

result of kinetic limitations and the formation of gels from the exceptionally hydrophobic segments, which prevented their complete conversion. We expect the overall conversion will improve in future work with segments bearing PEGs or other modifications.

4. *The lack of a convincing intact mass spectrum for the folded pro-hNGF (5c) is a bit concerning. The MaxEntX deconvoluted spectrum shown in the main figure 4 is a bit misleading when one looks at the raw parent ESI spectra in the SI Figure 10. Have the authors tried using alternative methods such as MALDI-TOF to obtain an intact mass spectrum?*

   Mass spectra of proNGF and βNGF are rarely reported due to the significant challenges associated in obtaining them, particularly because salts can interfere with the analysis. Despite extensive efforts we and others could not resolve this problem using MALDI-TOF. ProNGF is thought to form strong interactions with salts, as evidenced by the CyP adducts shown in Figure 2, which complicates the ionization process. The ESI spectrum, which confirms the formation of three disulfide bonds, was obtained following extensive desalting of proNGF with MilliQ water. The molecular masses of both proNGF and βNGF were further validated by Western blot analysis. We are presently in the process of synthesizing βNGF with a bioconjugation handle, which will enable the incorporation of an ionization tag to facilitate detection by MS.

Reviewer 3

1. *In transferring the expressed protein thioester to its CyPY derivative, would the C-terminal amino acid affect the reaction outcome? Is it generally applicable and have the authors tried the reaction at other sites?*

   The C-terminal amino acid may influence the reaction kinetics. However, beyond the chaperone peptide, which has a C-terminal phenylalanine, the reaction has also been successfully performed on a ubiquitin derivative with a C-terminal arginine. In that case, no significant differences in reaction outcome or conditions were observed.

2. *In the following Oxone oxidation, the authors observed oxidation at Trp20, are there any other amino acids susceptible to oxidation during this process, like Cys or Met?*

   The chaperone peptide lacks both cysteine and methionine, residues that would likely be sensitive to the oxidation conditions. We are currently developing strategies to overcome this limitation, particularly for cysteine, which could potentially be protected, or by buffering the reaction mixture, as was done for tryptophan.

3. *In this specific case, do you think the oxidation at Trp20 may affect the following folding or proteolytic cleavage? And if not, one may skip the purification of this form?*

   While tryptophan oxidation can influence protein structure and binding, it unlikely that the oxidation of Trp20 would affect the folding of proNGF, as the “chaperone peptide” is largely unstructured. For this initial report on βNGF synthesis, we elected to remove the side product with oxidized Trp20 as it could be effectively separated from the desired product via RP-HPLC. In future studies, we may prepare proNGF containing oxidized Trp20 to facilitate material throughput and assess any potential impact on its structure and function.

4. *The authors used trypsin for "partial" cleavage of the chaperone peptide, how is it controlled to avoid cleavage at other site?*

   It is a well-established technique to cleave the chaperone peptide in proNGF using trypsin rather than the endogenous enzyme furin. The chaperone peptide is structurally disordered, making it more accessible to trypsin, whereas the mature βNGF adopts a tightly packed cysteine knot structure that shields potential cleavage sites, thereby increasing its resistance to degradation. Furthermore, we carefully optimized the experimental conditions, including the enzyme-to-substrate ratio and incubation time, to achieve selective cleavage of the chaperone peptide while preserving the integrity of the final product.

5. *Double check the numbering in HPLC traces for ligations 1-3 in Fig.3, e.g. purified 5/6/7a., which disagree with the text numbering. SI File: 1) Page 30, Figure 10-I, is the initial Met also covered in the MS/MS analysis? It may be necessary to include it in the sequence. 2) Page 31, Figure 11-E, which is the denatured and the folded NGF trace? Also, in F, "beat-NGF" should be "beta-NGF" 3) Page 39, why are the*

CD experiments for the recombinant and synthetic proteins carried out in different buffers at different pH? 4) Double check the references in the SI whether they are the same, like refs 6 and 7, refs 10, 11 and 13.

We have reviewed and corrected the numbering in the HPLC traces. 1) The chaperone peptide was expressed in *E. coli*, and the initiator methionine was processed during expression, so it is not present in the final peptide sequence. 2) Both traces show denatured βNGF, as folding without the chaperone peptide was unsuccessful. The caption for Figure 11-F was revised accordingly. 3) The CD spectrum for recombinant βNGF was measured at an earlier stage of the project. The synthetic material was found to be more stable at lower pH, and by the time the measurements were made, the recombinant βNGF had already been depleted. As a result, remeasuring the CD spectrum for the recombinant protein was deemed unnecessary. 4) We have reviewed and corrected the references as needed.

Reviewer 4

1. *Page 3, line 19. In the SOLACE structure, one of the lysines appears to be Boc-protected ornithine. This mistake also appears in the SI (Section 4).*

   We thank the reviewer for catching this and have corrected the structure of SOLACE.

2. *Page 4, line 7. According to UniProt (P01138), NGF has 241 amino acids, with the first 17 residues omitted in the SI figure. I am wondering whether the numbering should be altered, such that Ala1 becomes Ala18 and so on. This is not a critical issue, but consistency with UniProt and other protein databases would be ideal.*

   The first 17 residues correspond to the signal peptide of proNGF, and collectively they are often referred to as pre-proNGF. This signal peptide is cleaved during translocation into the endoplasmic reticulum, which is why we chose to exclude it from the synthesis. However, we have adjusted the numbering to make it consistent with UniProt P01138.

3. *Page 6, line 3. It would be helpful to the reader to show the disulfide connectivity. Or at least sketch in the disulfide connectivity in Section 2 of the SI.*

   We have now highlighted the disulfide connectivity in Section 2 of the SI.

4. *Page 6, line 22. Was there any significant hydrolysis of the thioester observed at pH 8.2, during formation of 1b?*

   Hydrolysis was the main competing reaction; however, it was effectively minimized through careful optimization of the reaction conditions.

5. *Page 7, line 10. Was there any difficulty in coupling valine (which is β-branched) onto Cys(SOLACE)? Was double coupling or high temperature required? This will be useful information to know as this appears to be a difficult coupling (due to significant steric hindrance).*

   Fmoc-Cys(SOLACE)-OH was manually coupled at room temperature with an extended reaction time. The coupling efficiency was assessed, and double coupling was performed only when deemed necessary.

6. *Page 11, line 38. A brief paragraph on the limitations of this approach for preparing NGF is warranted (e.g., non-native residues, low yield). Moreover, some suggestions for improving the synthesis would be welcome (e.g., improving the crude quality of the peptide precursors, and whether there are key reactions that could be optimized further).*

   We have added a discussion of the limitations of this approach.

Reviewer 5

1. *Although the new KAHA ligation strategy is very interesting, the authors prepared a thioester first. However, this means that these peptides could be coupled using conventional native chemical ligation (NCL). In this case, the junction involved a thioester and cysteine, followed by subsequent oxidation using the B. Davis method, which successfully yielded serine from cysteine. A broad readership may wonder why KAHA ligation was chosen over NCL.*

   The closest Cys residue to the chaperone peptide falls well into the βNGF sequence and would require NCL with a Valine-derived thioester. The alternative, selective desulfurization in the presence of six cysteines presents a significant challenge, requiring additional protecting and deprotecting steps. More importantly, KAHA

ligation could be performed under acidic conditions that proved ideal for solubilizing the exceptionally hydrophobic 25 kDa proNGF. While we have used NCL successfully and with pleasure for more soluble proteins, the requirement for basic, aqueous conditions presents numerous challenges for hydrophobic targets.

2. *After the 1st and 2nd KAHA ligation, they cleaved the Fmoc by treatment with $HNEt_2$. Under this basic condition, was O-to-N shift to form an amide bond observed?*

   As we have previously studied, the O-to-N shift does not occur under the Fmoc deprotection conditions. In general, the O-to-N shift requires aqueous, basic conditions and the Fmoc deprotection occurs much faster than the O-to-N shift.

3. *For the conversion of thioester to α-ketoacid, they applied basic condition (pH = 8.2). If they have information about epimerization under these conditions, they should mention that.*

   We did not specifically investigate epimerization during cyanopyridinium ylide formation, as they are similar to extensive studies on NCL and aminolysis of peptide thioesters. Our group has, however, examined epimerization during the formation of the related cyanosulfonium ylides from activated carboxylic acids and subsequent oxidation to alpha-ketoacids. While these studies were conducted under somewhat different conditions, we observed no significant epimerization.

4. *On page 5, line 20, "hydrolyses" should be "hydrolyzed".*

   Thank you. We have corrected the spelling.

5. *In Figure 3, the compound numbers in HPLC profile, for example, purified 5, 6 and 7a, should be corrected.*

   We have reviewed and corrected the numbering in the HPLC traces.

We thank you again for your thoughtful insights and constructive suggestions.

Sincerely,

Jeffrey Bode, on behalf of all the authors.

oc-2025-00277n.R2

Name: Peer Review Information for "Chemical Synthesis and Chaperone Peptide Mediated Folding of Human Nerve Growth Factor by Expressed KAHA Ligation"

Second Round of Reviewer Comments

Reviewer: 4

Comments to the Author

The authors have addressed the reviewers' queries more than adequately. I congratulate the author son their work. In my view, it can be published without further revision required.

Reviewer: 1

Comments to the Author

The authors have revised the manuscript in accordance with the reviewer's requests, and it is now suitable for publication in ACS Central Science.

Reviewer: 5

Comments to the Author

The paper is well revised.

Reviewer: 3

Comments to the Author

All my previous concerns have been addressed properly, and I support the publication of this manuscript in ACS Cent Sci.

Reviewer: 2

Comments to the Author

The authors have done a reasonable job addressing my comments and those of the other reviewers. That being said, I still have reservations about including the MaxEnt MS type analysis in the characterization Figure since I believe this type of data analysis does not provide a reliable measure of purity for the final product. I think this should be removed from the figure and the reader directed to the SI data. Otherwise, I believe the manuscript is suitable for publication.

Author's Response to Peer Review Comments:

Dear Editor,

Thank you for the further review of our manuscript. Please find our responses to Review 2 and the formatting requests of the Editorial Office attached.

Thank you again for the prompt and professional handling of our manuscript. We look forward to seeing it in print!

Best wishes,

Jeff

Reviewer 2

I still have reservations about including the MaxEnt MS type analysis in the characterization Figure since I believe this type of data analysis does not provide a reliable measure of purity for the final product. I think this should be removed from the figure and the reader directed to the SI data. Otherwise, I believe the manuscript is suitable for publication.

We agree that the MaxEnt analysis has limitations and have removed this from Figure 4 and pointed the reader to the more extensive MS data in the Supporting Information. We have retained the MaxEnt MS spectra in the SI, along with the unprocessed MS data.

Formatting Needs:

Author List: Please include the email address(es) of the corresponding author(s) on the first page of the manuscript.

We have included the email address of the corresponding author on the first page of the manuscript.

Copyrights: Some or all of your graphics/tables includes a reference citation without a credit line. Please confirm that this pertains only to data, and not the figure/table itself."

The references in the Figure captions are to AlphaFold, which was used to generate some of the images. All of the graphics are our original artwork.

Graphics: Your figures and TOC Graphic may not be legible at print size. Please ensure graphics are an appropriate resolution for publication. If any parts of the graphic are unreadable, they should be replaced for better clarity and readability.

We have checked the figures and improved the resolution where necessary. We believe they should be acceptable in the printed version.

Supporting Information: Please label all graphics/tables in the following format: "Figure S1, S2…", "Scheme S1, S2…." or "Table S1, S2…", etc.

Supporting Information: Please number all pages in the following format: S1, S2, S3, etc

All of the Figures in the Supporting Information are now labelled as “Figure S1”, etc. We have updated the page numbers to include “S1, S2 etc.”.
